# Multimodal/Multifunctional Nanomaterials in (Bio)electrochemistry: Now and in the Coming Decade

**DOI:** 10.3390/nano10122556

**Published:** 2020-12-19

**Authors:** Paloma Yáñez-Sedeño, Araceli González-Cortés, Susana Campuzano, José Manuel Pingarrón

**Affiliations:** Departamento de Química Analítica, Facultad de Ciencias Químicas, Universidad Complutense de Madrid, E-28040 Madrid, Spain; aracelig@quim.ucm.es (A.G.-C.); pingarro@quim.ucm.es (J.M.P.)

**Keywords:** multifunctional nanomaterials, electrochemical (bio)sensing, metal nanomaterials, carbon nanomaterials, silica-based nanomaterials

## Abstract

Multifunctional nanomaterials, defined as those able to achieve a combined effect or more than one function through their multiple functionalization or combination with other materials, are gaining increasing attention in the last years in many relevant fields, including cargo targeted delivery, tissue engineering, in vitro and/or in vivo diseases imaging and therapy, as well as in the development of electrochemical (bio)sensors and (bio)sensing strategies with improved performance. This review article aims to provide an updated overview of the important advances and future opportunities exhibited by electrochemical biosensing in connection to multifunctional nanomaterials. Accordingly, representative aspects of recent approaches involving metal, carbon, and silica-based multifunctional nanomaterials are selected and critically discussed, as they are the most widely used multifunctional nanomaterials imparting unique capabilities in (bio)electroanalysis. A brief overview of the main remaining challenges and future perspectives in the field is also provided.

## 1. Introduction

Electrochemical (bio)sensors have attracted considerable attention over the years due largely to their simplicity and sensitivity. These characteristics have led to this type of biosensor to be widely used in a variety of applications where the presence of nanomaterials is essential. As it is well known, these materials provide advantageous properties, leading to an improved (bio)sensing performance of conventional electrodes. Regarding metallic nanoparticles (especially gold nanoparticles), their ability to adsorb biomolecules in a stable way with no loss of bioactivity for a long time was firstly exploited. In the case of carbon nanomaterials (i.e., carbon nanotubes), wide use was made of the intense electrocatalytic activity towards electrochemical reactions in which species such as NADH or H_2_O_2_ were involved. Throughout the last years, other valuable properties such as porosity in silica-based materials, magnetism, or enzymatic activity of metal oxides, have also been explored while other related nanomaterials, i.e., bimetallic nanoparticles, quantum dots (QDs), graphene or graphene QDs, have shown interesting features for application in electrochemical detection. A very interesting aspect is that most of these nanomaterials may exhibit several of these properties and, therefore, they can be considered as multifunctional materials in the sense that they work properly, fulfilling two or more functions simultaneously. For example, nanomaterials able to immobilize bioreagents, improve the electrode conductivity, and exhibit enzyme activity have been used in the construction of biosensing platforms. The preparation of these multifunctional nanomaterials is carried out by incorporating proper functional groups to a single product or, more frequently, by combining various dissimilar nanomaterials exhibiting different properties and activities. Multifunctional nanocomposites combine the desirable features of each particular nanomaterial to form new nanostructures in which, compared to their base counterparts, synergic or enhanced properties can be designed and controlled with high diversity, therefore providing an interesting and unique versatility of use and application.

Although some recent articles have reviewed nicely the potential of functional nanomaterials and nanostructures to improve the performance of electrochemical biosensors [1,2,3], as far as we know, no review has critically overviewed the use of the different types of multimodal/multifunctional nanomaterials in connection with electrochemical biosensing. In this review, multifunctional metal-based nanoparticles, carbon and mesoporous silica nanomaterials, as the most popular in the (bio)sensing field, are considered. The relevant properties exhibited by these materials: magnetism, enzyme activity, uniform porosity, electrocatalysis, or biocompatibility, combined with the suitability for biomolecules immobilization, high conductivity, easy functionalization, stability, and the ability for signal amplification, are discussed and illustrated with selected examples from the recent literature.

## 2. Multifunctional Metal-Based Nanomaterials

Metal and metal oxide nanoparticles have been used extensively for years in the preparation of electrochemical sensors and biosensors. Metal-based nanoparticles have shown to be useful for improving the detection capacity of (bio)sensors, acting in a multifunctional way in transcendental areas related to higher sensitivity (such as signal enhancement by improving the electron transfer rate or increasing the amount of immobilized bioreagents), and better selectivity (i.e., showing electrocatalytic effects toward a variety of electrochemical processes). To enhance (bio)sensing performance, metal-based nanomaterials have been used in two different ways: as modifiers of the electrode surface and as carrier tags. In both cases, the use of the nanomaterials provokes an improvement in the electrochemical response, thus allowing lower detection limits as well as the possibility of analyzing highly complex samples without or after minimal pretreatment.

This section is focused on the multifunctional sensing applications of metal nanoparticles (MNPs), metal oxides, and QDs alone or combined with other materials, as they are likely the most widely used metal-based nanostructures in the (bio)electro-analytical field. In addition, some strategies involving the use of two-dimensional (2D) transition metal chalcogenides (TMCs), transition metal oxides (TMOs), or Mxenes, as representative examples of more recent nanomaterials [4,5] are also discussed.

### 2.1. Metal Nanoparticles (MNPs)

The excellent properties of MNPs, i.e., the large surface area, electrical conductivity, electrocatalytic ability, good biocompatibility, adsorption ability, and pseudo-enzymatic behavior, boosted long ago their use in the preparation of electrochemical biosensors. MNPs have been employed as transducer surface modifiers to both enhance the capacity for the stable and well-oriented immobilization of biomolecules and improve the charge transport rates [6]. Taking advantage of these properties, a variety of electrode scaffolds using MNPs have been designed for the construction of enzyme biosensors [7,8,9,10], immunosensors [11,12,13,14,15,16,17,18,19,20,21,22,23,24,25], aptasensors [26,27,28,29,30,31], and DNA biosensors [32,33,34,35,36,37,38,39,40]. Table 1 summarizes the main characteristic, including the nanomaterial roles, the limits of detection, and the analyzed sample, of the electrochemical biosensing strategies involving multifunctional MNPs.

Focusing on enzyme-based biosensors, the dual role of MNPs acting as biomolecules support and electrocatalytic material for the improved detection of redox substrates or products and, in some cases, acting themselves as artificial enzymes, deserves to be highlighted. Some representative examples to be mentioned include the electrochemical reduction of enzymatically produced H_2_O_2_ at operating potentials close to 0 V in the presence of gold nanoparticles (AuNPs) [8] or platinum nanoparticles (PtNPs) [9], or the decreasing in the oxidation potential of enzymatically produced thiocoline at electrodes modified with AuNPs [41], AuNPs-QDs [42], or AgNPs [43]. Furthermore, it is important to note the ability of MNPs to allow direct electron transfer (DET) between redox proteins and bulk electrode materials, which permits electrochemical detection with no need for electron transfer mediators. As an illustrative example, gold nanotriangles (AuNTs) were used as a surface-modifier of a carbon ionic liquid electrode (CILE) to achieve direct electrochemistry and electrocatalysis of horseradish peroxidase (HRP) [7]. AuNTs exhibited a larger surface area than other AuNPs shapes, thus increasing the HRP loading, and acted as a bridge between the redox sites of the enzyme and the electrode surface. The resulting enzyme biosensor was applied with good results to the detection of trichloroacetic acid and sodium nitrite.

#### 2.1.1. Metal-Based Nanozymes

**Figure 1:**
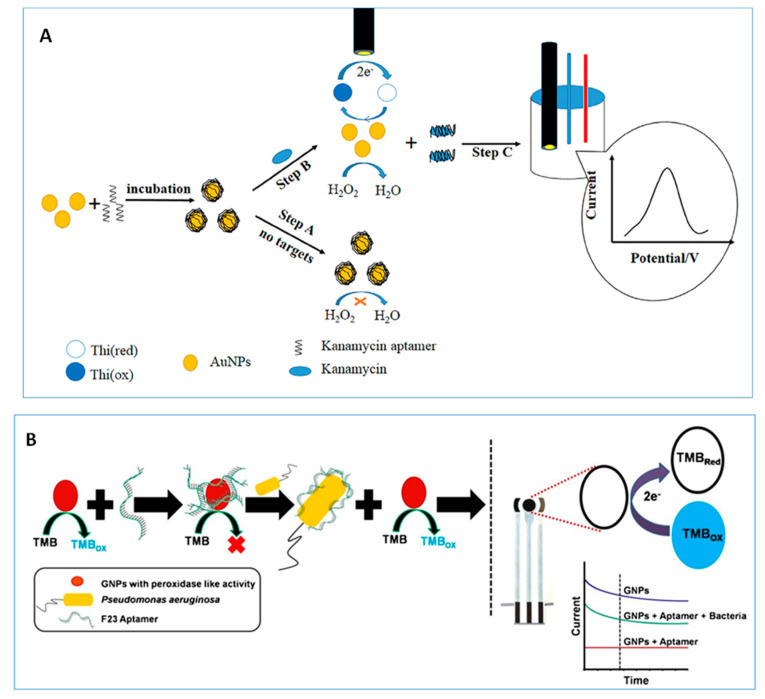
Scheme of aptasensing assays for the detection of kanamycin (**A**) and *P. aeruginosa* (**B**) using inhibition of AuNPs peroxidase activity. Reproduced from [44] (**A**) and [26] (**B**), with permission.

The usefulness of AuNPs as nanozymes has been widely recognized due to their excellent ability to mimic peroxidase activity of natural enzymes [60]. Electrochemical biosensors have been constructed using AuNPs as multifunctional substrates for immobilizing bioreagents and allowing the electrochemical reaction to occur at optimal experimental conditions without using natural enzymes [26,44]. In this context, it should be noted the development of electrochemical aptasensors in which the catalytic activity of AuNPs is exploited for the detection of H_2_O_2_ in the presence of a redox mediator. An illustrative example is an enzyme-free electrochemical aptasensor for the determination of kanamycin involving adsorption of ssDNA onto AuNPs and target induced replacement of the aptamer. Figure 1A shows as in the absence of kanamycin, the catalytic site of AuNPs is blocked and the peroxidase activity inhibited. However, this activity is recovered in the presence of the target providing a good electrochemical response for the reduction of oxidized thionine [44]. The method allowed the determination of kanamycin over the 0.1 to 60 nM range with a detection limit (LOD) of 0.06 nM, and was applied to the analysis of honey samples. In a more recent work, Das et al. [26] reported an aptasensor for the detection of *Pseudomonas aeruginosa* bacteria involving also the aptamer-controlled reversible inhibition of gold nanozyme activity (Figure 1B). In this case, 3,3′,5,5′-tetramethylbenzidine (TMB) was used as a colorimetric and electroactive reagent, allowing amperometric detection on a SPCE at −0.4 V.

Bimetallic nanoparticles exhibit superior biological enzyme-like activity because of the bi-functional effects and the easy shape and composition tunability for improving the properties of the implied nanomaterials [19,27,31,45,61]. Therefore, the applications of these nanoparticles in the construction of electrochemical biosensors have extended rapidly, being some example detailed in Table 1. As a representative example, PtPdNPs were used for the preparation of an aptasensor for the detection of Mucin 1, a heavily glycosylated protein belonging to the mucins family, and overexpressed in various types of cancer. In this configuration, the target-induced catalytic hairpin assembly was combined with the mimic peroxidase performance of PtPdNPs and so, the replacement of the target from the double helix structure on the electrode surface by a longer fragment labeled with PtPdNPs could effectively catalyze the oxidation of TMB by H_2_O_2_ to generate amplified electrochemical signals through a linear range between 100 fg mL^−1^ and 1 ng mL^−1^ with a LOD of 16 fg mL^−1^ [31].

Since the verification of intrinsic peroxidase-like activity of magnetite nanoparticles (Fe_3_O_4_)NPs in 2007 [62], other metal oxide nanomaterials such as cerium (ceria, CeO_2_) [46,63], copper [64], zinc [65], manganese [29], or cobalt [66] oxides, have been reported to show intrinsic enzyme like properties. Various studies have demonstrated that CeO_2_ NPs possess multienzyme properties [25,67], exhibiting the ability to mimic redox enzymes such as peroxidase, oxidase, or catalase among others [68]. The high catalytic performance is attributed to the presence of both Ce^+3^ and Ce^+4^ oxidation states and the existence of oxygen vacancies which make this nanomaterial useful for the preparation of enzyme-free electrochemical biosensors. So, taking advantage of the GOx-mimicking activity of CeO_2_ NPs, Kim et al. developed a simple and label-free strategy for the detection of terminal transferase (TdT), overexpressed in acute lymphoblastic leukemia. A personal glucose meter (PGM)-based DNA detection method involving the tuning of CeO_2_NPs GOx activity by DNA-induced aggregation was developed [46]. While in the absence of TdT, glucose was efficiently catalyzed by nanoparticles, thus decreasing glucose concentration and the glucometer response, in the presence of TdT, the reaction of the primer to produce the extended probe induced aggregation of CeO_2_NPs by electrostatic attraction. This resulted in a significant reduction of the GOx mimicking activity of CeO_2_NPs hindering the oxidation of glucose whose concentration maintained at the initial level. Using this strategy, the TdT activity was reliably determined down to 0.7 U mL^−1^ with high selectivity against other non-specific enzymes.

#### 2.1.2. Multifunctional MNPs in Electrochemical Affinity Bioplatforms

Electrochemical immunodetection is currently a mature methodology that has led to relevant results in different analytical fields. During the last years, the use of metallic nanoparticles for the construction of immunosensors has been a common practice. The performance of some of them are summarized in Table 1. What can be said about the current state of this area is that increasing multifunctionality to nanomaterials is more and more in demand in order to minimize or even avoid the use of unstable, expensive, and difficult to manipulate biological materials. In this context, label-free configurations, where MNPs play different roles, are among the most reported in the literature. The ability of these nanoparticles and hybrids or combinations to act as artificial nanozymes has been discussed in the previous section. However, here, some examples of their use as modifiers of immunosensing platforms for creating nanostructured surfaces with low background current and fast electron transfer, as well as their applications as signal amplification labels, are discussed.

Highly sensitive detection of tumor biomarkers is a difficult task where electrochemical immunosensors have provided spectacular results. As a relevant example, in 2016, Chang et al. [11] reported a triple signal amplification strategy for the sandwich-type immunosensing detection of alphafetoprotein (AFP). The method involved electroactive polymer nanospheres synthesized from ferrocene dicarboxylic acid further decorated with PtNPs and DNAzyme (hemin/G-quadruplex) as highly conducting label tags. In the resulting conjugates, PtNPs acted as immobilizing supports for detection antibodies also reinforcing the peroxidase activity of DNAzyme to catalyze H_2_O_2_. Therefore, the sensitivity of this electrochemical immunosensor was greatly improved due to the combination and synergistic effect of the three involved elements. The linear range of the calibration plot extended from 0.1 pg mL^−1^ to 100 ng mL^−1^ and the LOD value was 0.086 pg mL^−1^.

Liu et al. [19] reported the excellent peroxidase-like activity and good stability of AuPd-polydopamine (PDA) conjugates in the preparation of a sandwich-type electrochemical immunosensor for the detection of the biomarker apolipoprotein E4 (APOE4) related to Alzheimer’s disease. Due to the good biocompatibility and large specific surface area, the synthesized AuPd-PDA nanotubes exhibited multifunctional properties as signaling molecules and carrier tags to load detection antibodies. Moreover, this approach also made use of gold nano-bipyramid coated Pt (AuBP@Pt) nanostructures as modifiers of the electrode surface for immobilizing specific anti-APOE4 capture antibodies. The resulting immunosensor exhibited a wide linear range between 0.05 and 2000 ng mL^−1^ and a low LOD value of 15.4 pg mL^−1^. Hartati and coworkers [15] developed also a label-free immunosensor for the detection of HER2 (human epidermal growth factor receptor 2) breast cancer biomarker by immobilization of the thiolated capture antibody onto CeO_2_ previously functionalized with 3-aminopropyl trimethoxisylane (APTMS) and polyethylene glycol-α-maleimide-ω-NHS (PEG-NHS-Mal). The resulting bioconjugate was immobilized on the surface of an AuNPs/SPCE. In the presence of HER2, the electron transfer of the redox probe Fe(CN)_6_^3−/4−^ was inhibited and the current decrease was proportional to HER2 concentration. The as-prepared immunosensor achieved a limit of detection of 34.9 pg mL^−1^. A different strategy for the detection of the same biomarker was reported, involving the immobilization of a tetrahedral DNA nanostructure–aptamer 1 onto a gold electrode and the implementation of a sandwich-type configuration by attachment to another aptamer, which recognized a different epitope of HER2, loaded together with HRP on a Mn_3_O_4_/Pd@Pt nanocarrier (nanoprobe 1). Further signal amplification was achieved by adding a DNA complementary to the sequence in nanoprobe 1 tagged with Pd@Pt/HRP. These components enhanced the electrochemical signal because they catalyzed the oxidation of hydroquinone (HQ) with H_2_O_2_ to benzoquinone (BQ), showing a wide linear range between 0.1 and 100.0 ng mL^−1^ and a low limit of detection of 0.08 ng mL^−1^ [29].

An effective signal amplification strategy was applied by Li et al. [17] in the construction of a label-free amperometric immunosensor for prostate specific antigen (PSA) involving AuNPs and Cu_2_O@CeO_2_ nanocomposites. AuNPs were bound to the amino-functionalized oxides to obtain an electrode platform with large specific surface area and good biocompatibility where the presence of MNPs not only enhanced the electron transfer but also increased the amount of immobilized capture antibody. Furthermore, the combination of two oxides provided a better electrocatalytic activity towards the reduction of H_2_O_2_ than that shown by a single oxide. A wide linear range, from 0.1 pg mL^−1^ to 100 ng mL^−1^ and a low limit of detection, 0.03 pg mL^−1^, were achieved. Following a similar scheme, the same authors prepared an immunosensor to detect squamous cell carcinoma antigen (SCCA) which involved amino-functionalized cobalt tetraoxide@ceric dioxide nanocubes to bind AuNPs and PtNPs giving rise to Co_3_O_4_@CeO_2_-Au@Pt nanocomposites used as labels of secondary antibodies for signal amplification. In addition, AuNPs were electrodeposited onto the surface of a GCE to prepare a platform for sensing and immobilization of capture antibodies. Due to the synergetic effect, excellent electrochemical behavior, and electrocatalytic activity of all components, a broad linear range from 100 fg mL^−1^ to 80 ng mL^−1^ with a low LOD of 33 fg mL^−1^ for detecting SCCA were obtained [18].

**Figure 2:**
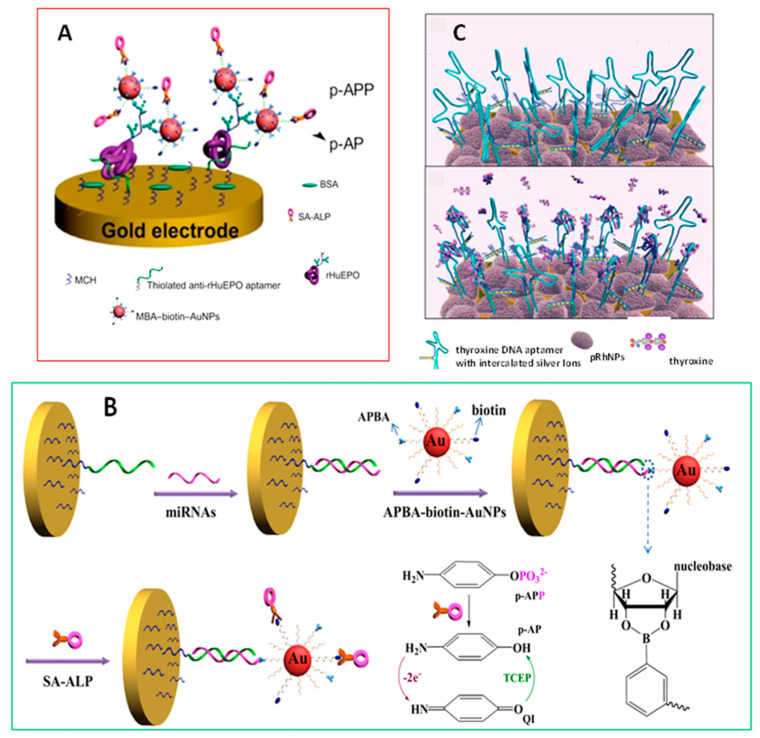
Schematic display of (**A**) the signal-amplified electrochemical biosensor for the detection of rHuEPO glycoprotein by using MBA-biotin-AuNPs as labels; (**B**) the label-free detection of miRNAs based on the triple signal amplification of APBA-biotin-AuNPs, SA-ALP, and the p-APredox-cycling reaction; and (**C**) the thyroxine biosensor in conditions of (up) absence and (down) presence of the analyte. Reproduced from [34] (**A**), [35] (**B**), and [38] (**C**) with permission.

The use of aptamers, small strands of DNA or RNA, in the design of electrochemical biosensors to specifically bind targets with a high affinity, provides several distinctive properties in comparison with antibodies such as high stability, small sizes, and ease of synthesis, making aptamers valuable molecular acceptors for the efficient and selective capture and recognition of biomolecules. Moreover, the combination of these bioreagents with multifunctional MNPs allows the enhancement of sensitivity for ultrasensitive detection of analytes. An interesting example of these improved features is that reported by Liu et al. [34] using 4-mercapto-phenylboronic acid/biotin-modified multifunctional AuNPs (MPBA-biotin-AuNPs) for the determination of recombinant human erythropoietin (rHuEPO), a glycoprotein hormone extensively used in the treatment of several anemias. The configuration involved immobilization of capture anti-rHuEPO onto the aptamer-coated electrode followed by MPBA-biotin-AuNPs attachment through the boronic acid–carbohydrate interaction (Figure 2A). The signal was monitored after the addition of streptavidin-conjugated alkaline phosphatase (ALP), promoting the production of electrochemically active *p*-aminophenol (*p*-AP) from *p*-aminophenyl phosphate (*p*-APP) substrate. The same authors developed a high-performance electrochemical biosensor for the detection of microRNAs (miRNAs) based on triple signal amplification of multifunctional AuNPs, ALP, and *p*-AP redox-cycling [35]. In this design, AuNPs were modified with CALNN and CALNNGK (biotin) G peptides and derivatized with 3-aminophenylboronic acid (APBA). The miRNAs were captured by the DNA probes on a gold electrode (Figure 2B). Amperometric label-free detection was made based on the difference in the structure of RNA versus DNA and the formation of covalent bonds between boronate moieties and cis-diols in nucleosides which avoided non-specific binding on DNA-covered surface. The measured current increased linearly with the miRNA-21 concentration between 10 fM and 5 pM, with a LOD value of 3 fM. In another strategy, the detection of miRNA was carried out after hybridization with a hairpin DNA probe previously immobilized onto the electrode surface and subsequent derivatization of the cis-diol moiety in the ribose of miRNA-21 with MPBA, which besides reacting with nucleotide to form the boronate ester, induced the aggregation of citrate-capped AgNPs through the Ag-S and citrate-boronate interactions acting as the cross-linker of AgNPs assembly. 

Other less frequently used MNPs such as those of rhodium have also found application in electrochemical biosensing. Porous rhodium nanoplates (pRhNPs) were used as immobilization support and modifier of a gold microgap electrode, providing an increase of the surface area and amplification of the electrochemical response. Multifunctional DNA-pRhNPs heterolayers have been prepared for the label-free detection of small molecules. As an example, Park et al. [37] developed an electrochemical label-free biosensor for the detection of thyroxine, the main thyroid hormone secreted by the thyroid gland. In this design (Figure 2C), a multi-functional DNA bioprobe was prepared using a thyroxine aptamer DNA to bind the target and four complementary C-C mismatched sequences with silver ions intercalated between them to report the electrochemical signal. Additionally, a thio-terminal group was used for immobilization to the Au electrode. Cyclic voltammetry (CV) and electrochemical impedance spectroscopy (EIS) were used to detect thyroxine based on the marked increase in the charge transfer resistance produced in the presence of the hormone over a concentration range from 100 nM to 1 pM with a limit of detection of 10.33 pM.

### 2.2. Multifunctional MNPs Involving Ordered Nanostructures

**Figure 3:**
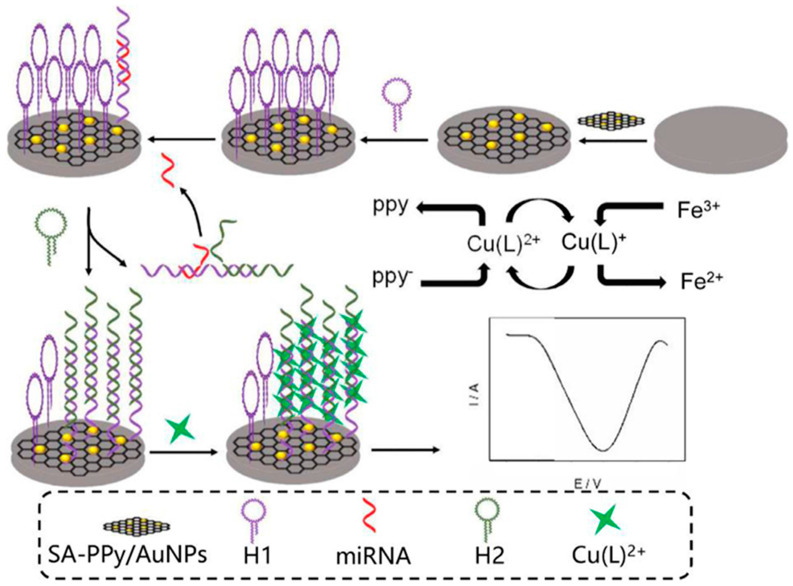
Scheme of the strategy for the electrochemical determination of miRNA-21 using a nanocomposite of sodium alginate-polypyrrole/Au nanoparticles (SA-PPy/AuNPs). Reproduced from [36] with permission.

The combination of MNPs with polymers [36,38], metal-organic frameworks (MOFs) [39,47,48,49], or dendrimers [23,32] provides nanocomposites with multiple functionalities in an ordered array of nanoparticles yielding a large number of active sites to allow highly sensitive detection. Table 1 summarizes some selected electrochemical biosensing applications involving such combination. For example, the incorporation of AuNPs to the network of polymers such as polypyrrole is a common strategy to increase their conductivity and compatibility with biomolecules. Tian et al. reported an electrochemical biosensor for the determination of miRNA-21 using a polypyrrole-AuNPs superlattice as electrode modifier and support material for the immobilization of ssRNA and toluidine blue (TB) as the hybridization indicator. This strategy led to a remarkable increase in the number of immobilized probe molecules on the electrode surface, allowing the determination of the target miRNA in a concentration range between 100 aM and 1 nM with a limit of detection of 78 aM [38]. Ma et al. synthesized a nanocomposite involving sodium alginate-polypyrrole/Au nanoparticles (SA-PPy/AuNPs) for the electrochemical detection of miRNA-21, taking advantage of signal amplification through catalytic hairpin assembly and the spontaneous catalytic reaction of Fe^3+^/Cu^2+^. The resulting configuration (Figure 3) displayed the multiple functionalities derived from the nanostructure composition including (1) the promotion of the reduction of Cu^2+^ that can be intercalated in the double helix of DNA, (2) the immobilization of the 3-terminal amino-modified hairpin capture probe through amidation reaction of previously activated carboxyl groups in SA, and (3) the enhancement of conductivity by the presence of AuNPs. This strategy involved opening the hairpin H1 in the presence of a target miRNA, followed by the replacement of the target in the presence of another longer complementary sequence H2, thus leaving miRNA available to start a new cycle. The double helix structures formed resulted in the immobilization of more copper (II) complex on the surface of the working electrode. In addition, the electrochemical signal of Cu^2+^ was enhanced in the presence of Fe^3+^ in the electrolyte since Fe^3+^ oxidized Cu^+^/Cu spontaneously and maintained a high concentration of Cu^2+^ on the electrode surface [36].

Metal-organic frameworks (MOFs) are formed by organic ligands and metal ions or clusters strongly coordinated with distinctive properties such as permanent porosity, abundant structures, and tailorable surface chemistry [69]. MOFs have been employed in (bio)sensing and, in particular, as the signal labels of electrochemical sensors. Noble metal NPs or alloy NPs can be adsorbed on the surface of MOFs for improving electrical conductivity among other properties. Chen et al. [47] prepared an ultrasensitive enzyme-free DNA sensor for the detection of the fibroblast growth factor receptor 3 (*FGFR3*) gene mutation based on the encapsulation of hemin into an amino-contained Fe-MIL-88 MOFs functionalized with PtNPs on which the capture probe was immobilized. The resulting hemin-MOFs/PtNPs were used for signal amplification because of the high conductivity and ability for H_2_O_2_ catalysis. Moreover, the electrode surface was modified with reduced graphene oxide–tetraethylenepentamine functionalized with AuNPs and streptavidin to further improve the amplification of the electrochemical response. The as-prepared DNA sensor was able to detect the synthetic target DNA in the 0.1 fM to 1 nM range with a LOD of 0.033 fM and was applied to early non-invasive prenatal diagnosis.

**Figure 4:**
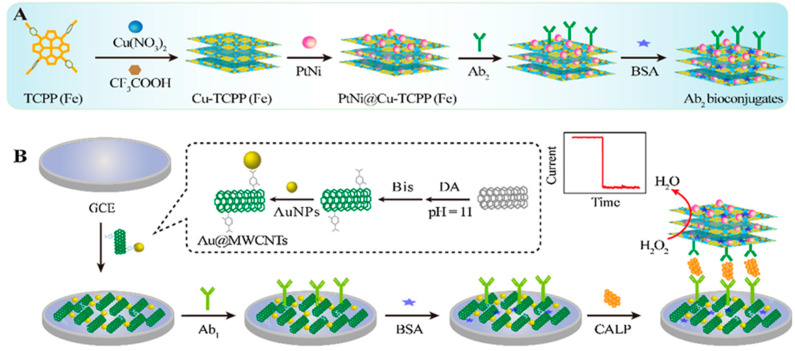
Schemes of (**A**) the preparation procedure of PtNi@Cu-TCPP(Fe)-Ab_2_ bioconjugates and (**B**) the construction process of the electrochemical immunosensor for calprotectin (CALP). Reproduced from [12] with permission.

Recently, ultra-thin MOFs layers with large surface area and excellent electrocatalytic activity, a high number of accessible sites for biomolecular adsorption and, most importantly, good dispersibility in solution, have been synthesized [70]. In this context, Li et al. reported the preparation of a Co-MOF nanosheet array on nickel foam (NF) which was used as the sensing material for the non-enzymatic electrochemical detection of glucose on the basis of the high electrocatalytic effect towards glucose oxidation. This Co-MOF/NF glucose sensor showed a fast response of 5 s and a wide linear range between 0.001 and 3 mM with a limit of detection of 1.3 nM [49]. MOF nanosheets have also been used as nanocarriers in the design of electrochemical immunosensors where their combination with MNPs provides additional enhanced catalytic activity, resulting in a larger signal amplification. In this sense, Dong et al. prepared a sandwich-type enzyme-free electrochemical immunosensor for calprotectin (CALP) by synthesizing MOF nanosheets using Fe (III) tetra(4-carboxy-phenyl)porphine chloride (TCPP (Fe)) as heme-like ligand and copper as metal nodes. Upon immobilization of PtNi nanospheres (Figure 4), the resulting PtNi@Cu-TCPP (Fe) hybrids provided specific surface area and active sites to bind detection antibodies. Furthermore, the electrochemical immunosensor was fabricated by capture antibody assembling onto a GCE modified with AuNPs-functionalized carbon nanotubes (Au@MWCNTs/GCE). The immunosensor allowed greatly amplified amperometric responses over a wide linear range of 200 fg mL^−1^ to 50 ng mL^−1^ [12].

**Figure 5:**
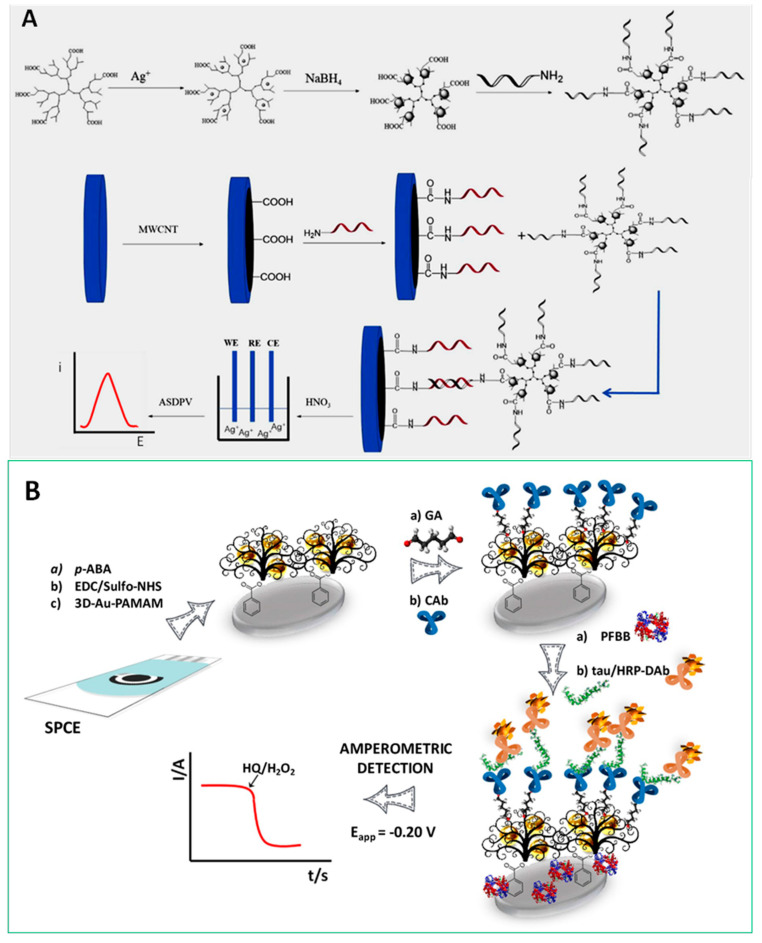
(**A**) Schematic display of the method for the detection of DNA hybridization using Ag-DNCs as label; (**B**) Fabrication and amperometric transduction involved in the development of an HRP-DAb-tau-CAb-3D-Au-PAMAM-*p*-ABA-SPCE immunosensor for the determination of tau protein. Reproduced from [32] (**A**) and [21] (**B**) with permission.

Dendrimers are monodisperse hyperbranched polymers with three-dimensional structures widely employed as “soft” nanomaterials in electrochemical biosensor technology [71]. Apart from their unique physicochemical properties such as globular or ellipsoidal shape with nanometric size, structural uniformity and monodispersity, the high density of surface functional groups and the permeability into internal cavities are important features in bioelectroanalytical applications. Moreover, dendrimers can be widely fine-tuned to promote electrocatalytic and charge-transfer processes on the electrode surface through proper encapsulation or covalent binding of MNPs and electron transfer mediators. A relevant example is the preparation of an immunosensor for the detection of α-fetoprotein (AFP) involving the immobilization of AuNP-dendrimer conjugates on the transducer surface, previously coated with chitosan using glutaraldehyde. The high density of chemical groups at the periphery of poly(amidoamine) (PAMAM) served as linking points for immobilization of ferrocene groups on the electrode surface, allowing sensitive label-free detection of this cancer biomarker through the decrease in the voltammetric response of ferrocenyl groups after the recognition event [23]. Jin et al. synthesized silver-dendrimer nanocomposites, which were used as oligonucleotide labels for the detection of DNA hybridization (Figure 5A). After the hybridization process, the silver nanoclusters were dissolved using strong acid and the released Ag^+^ ions were quantified by anodic stripping voltammetry [32]. Moreover, our group developed a strategy for the determination of tau protein, a hallmark of Alzheimer’s disease, using an AuNPs-PAMAM dendrimer nanocomposite covalently immobilized onto electro-grafted *p-*ABA. Figure 5B shows as the capture antibody was immobilized by cross-linking with glutaraldehyde on the amino groups of the modified electrode and tau protein was sandwiched with a detection antibody labeled with HRP. This method provided a LOD of 1.7 pg mL^−1^ [21] and was applied to the simultaneous determination of tau and TAR DNA-binding protein 43 (TDP-43) biomarkers of neurodegenerative disorders [22].

### 2.3. Multifunctional Nanomaterials Involving Quantum Dots (QDs)

As it is well known, QDs are nanocrystals with size between 2 and 10 nm in diameter consisting of semiconducting metal salts, such as PbX, CdX, ZnX (with X: S, Se, Te) and containing 100 to 100,000 atoms per particle. QDs are characterized by versatile features such as large surface area, ultrahigh porosity, tunable structure, and high electrochemical activity, related to their composition and dimensionality. QDs have been used in electrochemical biosensing due to the ability of these nanomaterials to easily conjugate with enzymes [50], antibodies [13,16,20,51], and DNA [28,52,53,54,72], which has greatly boosted their use as labels for signal amplification. Furthermore, the compositional nature of QDs makes it possible to get sharp and well-resolved stripping voltammetric signals due to the well-defined oxidation potentials of their metal components, which has led to their wide exploitation as nano-tracers for the development of ultrasensitive electrochemical affinity biosensors [72] and multiplexed detection [73]. Applications using QDs are summarized in Table 1.

**Figure 6:**
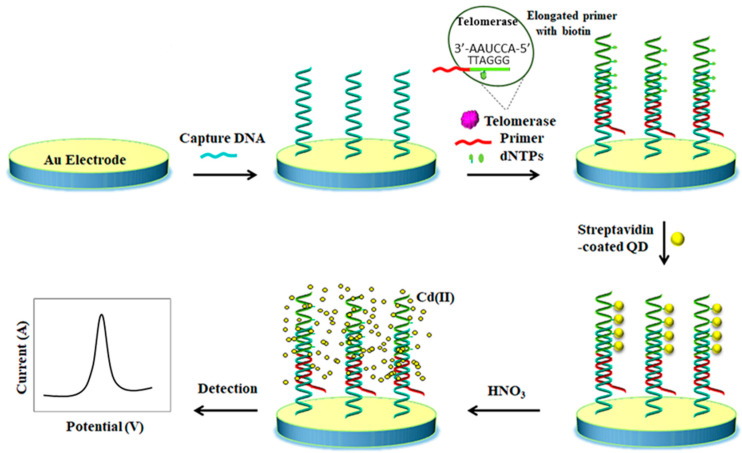
Schematic illustration of a QD-based electrochemical biosensor for the stripping voltammetric detection of telomerase activity. Reproduced and adapted from [52] with permission.

Li et al. prepared an electrochemical biosensor using streptavidin-Cd(II) QD bioconjugates for the detection of telomerase activity. A thiolated DNA capture probe was attached to a gold electrode surface whilst the telomerase extract was added to a reaction solution containing the specific telomerase primer and a biotin-dATP, dTTP, dGTP nucleotides mixture, thus taking place the addition of telomere repeats of (TTAGGG)_n_ to the 3′ end of the primer, getting multiple biotin anchoring points (Figure 6). The subsequent hybridization of extension product with the capture DNA and the addition of streptavidin-coated QDs induced the assembly of large amounts of strep-QDs onto the electrode. Later on, the dissolution of the anchored QDs in nitric acid solution released large amounts of Cd (II) which were quantified by square wave stripping voltammetry. The method allowed telomerase activity to be detected at the single cell level [52]. Multiplexed ASV-based bioassays using QD labels with different composition have been reported taking advantage of the separate oxidation potentials of metals in the stripping step. As an example, Rezaei et al. developed an electrochemical genosensor for the simultaneous detection of two hemophilia A related microRNAs. PbS and CdS QDs encapsulated ZIF-8 MOF particles were employed as signal-amplifying tags and catalytic hairpin assembly. The assay was conducted on an AuNPs modified GCE where each target can selectively open its harpin, leaving a fragment of DNA which hybridized with the PbS@ZIF-8-S1 or CdS@ZIF-8-S2. Finally, the encapsulated PbS and CdS QDs were dissolved and the released Pb(II) and Cd(II) quantified by DPV. Due to the encapsulation of a big number of QDs in each QDs@ZIF-8 label, the electrochemical signal was enormously amplified achieving LOD values of 0.19 and 0.28 fM for miR-1246 and miR-4521, respectively [54].

### 2.4. Two-Dimensional (2D) Transition Metal Multifunctional Nanomaterials

Transition metal oxides (TMOs) and chalcogenides (TCMs) prepared from elements of groups IV, V, and VI and S, Se, or Te, possess ultrathin thickness and planar structure as graphene-like materials, and exhibit certain unique chemical and physical properties due to the reduction in size and increase in the exposed active sites on their corner or edges. These nanomaterials have been used as transduction elements and supporting substrates in a wide variety of biosensing technologies due to its remarkable biocompatibility and large specific surface area which provides an extremely high density of active surface sites and fast electron transfer characteristics [73,74]. For example, molybdenum and tungsten disulfides are layered transition-metal dichalcogenides that can be isolated as monolayers or few-layer thick sheets because of the weak van der Waals interactions. These nanosheets can act as biosensing substrates usually combined with other nanomaterials such as gold nanoparticles [24,30,56,57], conducting polymers [40] or metal oxides [57], improving the resulting electrochemical biosensing performance (Table 1).

A representative example is the preparation of hollow MoS_2_ microcubes for the determination of microRNA-21 coupled with a duplex-specific nuclease, enzyme signal amplification, and electrochemical-chemical-chemical (EEC) redox cycling. In this strategy, the capture DNA probes labeled with biotin were immobilized onto MoS_2_ coupled to AuNPs and further hybridized with the target miRNA. The formed duplexes were cleaved by a duplex-specific nuclease. Thereafter, miRNAs were recycled and biotin groups at the end of the capture probe were also released from the electrode. The application of enzyme and EEC redox cycling greatly amplified the detection signal, achieving a LOD value of 0.086 fM [55]. Su and coworkers designed a MoS_2_-based electrochemical immunosensing strategy for the determination of carcinoembryonic antigen (CEA) using MoS_2_ nanosheets decorated with AuNPs. The MoS_2_-AuNPs nanocomposite was used as modifier material for the effective capture-antibody immobilization on the electrode surface and also as nanocarrier due to its large surface area, thus allowing large loadings of anti-CEA and HRP-anti-CEA. Moreover, the nanocomposite displayed enzyme mimicked activity and excellent conductivity. As a result, a wide linear range from 10 fg mL^−1^ to 1 ng mL^−1^ and an excellent limit of detection of 1.2 fg mL^−1^ were achieved [24]. A 2D-WS_2_ based nanocomposite with multifunctional properties was synthesized and employed for the construction of an electrochemical platform for the determination of carbohydrate antigen (CA72-4). In this method, WS_2_ nanosheets were functionalized with ferrocene monocarboxylic acid (FMC) and AuNPs. These nanoparticles increased the electrode active surface area and also acted as a mediator for electron tunneling, thus accelerating the electron transfer of redox FMC. The combination of the multifunctional nanocomposite with magnetic beads technology allowed the development of a high-performance electrochemical immunosensor [56].

More recently, a new group of 2D transition metal carbides/carbonitrides named Mxenes have attracted significant interest due to their unique properties such as metallic conductivity and hydrophilic surfaces [58,75]. Mxenes are synthesized by the selective etching of the “A” layers from the layered hexagonal M_n+1_AX_n_ phases (where M is an early transition metal such as Ti, V, or Nb, among others, A is usually an element from the group 13 or 14, X is carbon or nitrogen, and n = 1–3) [76]. Similar to other nanomaterials, Mxenes in combination with other components such as enzymes, MNPs, conducting polymers, and metal oxides, have served to prepare multifunctional nanostructures utilized in the development of electrochemical (bio)sensors. For example, the hybridization of Ag and AuNPs from solution onto exfoliated two-dimensional (2D) Ti_3_C_2_ titanium carbide nanosheets efficiently improved the chemical properties of MNPs due to lower aggregation and enhancement of electron transfer. Using this behavior, 2D layered materials nanohybrids obtained from MoS_2_ nanosheets and Ti_3_C_2_ were prepared for the ultrasensitive detection of miRNA-182 [59]. MoS_2_-Ti_3_C_2_ in combination with AuNPs were employed for the efficient immobilization of thiolated ssRNA, and [Fe(CN)_6_]^3−/4−^ as the redox probe. Because ssRNA is negatively charged through its phosphate backbone, a weak electrochemical signal was obtained in the absence of the target, due to electrostatic repulsion with the probe. Upon addition of miRNA-182, the grafting density onto the electrode surface increased and swelling-induced Au–S bond breakage occurred, releasing the dsRNA from the electrode surface. This biosensing platform provided a linear concentration range between 1 fM and 0.1 nM with a LOD of 0.43 fM.

## 3. Multifunctional Carbon Nanomaterials

Carbon nanomaterials have generated considerable interests in the past decades. Their tunable physical and chemical properties have led to a variety of applications in diverse scientific and technological fields. Combination of particular features of carbon nanomaterials such as electrochemical performance, high surface area, biocompatibility, enzyme-like behavior, adsorption ability, or electrocatalytic activity, allow them to be considered multifunctional materials able to perform various actions when used in the proper design. In addition, these nanomaterials can be easily modified by incorporating redox probes, biomolecules, magnetic labels or polymers, and can also be utilized in diverse nanoforms, geometric structures or chemical states that enhance their multifunctionality or improve the existing one.

In this section, recent examples of carbon nanomaterials that have proven their multifunctionality in various applications when used as components of electrochemical biosensors have been selected and their capabilities critically discussed.

### 3.1. Magnetic Carbon Nanomaterials

Magnetic iron oxides/carbon nanomaterials have attracted particular attention owing to their efficient, green, and economical multiple functionalities related to adsorption ability, outstanding catalytic activity, and (bio)sensing capacity. Among these materials, carbon nanotubes-based magnetic composites prepared by coating the outer wall of the nanotubes with magnetic nanoparticles by adsorption, covalent attachment, π-π stacking, or coating with polymeric films containing the iron oxide nanoparticles have found numerous applications [77]. A variety of electrochemical (bio)sensors have been reported in the literature where the presence of magnetic particles not only facilitates the preparation of the sensor but also provides other functionalities related to higher sensitivity, through enhanced electrical conductivity, and/or better selectivity derived from electrocatalytic effects toward redox processes involved in the detection scheme. Table 2 summarizes the main characteristics of recently reported electrochemical biosensing strategies involving magnetic carbon nanomaterials [78,79,80,81,82,83,84,85,86,87,88,89,90,91,92,93,94].

As an example, Khoshsafar et al. [78] prepared an electrochemical sensor using magnetic carbon nanotubes decorated with a molecularly imprinted polymer (m-CNTs@MIP) which was applied to the determination of levofloxacin (Lv). The resulting composite could be manipulated rapidly by an external magnetic field and had a high specific surface area as well as specific recognition capacity for Lv. The usual procedure with methacrylic acid as the functional monomer and ethylene glycol dimethacrylate as the crosslinker was employed to obtain MCNTs@MIP using Lv as the template. The resulting sensor was not significantly affected by the most common interfering species and provided a linear response over the 0.003–0.440 μM range with a LOD of 0.8 nM. The MIP sensor was applied to the analysis of spiked serum and urine. An amperometric immunosensor involving SPCEs grafted with *p*-aminobenzoic acid for covalent binding of streptavidin conjugated with the biotinylated capture antibody, and magnetic MWCNTs as nanocarrier tags for secondary antibodies was reported for the determinatio of fetuin (HFA), a relevant biomarker of inflammatory processes [79]. This approach provided analytical advantages in terms of wider concentration range and better sensitivity with respect to commercial ELISA kits. The linear calibration plot extended between 20 and 2000 pg mL^−1^ with a LOD value of 16 pg mL^−1^. In this immunosensor, the multifunctional detection label consisting of magnetic MWCNTs conjugated with HRP and detector antibody (anti-HFA) exhibited a large specific surface area with a high adsorption ability and promoted electron transfer with enhanced electrochemical responses for benzoquinone reduction at −200 mV. The improved analytical performance was partly attributable to the presence of magnetic particles on the external surface of MWCNTs and their pseudo-peroxidase activity. However, this enzyme activity was weak compared to that of HRP immobilized on the surface. The immunosensor was used for the determination of HFA in saliva with minimal sample treatment.

**Figure 7:**
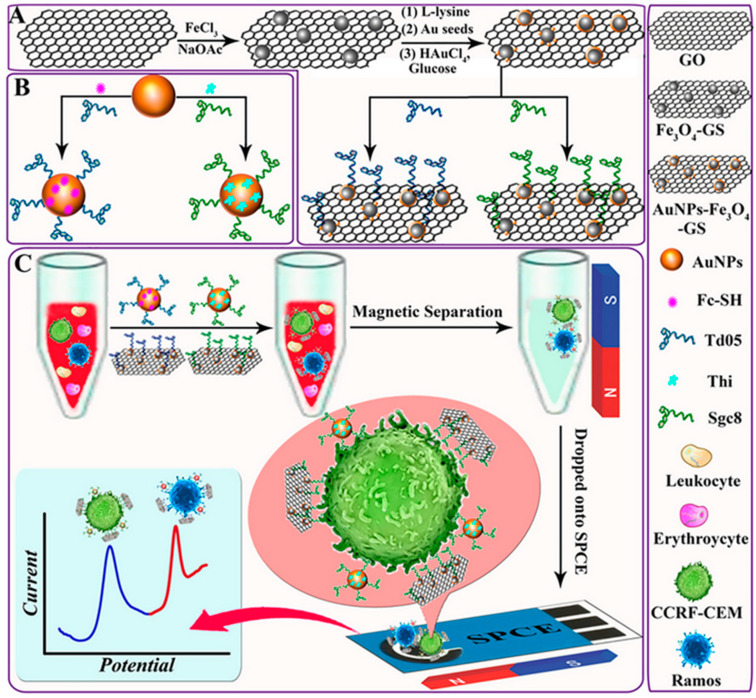
Preparation of (**A**) the aptamer-functionalized AuNPs-Fe_3_O_4_-GS capture probes and (**B**) the aptamer/redox probes functionalized AuNPs. (**C**) Steps of capture, isolation, and multiplexed detection of target CTCs. Reproduced from [80] with permission.

Detection and monitoring of circulating tumour cells (CTCs) in human blood is a major challenge in the diagnosis of different cancers because of their extremely low concentration levels and, therefore, the high sensitivity required. Dou et al. [80] prepared magnetic graphene decorated with gold nanoparticles (AuNPs-Fe_3_O_4_-GS) supported onto SPCEs as the electrode platform to capture and isolate CTCs from human whole blood allowing the separation and multiplexed electrochemical detection of different types of cells (Figure 7). Incubation of the probes with the sample solutions containing distinct CTCs (Ramos and CCRF-CEM) resulted in their efficient separation and the generation of two voltammetric peaks at specific potential values. The use of magnetic graphene sheets allowed the preparation of the electrochemical scaffold with various advantages such as the high amount of AuNPs dispersed on this surface which consequently led to the high loading of immobilized redox labels (Thi and Fc-SH) and specific aptamers (Sgc8 and Td05). Obviously, the magnetic properties of the resulting AuNPs-Fe_3_O_4_-GS facilitated the selective capture by the immobilized bioreagents and efficient isolation of CTCs from whole blood. Square-wave voltammetry (SWV) of Fc and Thi as the redox probes was utilized for the detection of Ramos and CCRF-CEM cells, respectively. Linear calibration plots from 5 to 500 cells mL^−1^ were obtained with LOD values of 3 and 4 cells mL^−1^, respectively.

### 3.2. Carbon Nanozymes

In recent years, several functional nanomaterials including metal oxides and nanocarbon-based materials which mimic functions of naturally occurring enzymes have been developed [124]. This class of artificial enzymes, named nanozymes [125], is an active research area in the field of biosensors due to the unique properties of nanozymes compared to natural enzymes in terms of stability, size-dependent catalytic activity, and easy bioconjugation. The use of nanozyme-based electrochemical biosensors for the detection of disease biomarkers has been reviewed recently [126,127].

Carbon nanomaterials have shown to possess enzymatic activity, which is a valuable functionality added to other properties of these materials. Carbon nanodots (CDs) and graphene quantum dots (GQDs), single- (SWCNTs) and multiwalled (MWCNTs) carbon nanotubes, carbon nanohorns (CNHs), and C60 fullerenes are some carbon nanomaterials exhibiting peroxidase activity [128]. Table 2 summarizes some applications involving carbon-based nanozymes [95,96,97,98,99,100,101,102,103,104,105]. This ability, together with the capacity of these nanomaterials for biomolecules immobilization, the improvement of electron transfer, the large surface area provided, and their rich surface chemistry, is known to benefit the construction of electrochemical biosensors with exciting multifunctionality. As it is known, horseradish peroxidase (HRP) is commonly employed in biosensing. However, the use of this enzyme requires labeling to a secondary receptor for target determination, thus making the assay procedures costly and time-consuming. Profiting the peroxidase-like activity exhibited by CNT-based materials, Cui et al. [129] synthesized helical CNTs with a large specific surface area and high catalytic activity for H_2_O_2_ and TMB and were used for the preparation of an amperometric sensor for H_2_O_2_ with a linear range between 0.5 and 115 μM.

Despite the nice behavior of CNTs alone, their combinations with other materials to form hybrids or composites that enhance peroxidase-like activity have been mostly used for the preparation of electrochemical (bio)sensors. Metal oxide [95,130] or metallic [96,97] nanoparticles, and other carbon nanomaterials [98,99] have been successfully employed for this purpose. A representative example is the preparation of Fe_3_O_4_/CNTs nanohybrids to enhance the peroxidase-like activity of nanotubes and also to take advantage of the large CNTs surface area for efficiently disperse Fe_3_O_4_ nanoparticles, thus preventing their aggregation [130]. In addition, the resulting hybrids also exhibit magnetism, which is very suitable for facilitating conjugation and sequential steps for the preparation of biosensors. Besides iron oxides, spinel-type chromites have shown suitable electrocatalytic properties derived from the large surface-to-volume ratio and high surface activity. CNTs have been used as support of these materials to promote the efficient nanozyme behavior towards the reduction of H_2_O_2_. So, a ZnCr_2_O_4_/MWCNTs spinel composite was employed for the enzyme-free detection of H_2_O_2_ in environmental and biological samples [95].

In another strategy, avidin-functionalized MWCNTs were decorated with ruthenium nanoparticles to prepare a multifunctional electrochemical interface on glassy carbon electrodes. The peroxidase-like properties of Ru [131] produced a synergic effect when combined with CNTs on the non-enzymatic catalytic reduction of H_2_O_2_ over a wide linear range from 0.5 μM to 1.75 mM. The resulting nanomaterial allowed the anchoring of biotinylated glucose oxidase for the preparation of a glucose biosensor which was applied to the analysis of commercial beverages [96]. MWCNTs have also been used as suitable supports for platinum nanoparticles stabilized onto poly(PAMAM) dendrimers (DEN) which avoided the aggregation and optimized both biocompatibility and catalytic activity towards H_2_O_2_ reduction. The resulting PtNPs/DEN/MWCNTs provided a non-enzymatic real-time determination of H_2_O_2_ released from breast cancer cells (MCF-7) using ascorbic acid as the stimulant agent [97].

Due to the peroxidase-like activities of CDs and GQDs [132], these carbon nanozymes have been used alone or combined with other materials for the preparation of electrochemical (bio)sensors, allowing the label-free detection of analytes by replacing HRP-based systems. As an example, Zhang et al. [104] assembled GQDs onto a gold electrode where their enzyme-like properties were exploited for the detection of H_2_O_2_ in living cells. Similarly, an electrochemical immunosensor using GQDs as enzyme mimics was developed to provide an efficient diagnostic method for *Yersinia enterecolitica*, a gram-negative bacillus shaped bacterium, which may contaminate eating food or water from infected human or animal feces. In this application, the multifunctionality of GQDs lies in their dual role of immobilizing specific antibodies and enzymatic activity. The GQD-modified gold electrode significantly enhanced electrocatalytic activity towards H_2_O_2_ reduction, probably due to the intimate electronic interactions between Au and GQDs, which improved the electron transfer. The detection of bacteria was based on the degree of inhibited electron transfer on the modified electrode due to the formation of the antigen–antibody complex. Therefore, the signal response of the immunosensor decreased along with the increased *Y. enterecolitica* concentration in the sample solution. A successful application was implemented for the analysis of milk and human serum, obtaining LOD values of 5 and 30 colony-forming unit (cfu) mL^−1^, respectively [101]. Hybrid nanocarriers involving GQDs and MWCNTs have been prepared to take advantage of their multifunctionality for signal amplification, immobilization of detection antibodies and peroxidase-like activity. As an example, a dual electrochemical immunosensor for the simultaneous determination of IL-13Rα2 and CDH-17, two biomarkers of emerging relevance in metastatic processes, was reported where the GQDs/MWCNTs-based hybrids were sandwiched with the antigens conjugated to capture antibodies immobilized onto *p*-aminobenzoic acid grafted screen-printed dual carbon electrodes [122].

Compared with their single component, all these hybrid materials exhibited enhanced peroxidase-like activities, most likely resulting from the synergetic effects of combined nanomaterials. Recently, it has been shown that Fe_3_O_4_ nanoparticles loaded as the third component on graphene oxide-dispersed CNTs have stronger enzyme-like activity. Amphiphilic GO nanosheets were employed as “surfactant” to disperse CNTs providing stable GO-dispersed CNT nanosupports in water for covalently loading cubic Fe_3_O_4_ nanoparticles [98].

The peroxidase-like activity of carbon nanozymes can also be increased via rational design of nanostructured materials as multifunctional nanozymes. Among other alternatives, doping heteroatoms into the π-conjugated system of carbon nanomaterials such as GQDs allows structure defects to be modified by providing unexpected properties [103]. This strategy constitutes an effective way to increase both pseudo-enzymatic behavior and specificity. As an example, N-doping enhances the peroxidase mimicking activities of carbon nanomaterials. In N-doped GQDs, the chemically bonded nitrogen atoms enhance the oxygen-containing functional groups, favoring water solubility and also increasing the number of anchoring sites to form stable chemical bonds with a variety of materials [133]. It is worth mentioning that up to a 100-fold increase in catalytic activity has been reported for the nitrogen-doped (N-doped) reduced graphene oxide (N-rGO) nanozymes compared to the reduced graphene oxide (rGO) alone. It was also shown that multiple enzyme-mimicking activities of carbon nanozymes were enhanced by nitrogen doping [134]. Although carbon nanozymes have demonstrated good catalytic efficiency, especially when combined with other nanomaterials, several challenges are still present and future efforts must focus on achieving greater enzymatic activity by promoting the use of new materials and the rational design of these and their combinations.

### 3.3. Multifunctional Biomedical Applications

Carbon nanomaterials are well suited for many relevant biomedical applications (Table 2) [106,107,108,109,110,111,112,113,114]. As it is known, theranostics is an emerging area that can take advantage of the use of nanomaterials, for example, as nanocarriers acting to detect agents and deliver multiple components, thus facilitating simultaneous synergistic diagnosis and therapies. In this field, graphene derivatives have unlocked a new era of biomedical nanomaterials due to their biocompatibility, physicochemical, and mechanical properties. Graphene nanomaterials combined with different inorganic and organic materials find wide applications in diagnostic, biosensing therapeutics, and drug delivery. Thus, graphene-polymeric nanocomposites have been used for drug delivery with the flexibility to incorporate hydrophilic or hydrophobic species and macromolecules. On the other hand, the special properties of CNTs facilitate their multifunctional applications in biomedicine both in diagnostic and therapeutic [135]. Indeed, their high biocompatibility and functionalization capacity make them excellent candidates as drug vehicles [136]. Oxidized CNTs are commonly used for these applications due to the lower amount of impurities and the existence of surface carboxylic gatherings that allow specific functionalization with targeting moieties. The use of CNTs as platforms in therapeutic agent delivery systems ranging from anti-infectious, anti-neoplastic agents, cardiovascular drugs to genes, and anti-inflammatory molecules, has been recently reviewed [137]. In this context, the inherent properties of CNTs, such as large surface area, high loading capacity, chemical stability, and great mechanical strength, make them excellent nanocarriers through functionalization. An interesting application in this area is the fabrication of stimuli-responsive film electrodes and logic gates. Zhou et al. [138] prepared a polymeric/GO electrode modified with SWCNTs to detect switching behaviors with temperature changing using hydroquinone and catechol as the target compounds. Carboxylated-MWCNTs and poly(*N*,*N*-diethylacrylamide) were also used for the preparation of hydrogel surface layers onto GCEs to study the electrochemical on-off behavior toward the drug molecules matrine and sophoridine [139]. Similar components were employed by the same authors to develop a multiple stimuli-responsive hydrogel sensor and a logic-gate system for rutin, which might be applied to the intelligent medical diagnostics and drug release [140].

Materials able to glucose sensing and allow the treatment of diabetic patients through insulin delivery are of high interest. Belkhalfa et al. [106] prepared multifunctional rGO/insulin/Ni(OH)_2_ films which exhibited excellent electrocatalytic behavior with high sensitivity towards the oxidation of glucose in alkaline medium, and allowing the electrochemical triggered release of insulin integrated into the film upon application of a negative potential to the interface.

The construction of theranostic systems that combine cancer treatment and diagnosis agents on a single platform has received great attention in the field of nanobiomedicine. Graphene derivatives have been used for this purpose. An illustrative example is a multifunctional system involving reduced graphene oxide and polydopamine further modified with BSA and decorated with a manganese (II) chelate, acting as a diagnostic platform using methotrexate anticancer drug as the target compound. The capturing ability of the developed surface was successfully evaluated through EIS by using 4T1 cancer cells, and the specificity was tested towards L929 normal cells. Furthermore, in vitro methotrexate release experiments were performed with results showing that about 80% of the drug was released from the system during the first 12 h of incubation at pH 7.4 [141]. The same team reported a similar theranostic system involving folic acid and 5-fluorouracil anticancer drug to target CT-26 colon cancer cells via overexpressed folate receptors. Voltammetry was utilized to investigate the system configuration [142].

**Figure 8:**
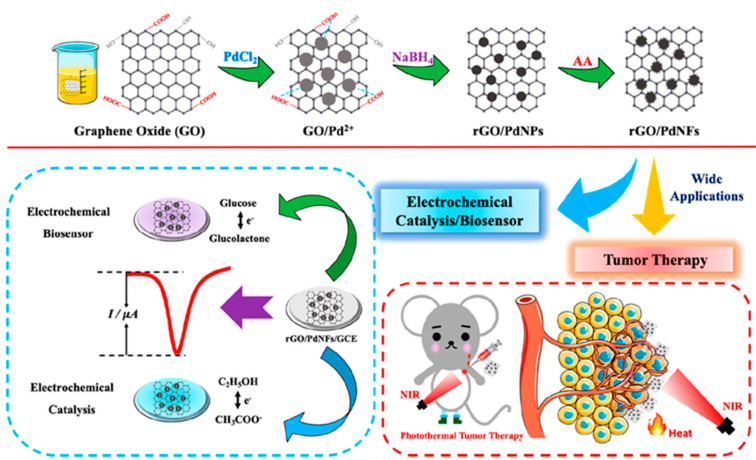
Schematic illustration of the synthetic route of rGO/PdNFs nanohybrids (top) and applications in non-enzymatic glucose sensing, electrochemical catalysis of ethanol, and photothermal tumor therapy in vivo (bottom). Reproduced from [107] with permission.

An excellent example of multifunctionality is the nanocarbon hybrid prepared by He et al. [107] consisting of reduced graphene oxide modified with palladium nanoflowers (rGO/PdNFs), which possesses various properties including non-enzymatic activity, electrocatalysis, and tumor therapy. As illustrated in Figure 8, the synthesized nanohybrid could be utilized as GCE modifier in an electrochemical sensor for glucose, providing a LOD value of 82.2 nM or applied in the electrochemical catalysis of ethanol, and photothermal tumor therapy.

### 3.4. Multifunctional Carbon Nanomaterials for Signal Amplification

Among the multiple functions of carbon nanomaterials, amplification of the electrochemical signals provided by (bio)electrochemical devices has found extensive applications (see Table 2) [115,116,117,118,119,120,121,122,123]. To accomplish signal amplification two main ways, involving different mechanistic strategies, can be considered: (a) increasing the loading of electrochemically detectable species or the redox mediators or the catalysts, and (b) enhancing the electrochemical response as the result of an improved electron transfer. Carbon nanomaterials can be deposited onto electrode surfaces or used as labels or carrier tags to increase the loading of the species of interest. In most cases, the carbon nanostructures used to amplify electrochemical responses act as effective supports to immobilize bioreagents, whilst providing high magnitude currents, exhibit electrocatalytic effects, increasing the selectivity of the detection, and sometimes act as nanozymes simplifying biosensor configurations.

Multifunctional CNTs were used by Sánchez-Tirado et al. [115] in the preparation of an electrochemical immunosensor for the determination of transforming growth factor β1 (TGF-β1) cytokine. Viologen-SWCNT hybrids synthesized by aryl-diazonium chemistry and conjugated with HRP and anti-TFG antibodies were used as label tags for signal amplification. Viologens are 4,4′-bipyridine derivatives which exhibit reversible electrochemical responses at negative potentials. Such reversible redox behavior promotes the electron exchange between electrodes and proteins with the subsequent decrease in the ohmic overpotential [143]. The resulting immunosensor provided a linear range extending between 2.5 and 1000 pg mL^−1^ and a LOD value of 0.95 pg mL^−1^ TGF-β1. An aptasensor for the determination of tetracycline was constructed using carboxyl-functionalized MWCNTs decorated with AuNPs as the carrier for the complementary strands and Thi as the electroactive probe. The specific aptamer was immobilized onto a gold electrode and the change in the differential pulse voltammograms of Thi was related to the antibiotic concentration though a linear range between 0.1 nM to 1 mM with a LOD value of 0.06 nM [116].

**Figure 9:**
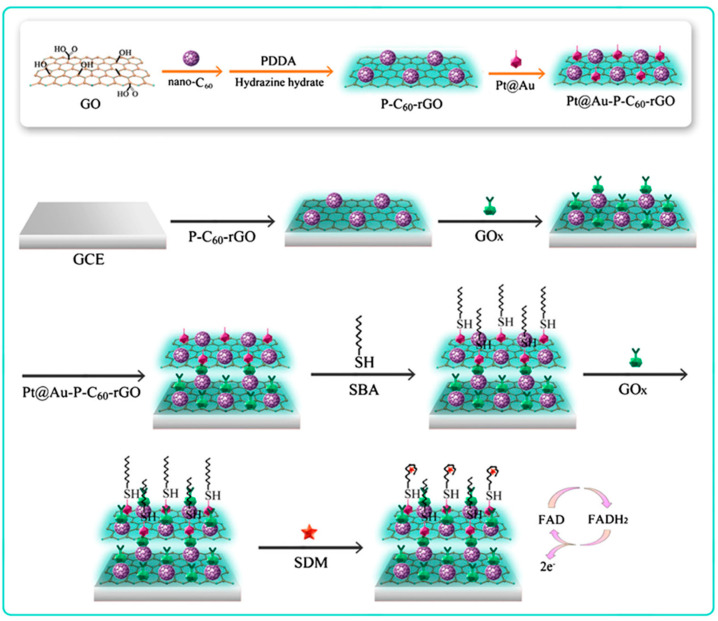
Schematic illustration of the stepwise construction of an aptasensor for the determination of sulfadimethoxine (SDM) using fullerene-doped reduced graphene oxide nanohybrids. Reproduced from [118] with permission.

Nanocomposites involving various carbon nanomaterials have attracted much attention for the fabrication of multifunctional tools used in response amplification schemes. For instance, carbon-based nanocarriers with multifunctional activity involving SWCNTs and peroxidase-like GQDs composites were used for enzyme-free electrochemical determination of carcinoembryonic antigen (CEA) [117]. A sandwich-type immunoassay was implemented between detector antibodies immobilized onto SWCNTs@GQDs and the immunocomplex formed by the antigen and the capture antibody on the rGO/AuNPs-modified GCE. The dual signal amplification made possible the detection of CEA from 50 to 650 pg mL^−1^ with a LOD value of 5.3 pg mL^−1^. Fullerene-doped reduced graphene oxide (C_60_-rGO) nanohybrids exhibit large specific surface area, excellent conductivity, and unique adsorption ability. This material was utilized for signal amplification in the design of an electrochemical aptasensor for the determination of sulfadimethoxine (SDM). The method involved a GCE modified with C_60_-rGO coated with poly(diallyldimethylammonium) and GOx (GOx/PDDA/C_60_-rGO/GCE). As Figure 9 shows, Pt@Au NPs were also added for further immobilization of thiolated SMD-binding aptamer. In the presence of SMD, the voltammetric responses generated by the direct electrochemical transfer between the redox center of GOx (FAD/FADH_2_) and the electrode surface were related to the concentration of the analyte over the 10 fg mL^−1^ to 50 ng mL^−1^ range with a LOD value of 8.7 fg mL^−1^. The high sensitivity was attributed to the increase in the electrode surface area provided by PDDA/C_60_-rGO, which enhanced the adsorbed amount of GOx onto the electrode, as well as to the presence of Pt@Au NPs that enhanced the immobilization of the thiolated aptamer and the amplification of the GOx electrochemical responses [118].

A very interesting material in this context is graphitic carbon nitride (g-C_3_N_4_), a non-metal semiconductor with adjustable electronic structure and high specific surface area. The g-C_3_N_4_ planes are composed of tris-triazine (C_6_N_7_) units connected by a planar tertiary amine group, so it can easily capture the target molecules [144]. However, due to the relatively low conductivity of g-C_3_N_4_, it is usually combined with other materials such as metals or CNTs for improving the electrochemical performance. As a representative example, a g-C_3_N_4_-CNTs composite with surface self-assembled PdNPs was prepared as a catalytic amplification platform for the voltammetric determination of 17α-ethinylestradiol (EE2) in feedstuffs [119]. Mesoporous g-C_3_N_4_ (mpg-C_3_N_4_) providing fast electron transfer and good biocompatibility has also been employed as the sensor platform and the carrier tag for constructing an electrochemical immunosensor for the detection of subgroup J of avian leukosis viruses (ALVs-J). Primary antibodies were immobilized onto the mpg-C_3_N_4_ modified GCE and a composite of mpg-C_3_N_4_ and electroactive Thi served as the carrier of secondary antibodies and the redox probe, respectively. The resulting immunosensor provided a low LOD of 120 TCID50/mL (TCID50: 50% tissue culture infective dose).

**Figure 10:**
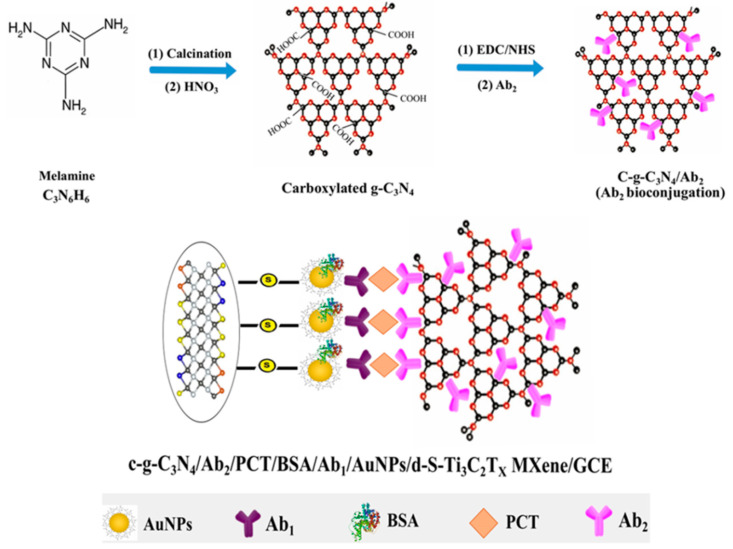
Preparation of carboxylated c-g-C_3_N_4_ and Ab_2_ bioconjugate (**up**) and sandwich-type immunosensor (**down**). Reproduced from [121] with permission.

The application of 2D transition metal carbides and nitrides (MXenes) [120] should also be commented on. In a recent example, a sensitive sandwich-type electrochemical immunosensor was reported for the determination of procalcitonin, a peptide used as a biomarker for septicemia. The immunosensor design involved the use of carboxylated-graphitic carbon nitride (c-g-C_3_N_4_) as carrier tag of detector antibodies (Ab_2_) for signal amplification (Figure 10). Interestingly, c-g-C_3_N_4_ not only exhibited an excellent catalytic activity toward H_2_O_2_ but could also be used directly as a redox probe. Moreover, a sulfur-doped MXene (d-S-Ti3C2TX MXene)-modified GCE including AuNPs was employed as immunosensor platform to increase the amount of immobilized capture antibody, which resulted in an improved sensitivity yielding a linear range extending between 0.01 and 1.0 pg mL^−1^ and a LOD value of 2.0 fg mL^−1^ [101].

## 4. Multifunctional Silica Nanomaterials

Mesoporous silica nanomaterials are defined by IUPAC as materials with a pore size of 2–50 nm functionalized with various supplementary groups [145,146]. These materials have attracted considerable attention in electrochemical biosensing due to their privileged characteristics, such as functionalizable and large specific surface area, mechanical stability, biocompatibility, affordable cost, and three-dimensional structure made of highly open interconnected spaces [147,148,149].

Table 3 summarizes the main types of mesoporous silica nanomaterials described so far and their characteristic features. Among them, mesoporous silica nanoparticles (MSNs) have aroused great interest in electrochemical biosensing because of their dual function of porous materials and nanomaterials and outstanding physiochemical features such as large surface area, high pore volume, uniform size and large inner space, controlled pore structure, fine suspendability in aqueous solution, and easiness to be functionalized by encapsulation or immobilization of a large amount of payloads (mediators, enzymes, antibodies) [149,150,151,152,153]. Moreover, due to their silicate inorganic framework, these materials are thermal, chemically, and mechanically stable, relatively environmentally inert and resistant to microbial attacks [151,154]. Their structure allows good homogeneity, high integrity, controlled composition, and good biocompatibility, with a demonstrated improvement of enzymes stability [149]. In addition, their unique topology allows three distinct domains to be independently and straightforwardly functionalized: the silica framework, the uniform nanochannels/pores, and the nanoparticle’s outermost surface [148,155,156,157,158]. MSNs, with versatile pore structure and functionality, can be fabricated with tunable size, shape, and pore diameter using simple and affordable procedures [159]. MSNs can be modified efficiently and stably with a huge number of macromolecules like DNA, RNA, and proteins since they have silanol groups with metal ions and inorganic frameworks [160,161].

Similar to other nanomaterials, MSNs can be combined with other nanomaterials such as CNTs [165,178], graphene oxide (GO) [179], GQDs [175], inorganic QDs [180], AuNPs [151,152,154,181], AgNPs [181], Ag NCs [182]), Fe_3_O_4_ NPs [181,183,184] to impart the nanohybrids as well as the resulting electrochemical biosensing devices with improved performance due to the combined or synergic properties in terms of easy magnetic manipulation and improved conductivity and loading capacity.

For example, the low charge transfer efficiency of MSNs has been surpassed by combining them with AuNPs and CNTs, while preventing the NPs aggregation [182]. On the other hand, the easy aggregation and limited functional groups of Fe_3_O_4_ NPs, which make their application in electrochemical biosensors challenging, can be solved by preparing core-shell Fe_3_O_4_@SiO_2_ NPs [183]. The SiO_2_ shell enhances the storage stability, improves the NPs dispersibility and provides a large number of –OH surface groups for further modification. Moreover, the formation of a SiO_2_ shell on the surface of CNTs effectively enhances the solubility of CNTs to a certain extent and also provides a high surface area with a porous structure, leading to a suitable bionanomaterial for constructing high-performance electrochemical biosensors [178].

It is also remarkable the functionalization of MSNs with ionic liquids (ILs), such as 1,4-diazabicyclo[2.2.2]octane (DABCO) [169,181], to produce an ionic liquid framework on their surface helpful for the deposition of Au NPs preventing their aggregation and formation of dendritic Au nanostructure due to the N groups.

MSF is an amorphous, stiff mesoporous silicate material with high mechanical stability, formed by hexagonal arrays of one-dimensional, parallel cylindrical channels. MSF appears as an appealing nanosupport to design stable electrochemical biosensors due to its large surface area-to-volume ratio, well-ordered pore structure, uniform pore size, and easy modification due to the large content of hydroxyl groups at the surface [176]. Growing of MSFs on the electrode surface offers an inimitable array of perpendicular nanochannels with excellent permeability properties and unique structural characteristics [177]. Moreover, these nanopores can be rationally tuned by chemical transformation at the outer or inner surface and, therefore, modulate the specific properties of MSF-electrodes, such as charge, selective permeability, hydrophobicity/hydrophilicity, and electrocatalysis [175]. For example, the conductivity of MSF can be improved by functionalizing with AgNPs [177]. Table 4 summarizes selected representative examples of electrochemical biosensing methods using multifunctional silica nanomaterials.

As deduced from Table 4, mesoporous nanomaterials, and in particular MSNs, are mainly used in electrochemical affinity bioassays (mostly aptasensors [149], but also immunosensors and DNA sensors) and much less in catalytic biosensors.

It is remarkable the versatility of silica nanomaterials to be used as electrode modifiers, nanocarriers of signaling elements or tracing tags. In particular, MSNs and MSF can be used in Janus formats as nano-engineered anisotropic supports for the toposelective co-immobilization of different (bio)molecules [183,185,186]. In addition, taking advantage of their porous structure, they can be used to accommodate molecules or other cargoes in the pore voids, acting as molecular gates once provided with stimulus-responsive gate-like molecular, supramolecular, or bio-molecular ensembles at the external surface able to block the pores and only triggered by a predefined chemical or biochemical stimulus, allowing on-command delivery of entrapped cargo molecules (which act as a signaling agent) from the uncapped pores [148,164,188,190,191,197].

The rationale and relevant characteristics of the different biosensing strategies will be discussed in more detail below. However, it is important to note here that the use of these multifunctional silica nanomaterials has shown great potential for the development of electrochemical biosensors for the determination of a wide variety of molecular targets (protein biomarkers, toxins, drugs, heavy metals, bacteria, specific gene regions, and other clinically relevant molecules) in food (rice, maize, wheat, kidney, muscle, milk, honey, soft drinks), environmental (water), and clinical (bacterial lysates, pharmaceuticals, serum, plasma, urine, and cells) samples. So far, these biosensors have been implemented mainly on conventional electrodes and much less on screen-printed electrodes (SPEs). Voltammetric techniques such as CV, DPV, SWV, LSV, ASV, and in a lesser extent, amperometry and frequency-dependent (EIS) techniques, have been used for the detection.

Regarding MSNs, they have been employed in electrochemical biosensing as Janus-type particles, as gated systems or, exploiting their excellent properties in individual or hybrid formats, as electrode modifiers or nanotransporters of signaling elements. In addition, some works exploited two types of SiO_2_ NPs in the same strategy [183,192].

**Figure 11:**
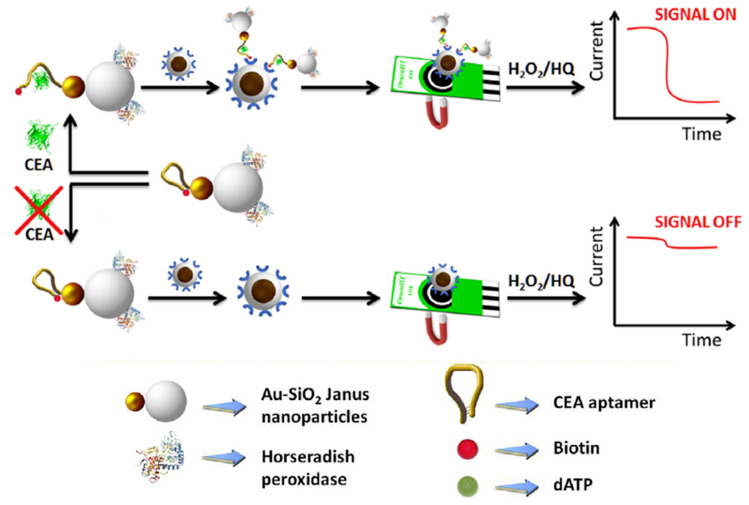
Schematic display of the aptasensing strategy reported for the amperometric determination of CEA involving the use of aptamer and HRP bifunctionalized Au-MS JNPs, Av-Fe_3_O_4_@SiO_2_ NPs and amperometric detection at SPCEs. Reprinted and adapted from [183] with permission.

Villalonga’s group [185] used dual functionalized Janus type Au-MSNs (Au-MS JNPs) both as electrode supports and nanotransporters of signaling elements in enzymatic and affinity biosensors. In their first proof-of-concept, they employed Au-MS JNPs modified with Strep and HRP on the Au and MS faces, respectively, as an electrochemical biorecognition-signaling system using a biotin-modified gold disk electrode and monitoring the affinity reaction by EIS and CV in the presence of [Fe(CN)_6_]^4-/3-^ [183,185,186]. The same group used Au-MS JNPs modified with GOx and HRP on the Au and MS faces, respectively, to modify a SWCNTs-coated GCE for the enzymatic biosensing of glucose [186]. The amperometric biosensor achieved a LOD of 360 nM and was utilized for the analysis of commercial soft drinks. Furthermore, they have reported the use of Au-MS JNPs, modified with HRP and a DNA hairpin aptamer dually modified with biotin and thiol on the MS and Au faces, as a biorecognition-signaling system, as well as avidin-modified Fe_3_O_4_@SiO_2_ NPs as solid supports for the construction of an electrochemical aptasensor for the determination of carcinoembryonic antigen (CEA) [183]. As can be seen in Figure 11, the DNA hairpin aptamer was unfolded selectively in the presence of CEA, leaving biotin accessible for recognition by avidin-modified Fe_3_O_4_@SiO_2_ NPs. The resulting magnetic bioconjugates were captured on the surface of SPCEs and amperometric detection was performed in the presence of the H_2_O_2_/HQ system providing large currents proportional to the concentration of CEA due to the high concentration of aptamer and bifunctionalized HRP Au-MS JNPs attached to the Av-Fe_3_O_4_@SiO_2_ NPs. This aptamer, involving a smart combination of two different types of SiO_2_ NPs, achieved a LOD of 1.2 pM and was used for the determination of CEA in a spiked commercial human serum.

**Figure 12:**
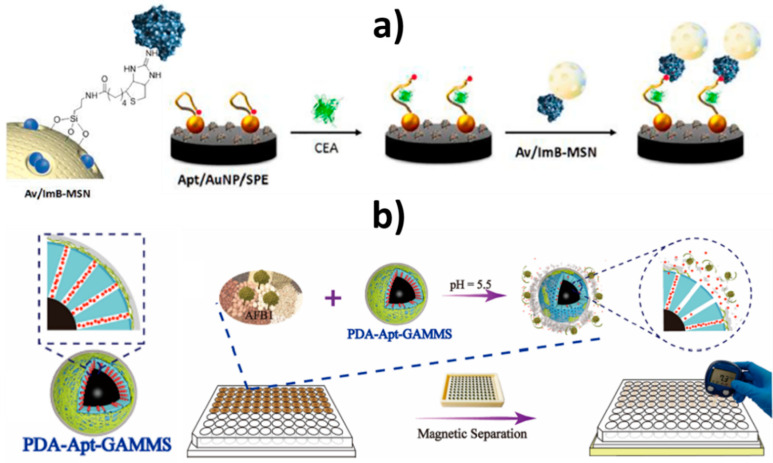
Display of the electrochemical aptamer-based biosensing strategies using MSNs as gated nanomaterials. (**a**) MB-loaded Av/ImB-MSN as nanocarriers for signal amplification for the determination of CEA. (**b**) Glucose-loaded PDA-Apt-GAMMS for the determination of AFB1 using a PGM. Reprinted and adapted from [187] (**a**) and [191] (**b**) with permission.

MSNs have been used as gated nanomaterials in DNA- or aptamer-based biosensing strategies for the determination of Hg^2+^, toxins, and cancer biomarkers. In addition, methylene blue (MB)-loaded MSNs have been employed as electrode modifiers or nanocarriers of signaling elements in the development of aptasensors for the determination of ochratoxin A (OTA) [164] and CEA [187], respectively. In the method reported by Muthamizh et al., MB was released from the OTA aptamer-capped MSNs in the presence of OTA. Jiménez-Falcao et al. exploited the MB-loaded MSNs capped with a pH-sensitive avidin/imminobiotin (Av/ImB-MSN) ensemble for signal amplification in the preparation of an aptasensor through assembling of dual biotin and thiol-labeled DNA hairpin aptamer on AuNPs-SPCE. The unfolding of the aptamer in the presence of CEA allowed biotin to be associated with the avidin-capped mesoporous nanocarrier (Figure 12a). The release of MB was achieved by incubating the Av/ImB-MSN in acidic medium due to pH-mediated disruption of the imminobiotin/avidin complex. MB released was monitored in both methods by DPV.

On the other hand, glucose-loaded MSNs involving personal glucometer (PGM) readout have been described [179,188,189,190,191]. For example, the use of glucose-loaded DNAzyme-capped MSNs was reported by Fu et al. for the determination of Pb^2+^ [188]. Wang et al. [189] combined glucose-loaded wrapping DNA-capped MSNs and the extension reaction assisted by deoxyribonucleotides (dNTPs) in the design of an electrochemical method to detect telomerase activity in HeLa cells achieving the possibility to detect just 80 cells mL^−1^. Upon addition of telomerase and dNTPs, the DNA assembled on MSNs was extended along the 5′→3′ direction, resulting in the formation of repeated DNA sequence at the 3′ end of telomerase which hybridized with the sequence at the 5’ end forming a rigid hairpin-like DNA structure and leading to the detachment of the wrapping DNA from the surface of aminated MSN and therefore, to the release of the pore-entrapped glucose molecules which were monitored at a PGM.

Liang et al. reported the determination of Hg^2+^ by using the target-responsive release of glucose from single-strand wrapping DNA sealed MSNs [190]. In the presence of Hg^2+^ and the assistant DNA, the T–Hg^2+^–T base-pairing detached wrapping DNA from MSNs and induced the formation of a wrapping and assistant DNA duplex able to be recognized by Exo III, which can digest the wrapping DNA and Hg^2+^, leading to the continuous detachment of wrapping DNA from MSNs and releasing glucose from MSNs.

Aptamer-capped MSNs with glucose loaded on GO nanosheets attached to the aptamer through π-stacking interactions were used for a sensitive determination of AsO_3_^−^ in water samples with a LOD of 2.3 pg mL^−1^ [179]. Moreover, glucose-loaded “dual gates” aminated magnetic mesoporous silica nanocomposites (GAMMS) bearing polydopamine (PDA)-aptamer (Apt) two-tier shells (PDA-Apt-GAMMS) were employed for the determination of aflatoxin B1 (AFB1) (Figure 12b) [191]. The pores of the AMMS were opened and MB molecules released upon the introduction of the target molecules under acidic conditions due to the PDA self-degradation and the specific Apt-target reaction. As it can be deduced from Table 4, the strategies described using MSNs loaded with MB [164,187] or glucose [179,188,189,190,191] as gated systems led to sensitive biosensing with applicability to the analysis of food or clinical samples even at the point of care.

Different MSNs were exploited as electrode modifiers in the development of different aptasensors [152,154,163,165,169,173,181]. For example, Huang et al. developed a label-free codeine aptasensor at an Au-MSNs-modified GCE [152]. Using EIS in the presence of [Fe(CN)_6_]^4^^−/3^^−^, this aptasensor provided a LOD of 3 pM. A chloramphenicol (CAP) aptasensor was developed by immobilizing the specific aptamer on an AuNPs/SBA-15@DABCO-modified SPCE and using hemin, which interacted with the guanine bases of the aptamer, as an electrochemical indicator [169]. The DPV signal of the hemin attached to the aptamer was amplified in the presence of CAP due to the stabilization of the folded aptamer. The same authors reported an epirubicin aptasensor monitoring by linear sweep voltammetry (LSV) the analyte linked to the aptamer immobilized on AuNPs/Fe_3_O_4_@SiO_2_/DABCO/SPCEs [181]. Both aptasensors achieved low LODs (4.0 nM and 0.04 μM for CAP and epirubicin, respectively) and were used for the analysis in spiked human blood serum.

**Figure 13:**
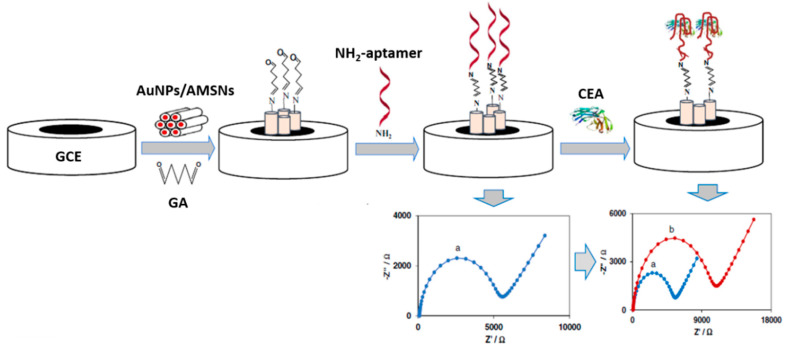
Impedimetric aptasensor for CEA using an AuNPs/AMSNs-GCE as transduction interface. Reprinted and adapted from [154] with permission.

Zare’s group [154,163] used amino-functionalized MSNs (AMSNs) or AuNPs/AMSNs as GCE modifiers to construct aptasensors for the voltammetric determination of hemin and hemoglobin (Hb) and the impedimetric determination of CEA (Figure 13). In both biodevices, the specific NH_2_-aptamer was covalently immobilized using glutaraldehyde (GA) on the AMSNs-modified GCE. The voltammetric aptasensor measured by DPV, the reduction current of hemin or Hb in the presence of oxygen [163]. Both aptasensors were employed to perform the determination of Hb and CEA endogenous concentrations in real blood and serum samples.

A very sensitive aptasensor (LOD of 10 fM) for AFM1 was prepared by immobilizing a toluidine blue (TB)-labeled aptamer on a GCE modified with chitosan-modified graphene quantum dot/dendritic fibrous nanosilica functionalized by amine groups (GQDs-CS/KCC-1-NH_2_-Tb) by electrodeposition [173]. This bioscaffold, in which the electrochemical signal of TB measured by DPV decreased as the concentration of AFM1 increased due to the prevention of the electronic transfer, was useful to perform the determination in milk samples.

**Figure 14:**
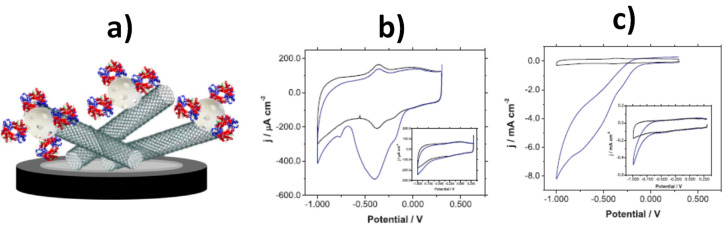
(**a**) Schematic display of the GCE/MWCNTs-MCM41-Hb bioplatform. Cyclic voltammograms obtained in a N_2_-saturated 0.050 M phosphate buffer solution pH 7.00 in the absence (black) and presence (blue) of 1.5 × 10^−2^ M NaNO_2_ (**b**) and 0.100 M TCA (**c**) at GCE/MWCNTs-MCM41-Hb. Inset: corresponding CVs obtained at GCE/MWCNTs. Reprinted and adapted from [165] with permission.

Eguílaz et al. [165] reported a novel biosensing platform involving a hybrid bioconjugate composed of Nafion-coated multiwalled carbon nanotubes (MWCNTs) and mesoporous silica MCM-41 nanoparticles functionalized with Hb as GCE modifier (Figure 14a). The prepared MWCNTs-MCM41-Hb nanostructured architecture combined the high surface area, biocompatibility and protein loading capacity of MCM-41 NPs with the high surface area and catalytic properties of MWCNTs, and allowed the direct electron transfer (DET) between Hb and the electrode surface (Figure 14b,c). The GCE/MWCNTs-MCM-41-Hb was successfully used as a third-generation biosensor for amperometric determination of NO_2_^−^ and trichloroacetic acid (TCA).

**Figure 15:**
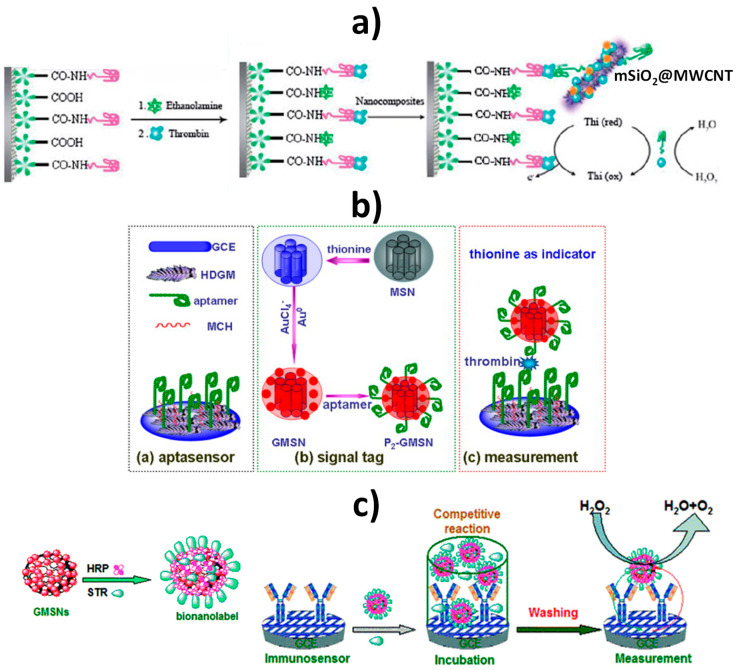
Sandwich aptasensors for thrombin developed at a PAMAM–rGO-GCE using mSiO_2_@MWCNT decorated with Thi, platinum nanoparticles (PtNPs), and hemin/G-quadruplex bioelectrocatalytic complex as bionanolabels (**a**) or a HDGMs-modified GCE using Thi-GMNs as signaling tags (**b**). (**c**) Competitive immunosensor for STR developed at a TRSiNs (Thi)-GCE using GMSNs carrying STR-BSA and HRP. Reprinted and adapted from [178] (**a**) [151] (**b**) and [192] (**c**) with permission.

MSNs have been employed as labels in affinity electrochemical biosensors [151,178,192]. For example, mSiO_2_@MWCNT were used as nanocarriers of Thi, platinum nanoparticles (PtNPs) and hemin/G-quadruplex bioelectrocatalytic complex to achieve amplification of responses in the design of a sandwich-type pseudobienzyme aptasensor for the determination of thrombin. A poly(amidoamine)-reduced graphene oxide-modified GCE (PAMAM–rGO-GCE) was prepared as the biosensor platform to enhance the surface area for the immobilization of abundant primary aptamers and to facilitate electron transfer from Thi to the electrode (Figure 15a) [178]. Electrochemical detection by DPV in the presence of H_2_O_2_ provided a LOD of 50 fM and the aptasensor was utilized to perform the determination in spiked human serum.

Tang et al. [151] designed another sandwich-based aptasensor for thrombin by combining GCE modified with densely packed hierarchical dendritic gold microstructures (HDGMs) and Thi nanogold-decorated MSNs (Thi-GMSNs) as signaling tags (Figure 15b). The aptasensor achieved a LOD of 15 fM and exhibited successful applicability in spiked fetal calf serum through DPV detection of Thi.

The use of GMSNs as nanotransporters of STR-BSA and HRP has been proposed in the development of a competitive immunosensor for streptomycin (STR) implemented on GCE modified with three-dimensional organosilica nanostructures (TRSiNs) doped with Thi (Figure 15c) [192]. This bioplatform allowed a LOD of 5 pg mL^−1^ and was used for the determination of the antibiotic in different contaminated samples through the electrochemical monitoring of Thi by DPV.

**Figure 16:**
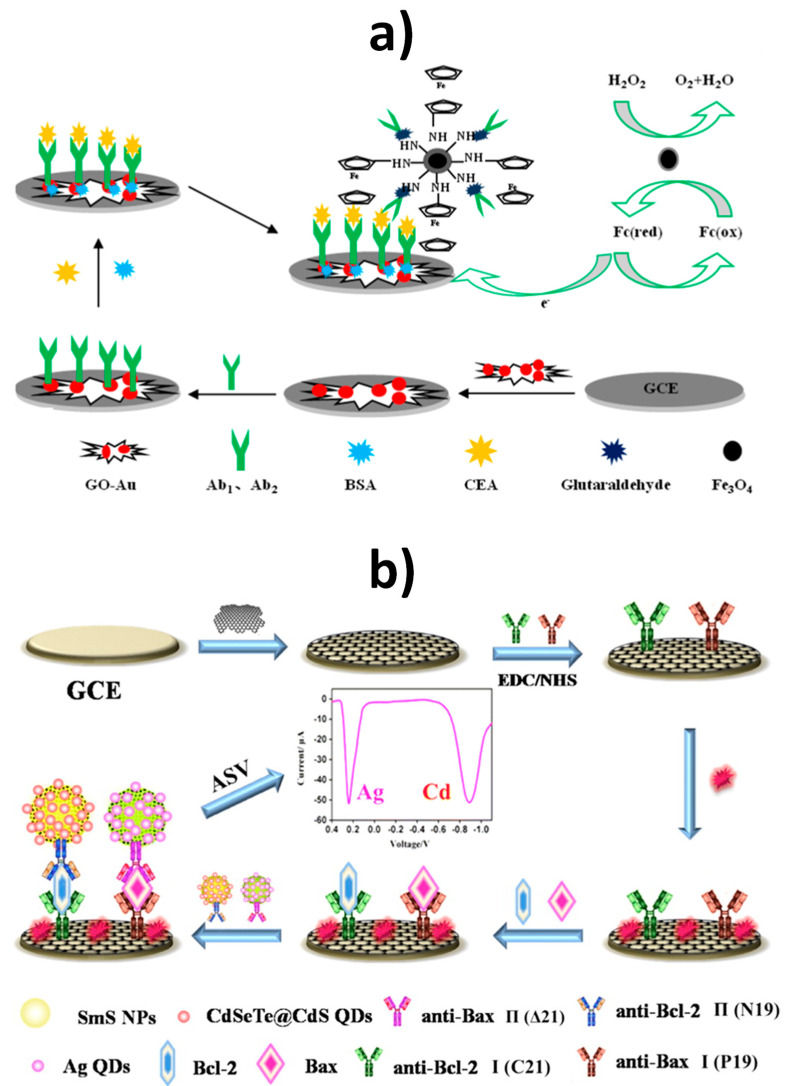
Sandwich electrochemical immunosensors developed for: (**a**) the determination of CEA using Fe_3_O_4_@SiO_2_–Fc–DAb/HRP nanobioconjugates and (**b**) the simultaneous determination of Bcl-2 and Bax using mesoporous SiO_2_ decorated with DAb and CdSeTe@CdS quantum dots (QDs) or Ag nanoclusters (NCs). Reprinted and adapted from [182] (**a**) and [180] (**b**) with permission.

Feng et al. [182] employed Fe_3_O_4_@SiO_2_–NH_2_ as nanocarriers of Fc-COOH and DAb (Fe_3_O_4_@SiO_2_–Fc–DAb/HRP) to prepare a sandwich-type immunosensor for CEA using a GO–AuNPs-modified GCE as electrode platform (Figure 16). The designed immunosensor showed a very LOD of 0.0002 ng mL^−1^, which was attributed in part to the large amount of Fc-COOH loaded to Fe_3_O_4_@SiO_2_–NH_2_ which could hasten the H_2_O_2_ decomposition monitored by DPV. The immunosensor was successfully applied in spiked human serum samples. Another sandwich immunosensing strategy was developed by Zhou et al. [180] for the simultaneous detection of B-cell lymphoma 2 (Bcl-2) and Bcl-2-associated X protein (Bax) proteins at a rGO-GCE using mesoporous SiO_2_ decorated with detector antibody (DAb) and CdSeTe@CdS QDs or Ag nanoclusters (NCs) as nanocarriers of signaling elements. The simultaneous determination was performed indirectly by the detection of oxidation peak currents of Cd and Ag using anodic stripping voltammetry, allowing LOD values of ∼0.5 fmol. This immunosensor was applied to the determination of the Bcl-2 expression and in drug-treated cells (chronic myeloid leukemia K562 cells), showing the ability to detect only 1 × 10^3^ cells.

**Figure 17:**
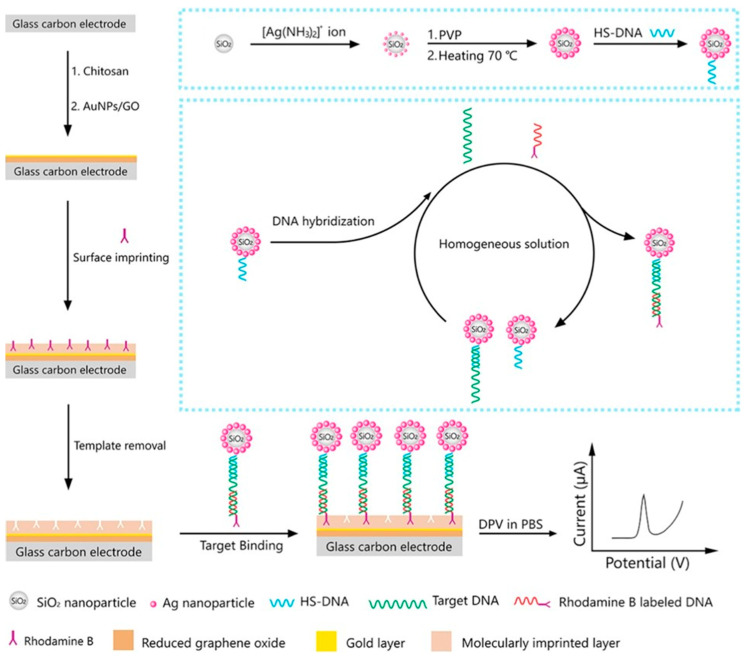
DNA biosensor for the determination of *BRCA-1* using DNA-modified SiO_2_@AgNPs as tracing tags. Reprinted and adapted from [193] with permission.

SiO_2_ NPs were also exploited as tracing tags for signal amplification in electrochemical DNA sensors. You et al. [193] reported the use of DNA-modified SiO_2_@AgNPs as tracing tags in a DNA biosensor for breast cancer susceptibility (*BRCA-1*) gene (Figure 17). The biosensor involved an AuNPs-GO-modified GCE coated with a layer of a MIP synthesized using rhodamine B (RhB) as a template. A sandwich hybridization was performed in a homogeneous solution between the target DNA, the DNA-modified SiO_2_@AgNPs, and an RhB-labeled probe. The resulting SiO_2_@Ag/dsDNA/RhB was specifically recognized by MIPs via the interaction between imprinting cavities and RhB. Using DPV of RhB, the DNA biosensor achieved a low LOD of 2.53 fM and was employed for the analysis of serum.

**Figure 18:**
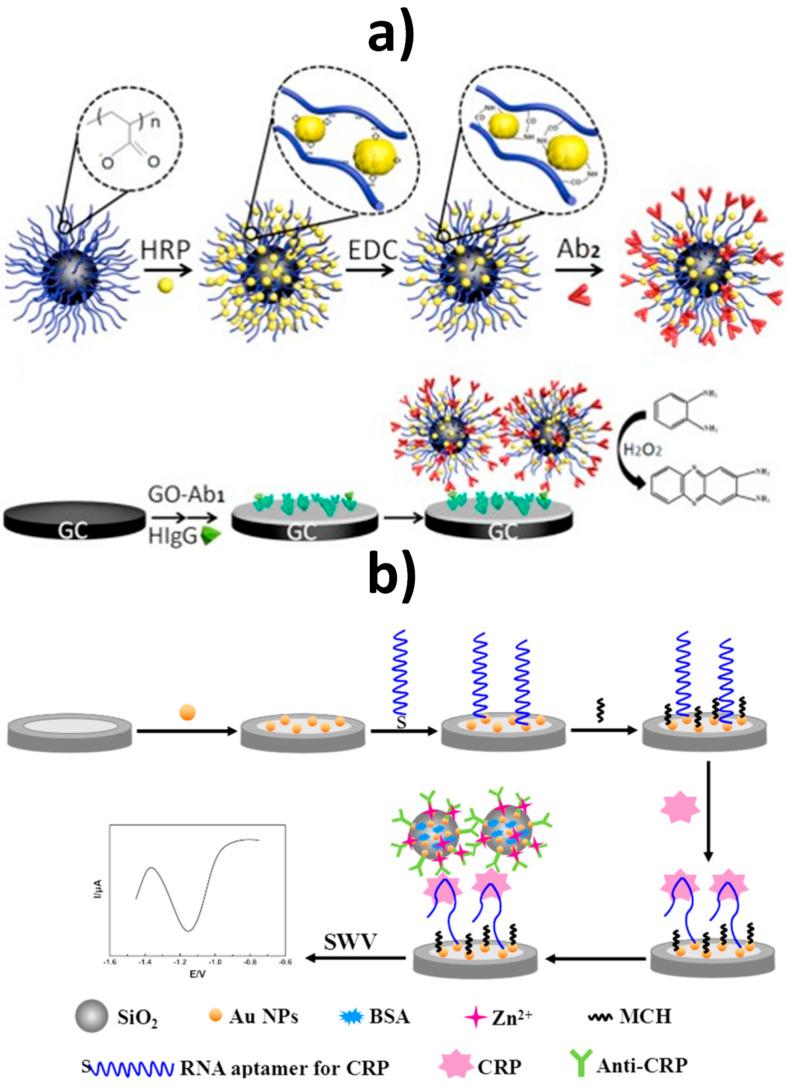
Use of DAb and HRP-loaded SiO_2_-SPAABs (**a**) and AuNPs-Si MSs decorated with Zn^2+^ and DAb as labels (**b**) in the preparation of an electrochemical immunosensor and an aptasensor for the determination of HIgG and CRP, respectively. Reprinted and adapted from [194] (**a**) and [195] (**b**) with permission.

Zhao et al. reported an electrochemical sandwich immunosensor for human IgG (HIgG) using a GO-GCE as transducer and DAb and HRP-loaded silica-poly(acrylic acid) brushes (SiO_2_-SPAABs) as labels (Figure 18a) [194]. The SiO_2_-SPAABs NPs exhibited superior properties over the conventional ones regarding the three-dimensional structure, flexibility, and high-abundant capacity of enzyme. SiO_2_ NPs modified with SPAABs, in which the SiO_2_ nanoparticles served as a robust and versatile core for surface modification and surface-initiated reversible addition–fragmentation chain transfer (RAFT) polymerization process of SPAABs, provided good control of molecular weights, rich content of carboxyl groups, and thickness of the poly(acrylic acid) (PAA) brushes [194]. It is important to note that HRP could only be immobilized on the surface of MSNs due to the space steric hindrance. The affinity reaction was followed by DPV using the OPD+H_2_O_2_ system and the immunosensor achieved a 6.70-fold higher sensitivity than conventional ELISA assays. The LOD was 50 pg mL^−1^ and the immunosensor was applied to the analysis of HIgG in serum samples.

It is worth noting that although mesoporous silica provides a larger surface than the pure silica nanoparticles for the immobilization of biomolecules and improve the sensitivity of the resultant biosensor, silica microspheres (Si MSs) have also been exploited in some electrochemical biosensors [151,192]. For example, an attractive electrochemical aptasensor was reported for the determination of CRP detection using Si MSs functionalized with AuNPs and decorated with Zn^2+^ and DAb as immunoprobes (Figure 18b). The aptasensor was constructed by self-assembling a thiolated aptamer at an AuNPs-GCE. SWV was employed to monitor the reduction of Zn^2+^, achieving a low LOD of 0.0017 ng mL^−1^. The sensor was successfully used in the analysis of real serum samples.

**Figure 19:**
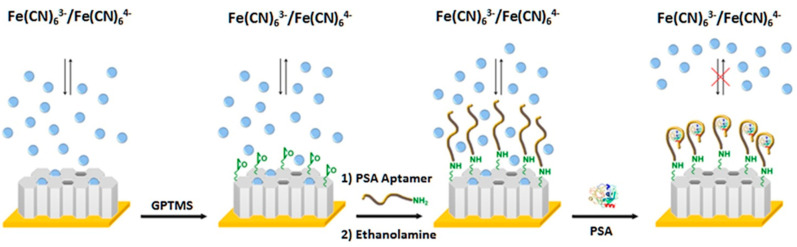
Label-free electrochemical aptasensor for PSA detection developed at an MSF-AuE. Reprinted and adapted from [176] with permission.

Regarding MSFs, to date, they have always been used as a modifier of conventional gold electrodes in catalytic or affinity biosensing methods to target transglutaminase activity (TGase) [196] Cu^2+^/ascorbic acid (AA) [195], *E. coli* 16S rRNA [175], prostate-specific antigen (PSA) [176], and streptomycin (STR) [177]. Villalonga’s group proposed an electrochemical assay to quantify TGase activity via the enzyme-controlled diffusion of Fe(CN)_6_^3−/4−^ through amino-functionalized nanochannels of a MSF-AuE [196]. The nanochannels were selectively gated by the catalytic action of TGase in the presence of the glutamine-donor substrate N-benzyloxycarbonyl-L -glutaminylglycine (CBZ). This effect produced a decrease in the accessibility of the electrochemical probe to the modified electrode surface and, accordingly, in the signals recorded by CV and EIS. Further works from the same group [174,175,176] used MSFs as gated systems involving specific DNAzymes, DNA, or aptameric sequences as biomolecular gatekeepers. In all these methods, the presence of the target analyte led to the opening of the nanopores and the free diffusion of the [Fe(CN)_6_]^4−/3−^ ions through the nanochannels of the MSF to the electrode surface which was monitored by voltammetry. A representative example of these methods is displayed in Figure 19. These scaffolds showed interesting applications such as the direct determination of the *Escherichia coli* 16S ribosomal RNA gene in raw lysate solutions in only 45 min [175].

Roushani and Ghanbari [177] reported a sandwich aptasensor for STR at an MSF-AuE. In this case, the MSF was used to increase the surface area to bind a large amount of aptamer and to improve the electrical conductivity of the electrode. Binding of STR to the aptamer hindered the diffusion of the redox probe ([Fe(CN)_6_]^4−/3−^), monitored by DPV, through the nanochannels of the MSF.

## 5. Overview and Look to the Future

Currently, most researchers are aware of the major role played by nanomaterials in the development of biosensors and electrochemical biosensing strategies providing improved performance in terms of sensitivity, selectivity, reproducibility, and operational stability. Multifunctional and multifunctionalized nanomaterials further enhance the significant advantages imparted by conventional nanomaterials because they are able to achieve a mixed/combined effect or multiple functions using a single nanosystem. This review article has overviewed, through critically discussing recent developments, the nanomaterials used in designing electrochemical biosensors and biosensing approaches that either exhibit multifunctionality by themselves or combined with other materials to display combined or synergic properties.

Because of their unique characteristics and the excellent properties, we have focused on multifunctional metal (MNPs, metal oxides, QDs, 2D-TMCs, TMOs, and Mxenes), carbon and silica-based (MSNs, MSF) nanomaterials. Magnetic and silica microspheres are also briefly discussed. These nanomaterials have been used as electrode modifiers, nanocarriers of signaling elements or tracing tags in electrochemical (catalytic and affinity) biosensors and biosensing approaches. The excellent capabilities of these nanomaterials have been profited in connection with different bioreceptors (enzymes, antibodies, aptamers, and DNA probes), assay formats, electrodes (conventional and SPEs), and detection techniques (label-free and label-based strategies), opening up the improved determination of a wide variety of relevant molecular targets in the clinical, food, and environmental fields and in very diverse matrices through simple and short protocols.

In the field of electrochemical biosensors, the following properties/opportunities of the functional nanomaterials reviewed in this article deserve to be highlighted: (i) the large surface area, porosity, electrical conductivity, electrocatalytic activity, good biocompatibility, tunable shape and composition, adsorption ability, high density of active surface sites, and pseudo-enzymatic behavior of MNPs. These properties have allowed the enhancement of the capacity for the stable and well oriented immobilization of biomolecules, the improvement of the charge transport rates, the replacement of natural enzymes, and the achievement of DET between redox proteins and bulk electrode materials; (ii) the tunable physical and chemical properties, electrochemical performance, high surface area, biocompatibility, enzyme-like behavior, adsorption ability, and electrocatalytic activity of carbon-based nanomaterials; and (iii) the biocompatibility, tunable size, shape, and pore structure/diameter using simple and affordable fabrication procedures, thermal, chemical, and mechanical stability, suspendability in aqueous solution, high porosity (large specific surface area and interior space), uniform size, and three-dimensional structure, made of highly open interconnected spaces and easiness to be functionalized for encapsulating or immobilizing a large amount of payloads, which have allowed their unique use in Janus formats as nano-engineered anisotropic supports or as gated systems, of silica-based materials.

It is clear that progress in this field will inevitably be assisted by both new uses and rational designs and synthetic methodologies of novel multifunctional nanomaterials and their combinations in an easy, reproducible, and chemical friendly manner. This will lead to improved capabilities, for example, to achieve greater enzymatic activity and, therefore, minimizing the use of unstable, expensive, and difficult to manipulate biological materials

It is important to mention that electrochemical biosensing making use of multifunctional nanomaterials has to face also all the challenges that the future of electrochemical biosensors has in front with a view to achieve their wide commercialization and integration in everyday life. For example, their use should be generalized for multiplexed and multiomic determinations and fully implemented and validated for the analysis of a large number of real samples by different users while ensuring robustness and reliability at different settings.

As far as we know, there are some nanobiotechnological companies that commercialize multimodal/multifunctional nanomaterials, mainly for their use in drug delivery, therapeutic applications, medical devices (including tissue engineering), and basic research. However, up to date, we do not know any commercial product in bio-electrochemistry by exploiting them.

In this sense, continuous advances have opened opportunities for the application of the new generation of multifunctional nanomaterials in a variety of disciplines. Nevertheless, there is still a huge gap between research with these nanomaterials in the laboratories and their large-scale production and commercialization. This gap contains a litany of challenges to address. One of the most important lays in their robust production and scale-up using laboratory processes consistent with current manufacturing capabilities. Moreover, these nanomaterials will achieve commercial success only when compelling applications are found and adopted. For now, their applications in bio-electrochemistry are still at the concept level and require much more basic research to be incorporated into a viable product. Another major challenge includes performing rigorous studies to evaluate the toxicity of these nanomaterials over the next 5–15 years and describing models that predict their effects on human health and the environment to ensure their public acceptance. In addition, researchers should take a proactive role in translating laboratory findings into viable and marketable technology, which requires them to consider future commercialization at an early stage in their research and act accordingly. Moreover, this long and hard process to convert these basic research discoveries into marketable products must be necessarily supported at the global level with funding and support both from governments, which provide the early-stage investment in this high-risk, high-payoff technology, and private or corporate investment, in order to carry the process to fruition [198,199].

Nevertheless, it can be predicted that the excellent advantages and versatility exhibited by the multifunctional nanomaterials reported so far and those envision to come will undoubtedly lead to new and unpredictable capabilities and opportunities. The combination of multifunctional nanomaterials with the use of other receptors less explored to date, such as switch-based receptors, and with advances in surface chemistry, allowing anti(bio)fouling behavior and ability to perform the determinations in continuous mode and even in vivo in a reagentless and calibration-free manner, will booster also the capabilities and opportunities. Other future perspectives include the use of multifunctional nanomaterials to improve the characteristics of flexible and wearable biosensors, which are highly sought. It is clear that there is a lot of space to be explored and important challenges to be addressed through properly coordinated multidisciplinary efforts. Nevertheless, it also clear that all the achievements made, the intense research activity and the experience gained in this field are enough to ensure the binomial multifunctional nanomaterials-electrochemical biosensing will continue to gain ground due to the broad potential, tremendous opportunities, versatility to adapt the changing societal needs, and the challenging applications that need to be successfully addressed.

## Figures and Tables

**Table 1:** Electrochemical biosensing methods involving multifunctional metal-based nanomaterials.

Electrode	Nanomaterial/Role	Biosensing Approach/Format	Detection Technique	Target Analyte	LR/LOD	Sample	Ref.
-Metal nanoparticles (MNPs)-							
CILE	CILE modified with AuNT and HRP and coated with nafion/Direct electron transfer of HRP	Enzyme-based	CV	TCA and NaNO_2_	TCA: 1.0–250 mM/0.33 mM NaNO_2_: 1.6–66.0 mM/0.53 mM	Facial peel solution	[7]
GCE/AuNPs	Self-assembly of GS-IL/AuNRs through thiolated sol–gel matrix Positively charged GS-IL was used for GOx immobilization GS-IL and Au NRs acted as high electroactive catalyst.	Enzyme-based	Amperometry −0.2 V (H_2_O_2_)	Glucose	1–764 μM/0.38 µM	Serum and brain microdialysate	[8]
SPCE	PtNPs-MWCNTs-GOx/Electrode modifier to enzyme immobilization and electrocatalytic	Enzyme-based	Amperometry −0.5 V (H_2_O_2_)	Glucose	65.8–260.6 μg mL^−1^/35.0 μg mL^−1^	White grapes and glucose tablets	[9]
GCE	Electroactive polymer nanospheres synthesized from polymerization of ferrocenedicarboxylic acid/nanocarriers of Ab2, PtNPs, and hemin/G-quadruplex bioelectrocatalytic complex	Antibody based/Sandwich	DPV	AFP	0.1 pg mL^−1^–100 ng mL^−1^/0.086 pg mL^−1^	Human serum	[11]
GCE	GCE modified with Au@Thi/GO loaded with cAb PtCu@rGO/g-C_3_N_4_ conjugated with DAb	Antibody based/Sandwich	Amperometry −0.4 V	PSA	50 fg mL^−1^–40 ng mL^−1^/16.6 fg mL^−1^	Human serum	[14]
SPCE-AuNPs	PEGylated nanoCeO_2_ conjugated to thiolated-cAb	Antibody based/Direct	CV ([Fe(CN)_6_]^3−/4−^)	HER2	1–500 pg mL^−1^/34.9 pg mL^−1^	Serum from breast cancer patient	[15]
GCE	Cu_2_O@CeO_2_-AuNP/nanocarrier of cAb and electrocatalytic activity reduction of H_2_O_2_	Antibody based/Direct	Amperometry −0.4 V	PSA	0.1 pg mL^−1^–100 ng mL^−1^/0.03 pg mL^−1^	Human serum	[17]
GCE-AuNP	Co_3_O_4_@CeO_2_-Au@Pt/nanocarrier of DAb and enzyme-mimetic label	Antibody based/Sandwich	Amperometry −0.2 V	SCCA	100 fg mL^−1^–80 ng mL^−1^/33 fg mL^−1^	Spiked human serum	[18]
GCE	AuBP@Pt/Electrode modifier AuPd-PDA nanozyme/DAb nanocarrier and electrocatalytic activity reduction of H_2_O_2_	Antibody based/Sandwich	Amperometry −0.25 V	APOE4	0.05–2000 ng mL^−1^/15.4 pg mL^−1^	Commercial goat serum	[19]
SPCE	SPCEs modified with a 3D network PAMAM-AuNPs nanocomposite/to immobilize cAb	Antibody based/Sandwich	Amperometry −0.2 V (H_2_O_2_ + HQ)	tau protein	6–5000 pg mL^−1^/1.7 pg mL^−1^	Raw plasma and brain tissue from healthy and post mortem diagnosed AD patients	[21]
dSPCE	3D-Au-PAMAM on *p*-ABA-dSPCE for the immobilization of cAb	Antibody based/Sandwich	Amperometry −0.2 V (H_2_O_2_ + HQ)	tau and TDP-43 proteins	Tau: 0.008–5.0 ng mL^−1^/2.3 pg mL^−1^ TDP-43: 0.043–15.0 ng mL^−1^/12.8 pg mL^−1^	Plasma and brain tissue extracts from healthy and NDD patients	[22]
	Fc-IL-CHO/AuNP-PAMAM-based platform was used to immobilize the cAb	Antibody based/Direct	DPV (Fc)	α-fetoprotein	0.05–30 ng mL^−1^/0.02 ng mL^−1^	Human serum samples	[23]
GCE	rGO-TEPA as electrode modifier and Ag@CeO_2_ nanocomposite as labels	Antibody based/Sandwich	Amperometry −0.4 V (H_2_O_2_)	TGSF	0.500–100 pg mL^−1^/0.2 pg mL^−1^	Spiked human serum	[25]
GCE	Poly-L-lysine/Electrode modifier DNA-Ag/Pt NCs/peroxidase-mimicking activity	Aptamer based/Sandwich	Amperometry −0.1 V (H_2_O_2_ + TMB)	VEGF	6–20 pM/4.6 pM (175 pg mL^−1^)	Human serum	[27]
GCE	TDNs–aptamer1 as recognition probe Mn_3_O_4_-Pd@Pt-aptamer2-HRP as nanozymes and nanoprobe	Aptamer based/Sandwich	DPV (H_2_O_2_ + HQ)	HER2	0.1–100.0 ng mL^−1^/0.08 ng mL^−1^	Human serum	[29]
AuE	Signal amplification by CHA reaction PtPdNPs/HRP as mimic nanozyme	Aptamer based/Displacement	Amperometry −0.45 V (H_2_O_2_ + TMB)	Mucin 1	100 fg mL^−1^–1 ng mL^−1^/16 fg mL^−1^	Human serum	[31]
GCE	MWCNT/Electrode modifier for ssDNA immobilization Ag-DNCs-labeled DNA probe	DNA-based/Direct	Ag+ by DPASV	DNA hybridization	10–300 pM/0.78 pM	-	[32]
GCE	AuNPs/Electrode modifier for immobilization of S-DNA probe MPBA-AgNPs as label	DNA-based/Direct	LSV	miRNA-21	0.1–50 fM/20 aM	Serum	[33]
Gold	MPBA-biotin-AuNPs and Strept-AP as labels	Aptamer-based/Direct	DPV (*p*APP)	rHuEPO	0.02–2 pM/8 fM	-	[34]
GCE	SA-PPy/AuNPs/Electrode modifier for hairpin immobilization Signal amplification by CHA reaction and Cu^2+^/Fe^3+^ catalytic reaction	Aptamer-based/Displacement	SWV	miRNA	1 fM–1 nM/0.34 fM	Spiked serum	[36]
Au micro-gap electrode	pRhNPs/Electrode modifier and T4 DNA aptamer 3-way junction-Ag^+^ immobilization	Aptamer-based/Direct	EIS ([Fe(CN)_6_]^3−/4−^)	T4	up to 11.41 pM/10.33 pM	Human serum	[37]
GCE	Self-assemble AuNPs superlattice through pPPy for sDNA immobilization/Electrode modifier TB as tracing tag	DNA-based/Direct	DPV	miRNA-21	100 aM–1 nM/78 aM	Human serum	[38]
GCE	poly(FBThF) and Ag-rGO-NH_2_ nanocomposite/AChE immobilization and catalytic activity	AChE	Amperometry (ATCl)	Malathion trichlorfon	Malathion: 0.099–9.9 μg L^−1^/0.032 µg mL^−1^ Trichlorfon: 0.0206–2.06 μg L^−1^/0.001 μg L^−1^	Food	[43]
Bare gold	AuNPs functionalized with aptamer/mimetic HRP activity	Nanozyme/target-induced replacement of the aptamer	DPV (H_2_O_2_, thi)	kanamycin	0.1–60 nM/0.06 nM	Honey samples	[44]
GE	PtRu nanoparticles/Electrode modifier and for AO and AMO immobilization	Nanozyme-based	Amperometry −0.32 V (H_2_O_2_)	Ethanol Methylamine	Ethanol: 25–200 μM Methylamine: 20–600 μM	Red wine	[45]
-	CeO_2_ NPs/GOx mimicking activity	Nanozyme/TdT induced the aggregation of CeO_2_	PGM signal	Glucose	up to 100 U mL^−1^/0.7 U mL^−1^	Human blood	[46]
-Multifunctional MNPs involving ordered nanostructures-							
GCE/AuNP@MWCNTs	bimetallic Cu-TCPP(Fe) nanosheets to immobilize PtNi nanospheres/nanocarriers of detection antibody and electrocatalytic H_2_O_2_ reduction	Antibody based/Sandwich	Amperometry (H_2_O_2_)	CALP	200 fg mL^−1^–50 ng mL^−1^/137 fg mL^−1^	Healthy human serum	[12]
ITO	In the presence of the target, DNA-walker-induced conformation switch to immobilize Pd/PCN-224/catalysis of NaBH_4_ oxidation	DNA-based/Direct	LSV (NaBH_4_)	DNA	100 fM–100 nM/33.6 fM	Human serum	[39]
GCE	rGO-TEPA/AuNPs/Strep/immobilization of thiol-DNA probe hemin-MOFs/PtNPs/signal amplification	DNA-based/Direct	Amperometry (H_2_O_2_)	*FGFR3* mutation gene	0.1 fM–1 nM/0.033 fM	Serum from patients in different gestation periods	[47]
Bare gold	2D-Zr-MOF/Electrode modifier ad for aptamer immobilization	Apasensor-based/Direct	EIS ([Fe(CN)_6_]^4−/3−^)	Mucin 1	0.001–0.5 ng·mL^−1^/0.12 pg mL^−1^	Human serum	[48]
	Co-MOF nanosheet array on nickel foam/glucose oxidation electrocatalysis	Nanozyme/Direct	Amperometry	Glucose	0.001–3 mM/1.3 nM	Human blood serum and fruit juice	[49]
-Multifunctional nanomaterials involving quantum dots (QDs)-							
GCE	Modification with iron magnetic nanoparticles with a core shell MOFs to immobilize the cAb and NiCd-QDs conjugated to DAb as electroactive non-enzymatic probe	Antibody based/Sandwich	DPV	PSA	1 pg mL^−1^–100 ng mL^−1^/0.45 pg mL^−1^	Human serum	[13]
GCE	PbS-QDs conjugated to DAb as a label	Antibody based/Sandwich	stripping of Pb(II) by SWASV	HER2	1–100 ng mL^−1^/0.28 ng mL^−1^	Spiked serum samples	[16]
SPCE array	CdSe/ZnS-QDs conjugated with anti-h-IgG as label	Antibody based/Sandwich	DPASV	Anti-tTG IgG	Up to 40 U mL^−1^/2.2 U mL^−1^		[20]
GCE	MWCNTs@PDA@AuNPs/Electrode modifier for immobilization of Con A Aptamer-DNA concatamer-QDs/recognizing probes	Aptamer-based/Sandwich	ASV	Cancer cells	1.0 × 10^2^–1.0 × 10^6^ cells mL^−1^/50 cells mL^−1^	Model cancer cells	[28]
GCE	Cat@AMQDs/Electrode modifier	Enzyme-based	Amperometry −0.1 V (H_2_O_2_)	CA125	50–300 µM/4.4 μM	Ovarian cancer serum	[50]
SPCE	core/shell CdSe@ZnS-QDs-Strep/signal tag	Antibody based/Sandwich	DPASV	HER2-ECD	10–150 ng mL^−1^/2.1 ng mL^−1^	spiked human serum samples	[51]
Bare gold	streptavidin-CdQD	DNA-based	SWASV	Telomerase activity	1 to 10^5^ cells	HeLa cells, HEK293T cells and MRC-5 cells	[52]
Bare gold	3-CdTeQD-DNA nanocomposite hybridizes with the cleaved DNA probe on the electrode after DNA probe-mRNA interaction and DSN	DNA-based	DPASV	mRNA of *BRCA1*	5 aM–5 fM/1.2 aM	Healthy human serum samples	[53]
GCE	AuNP/Electrode modifier for immobilization of two specific harpins PbS@ZIF-8-S1 and CdS@ZIF-8-S2/signaling tag	DNA-based/CHA for signal application	DPASV	Hemophilia A biomarkers: miR-1246 and miR-4521	miR-1246: 0.05 pM–1.0 mM miR-4521: 0.05 pM–1.0 mM	Human serum	[54]
*-*Two-dimensional (2D) transition metals-							
GCE	Ag@Ti_3_C_2_T_x_ nanocomposites as nanocarriers of AChE	Enzyme-based	DPV	Malathion	10^−14^–10^−8^ M/3.27 × 10^−15^ M	Tap water	[10]
GCE	MoS_2_-AuNPs as electrode modifier and nanoprobe for immobilizing the cAb and HRP-DAb respect.	Antibody based/Sandwich	DPV (H_2_O_2_ + *o*-PD)	CEA	10 fg mL^−1^–1 ng mL^−1^/1.2 fg mL^−1^	Spiked samples	[24]
SPE	MoS_2_-AuNPs-β-CD as electrode modifier and for immobilizing the MB-aptamer probe	Aptamer-based/Displacement	DPV (Fc-COOH)	OTA	0.1–50 nM/0.06 nM	-	[30]
CPE	PXA film functionalized MoS_2_ nanosheets/Electrode modifier for DNA immobilization and signal tag	DNA-based/Displacement	EIS	Circulating tumor DNA	1.0 × 10^–16^–1.0 × 10^−10^ mol L^−1^/1.8 × 10^–17^ mol L^−1^	PIK3CA gene in peripheral blood of patients with gastric carcinoma	[40]
GCE	AuNPs-MoS_2_ microcubes/Electrode modifier for biotin-cDNA DNS cleaves duplexes DNA-mRNA and mRNA is released	DNA-based/strep-AP	DPV FcM (redox mediator) TCEP (reducing reagent) and AAP (enzyme substrate)	miRNA-21	0.1 fM–0.1 pM/0.086 fM	Human serum samples breast from cancer patients	[55]
GCE	Av-MBs/Electrode modifier for cAb immobilization AuNP-FMC-WS_2_ nanocomposite/for DAb immobilization and signal tag	Antibody based/Sandwich	DPV	CA72-4	2–50 U L^−1^/0.6 U L^−1^	Human serum	[56]
GCE	TiO_2_–SnS_2_ nanocomposite/Electrode modifier for GOx immobilization	Enzyme-based	Amperometry −0.45 V	Glucose	0.008–1.13 and 1.13–5.53 mM/1.8 µM	Human serum samples	[57]
GCE	Ti_3_C_2_-MXene functionalized with APTES/Electrode modifier for cAb immobilization	Antibody based/Direct	CV ([Ru(NH_3_)_6_]^3+^)	CEA	1.0 × 10^−4^–2000 ng mL^−1^/1.8 × 10^−5^ ng mL^−1^	Spiked human sera sample	[58]
GCE	MoS_2_/Ti_3_C_2_ nanohybrids and AuNPs/Electrode modifier for probe RNA immobilization	DNA-based/Direct	DPV	miRNA-182	1 fM–0.1/0.43 fM	Serum samples	[59]

AAP: ascorbic acid 2-phosphate; AChE: acetylcholinesterase; AFP: alpha fetal protein; AP: alkaline phosphatase; AMO: methylamine oxidase; AMQDs: antimonene quantum dots; anti-tTG: anti-transglutaminase IgG antibodies; AO: alcohol oxidase; APOE4: apolipoprotein E4; APTES: 3-aminopropyl triethoxysilane; ATCl: acetylthiocholine chloride; AuBP: gold nanobipyramid; AuNRs: gold nanorods; AuNTs: gold nanotriangles; Av: avidin; BRCA1: breast cancer 1 gene mutation; CA72-4: carbohydrate antigen 72-4; cAb: capture antibody; CALP: calprotectin; ASV: anodic stripping voltammetry; Cat: catalase; CD: cyclodextrin; CEA: carcinoembryonic antigen; CHA: catalytic hairpin assembly; CHO: aldehyde groups; CILE: carbon ionic liquid electrode; Con A: concanavalin A; CPE: carbon paste electrode; CV: cyclic voltammetry; DAb: detector antibody; DNC: dendrimer nanocomposite; DPASV: differential pulse anodic stripping voltammetry; DPV: differential pulse voltammetry; dSPCE: dual screen-printed carbon electrode; DSN: double-strand specific nuclease; EIS: electrochemical impedance spectroscopy; FBThF: 4, 7-di (furan-2-yl) benzo thiadiazole; Fc: ferrocene; Fc-COOH: ferrocenecarboxylic acid; FcM: ferrocene methanol; GE: graphite electrode; GO: graphene oxide; GOx: glucose oxidase; GS-IL: ionic liquid modified graphene sheets; HER2: human epidermal growth factor receptor 2; HER2-ECD: extracellular domain of the human epidermal growth factor receptor 2; HRP: horseradish peroxidase; IL: ionic liquid; LOD: detection limit; LR: linear range; ITO: indium tin oxide; MB: methylene blue; MPBA: 4-mercaptophenylboronic acid; MWCNTs: multi-walled carbon nanotubes; NDD: neurodegenerative disorders; PAMAM: G4 polyamidoaminic dendrimers; *p*APP: *p*-aminophenyl phosphate; PCN-224: porphyrinic metal-organic framework; PDA: polydopamine; PGM: personal glucose meter; PPy: polypirrole; pRhNPs: porous rhodium nanoplates; PSA: prostate specific antigen; PtNPs: platinum nanoparticles; PXA: poly-xanthurenic acid; rHuEPO: recombinant human erythropoietin; SA: sodium alginate; SCCA: squamous cell carcinoma antigen; SPCE: screen-printed carbon electrode; SPE: screen-printed electrode; Strep: streptavidin; SWASV: square wave anodic stripping voltammetry; T4: tyroxine; TB: toluidine blue; TCA: trichloroacetic acid; TCEP: tris(carboxyethyl)phosphine; TCPP: tetrakis (4-carboxyphenyl) porphyrin; TDN: tetrahedral DNA nanostructure; TdT: terminal deoxynucleotidyl transferase; TEPA: tetraethylenepentamine; Thi: thionine; TSGF: tumor specific growth factor; VEGF: vascular endothelial growth factor.

**Table 2:** Electrochemical (bio)sensing strategies involving multifunctional carbon nanomaterials.

Electrode	Nanomaterial/Role	(Bio)sensing Approach/Format	Detection Technique	Target Analyte	LR/LOD	Sample	Ref.
-Magnetic carbon nanomaterials-							
CPE	m-CNTs@MIP/selective detection	Sensor	Voltammetry	Levofloxacin	0.003–0.440 μM/0.8 nM.	Spiked serum, urine	[78]
SPCE	m-MWCNTs/nanocarrier tag for Ab_2_	Sandwich-type immunosensor	Amperometry (H_2_O_2_ + HQ)	Fetuin	20–2000 pg mL^−1^/16 pg mL^−1^	Saliva	[79]
SPCE	AuNPs-Fe_3_O_4_-GS/electrode modifier	Aptasensor	SWV	CTCs	5–500 cells mL^−1^/3–4 cells mL^−1^	Whole blood	[80]
GCE	Pb^2+^@AuNPs-MWCNTs-Fe_3_O_4_/label for Ab2	Sandwich-type immunosensor	Amperometry (H_2_O_2_)	AFP	10 fg/mL–100 ng mL^−1^/3.33 fg mL^−1^	Spiked serum	[81]
AuE	Fe_3_O_4_/GO electrode modifier for avastin Ab_1_ immobilization	Label-free immunosensor	DPV [Fe(CN)_6_]^3−/4−^	VEGF	31.25–2000 pg mL^−1^	Plasma	[82]
GCE	Au@Ag/GS*-*Fe_3_O_4_/Cd^2+^/electrode modifier for immobilization of anti-IgG	Label-free immunosensor	Amperometry	IgG	5 fg mL^−1^–50 ng mL^−1^/2 fg mL^−1^	Serum	[83]
SPCE	Fe_3_O_4_@AuNPs/NGr	Aptamer	DPV	Leukemia cancer cells	10–10^6^ cell mL^−1^	Plasma	[84]
ITO	Fe_3_O_4_@PDA/rGO/electrode modifier for Ab_1_ immobilization	Sandwich-type immunosensor	CV	MC-LR	0.01–50 mg L^−1^/0.007 mg L^−^^1^	Water	[85]
GCE	GS-Fe_3_O_4_/Au@Ag/Ni^2+^/label for Ab_2_ loading	Sandwich-type immunosensor	Amperometry	CEA	0.1 pg/mL–100 ng/mL/0.0697 pg/mL	Serum	[86]
GCE	Fe_3_O_4_@AuNPs/rGO/electrode modifier for cortisol immobilization	Immunosensor/Direct competitive	DPV (H_2_O_2_/HRP)	Cortisol	0.1–1000 ng mL^−^^1^/0.05 ng mL^−^^1^	Serum	[87]
CPE	Fe_3_O_4_/rGO-PANHS/electrode modifier for BRCA1 5382 insC ssDNA immobilization	Label-free DNA biosensor	EIS	BRCA1 5382 insC mutation.	1.0 × 10^−^^18^–1.0 × 10^−^^8^ mol L^−^^1^/2.8 × 10^−^^19^ mol L^−^^1^	Spiked genome samples	[88]
GCE	CNP-L/CuONP/MWCNTs/Pe	Enzymatic biosensor	Amperometry	Triglycerides	0.001–0.05 g L^−^^1^/0.0032 g L^−^^1^ (triolein)	Serum	[89]
GCE	Fe_3_O_4_/CNTs/GO for aptamer immobilization	Label-free aptasensorr	DPV [Fe(CN)_6_]^3−/4−^	Diclofenac	100–1300 pM/ 33 pM	Ampoules	[90]
PtE	HRP/Fe_3_O_4_/Chit/rGO/electrode modifier	Enzymatic biosensor	CV	H_2_O_2_	up to 100 μM	-	[91]
SPCE	C@GNRs/electrode modifier for immobilization of ssDNA	DNA hybridization biosensor	CV Fe(CN)_6_^3−^	ssDNA	-	-	[92]
GCE	ZnFe_2_O_4_/α-Fe_2_O_3_/Gr/electrode modifier	Non-enzymatic	Amperometric	Glucose	1–10 mM	-	[93]
GCE	Fe_3_O_4_@TMU-21/MWCNTs/electrode modifier	Label-free immunosensor	Amperometric (H_2_O_2_)	HER2	1.0 pg mL^−^^1^–100 ng mL^−^^1^/0.3 pg mL^−^^1^	Serum	[94]
-Carbon nanozymes-							
GCE	ZnCr_2_O_4_/MWCNTs/electrode modifier	Enzyme-free sensor	Amperometry	H_2_O_2_	50 μM–34.8 mM/<0.11 μM	Lens cleaning solution	[95]
GCE	RuNPs/MWCNTs-Av/HRP mimic nanozyme	Modified electrode	Amperometry	H_2_O_2_	5.0 × 10^−7^ M–1.75 × 10^−3^ M/65 nM	-	[96]
GCE	Pt-DEN/CNTs/electrode modifier, HRP mimic nanozyme	Enzyme-free sensor	Amperometry	H_2_O_2_	3–400 μM/0.8 μM	H_2_O_2_ released from living cells	[97]
GCE	Fe_3_O_4_/CNTs/GO/electrode modifier, HRP mimic nanozyme	Enzyme-free sensor and enzyme biosensor (GOx)	CV (H_2_O_2_) Amperometry (glucose)	H_2_O_2_, glucose	0.01–0.50 mM (H_2_O_2_) 0.050–5.0 mM (glucose)	-	[98]
GCE	CDs/MWCNTs/electrode modifier, HRP mimic nanozyme	Enzyme-free sensor	Amperometry	H_2_O_2_	3.5 × 10^−6^–3.0 × 10^−4^ M/0.25 μM	H_2_O_2_ released from living cells, serum	[99]
GCE	Au-Ag/MWCNTs/electrode modifier	Enzyme-free sensor	CV	Gastric cancer cells-volatile biomarkers	up to 0.0025% (*v*/*v*)/0.3 ppb (3-octanone), up to 0.055% (*v*/*v*)/0.5 ppb (butanone)	MGC-803, GES-1 cells	[100]
AuE	GQDs/electrode modifier, Ab_1_ immobilization, HRP mimic nanozyme	Label-free immunosensor	Amperometry	*Yersinia enterecolitica*	6.23 × 10^2^–6.23 × 10^8^ cfu mL^−1^/5 cfu mL^−1^ (milk), 30 cfu mL^−1^ (serum)	Milk, serum	[101]
SPCE	GDQs/MWCNTs/Ab_2_ and HRP nanocarrier, HRP mimic nanozyme	Sandwich-type immunosensor	Amperometry (H_2_O_2_, HQ)	IL-13Rα2	2.7–100 ng mL^−1^/0.8 ng mL^−1^	Raw cellular lysates	[102]
GCE	PtPd/N-GQDs@Au/electrode modifier/catalytic activity towards H_2_O_2_	Label-free immunosensor	Amperometry (H_2_O_2_)	CEA	5 fg mL^−1^–50 ng mL^−1^/2 fg/mL	Spiked serum	[103]
AuE	GQDs/HRP mimic nanozyme	Enzyme-free sensor	Amperometry	H_2_O_2_	-	Human breast cancer MCF-7 cells	[104]
GCE	Au/OMCS/electrode modifier/xanthine oxidase mimics	Enzyme-free sensor	DPV	Xanthine	0.10–20 μM/0.006 μM	Spiked urine	[105]
-Multifunctional biomedical applications-							
AuE	rGO/insulin/Ni(OH)_2_/insulin releasing, glucose detection	Enzyme-free sensor	Amperometry	Glucose	5 μM–10 mM/~ 5 μM	-	[106]
GCE	rGO/PdNFs/electrode modifier	Enzyme-free sensor	Amperometry	Glucose	10–90 nM/8 nM	-	[107]
Au/PO	Gr/electrode modifier	Flexible microsensor	DPV	Dopamine	0.3 μM to 56.8 μM/0.11 μM	-	[108]
CPE	PVC/rGO/AuNPs/CNT/electrode modifier	Dual microcatheter	SWV	Propofol, fentanyl	25–125 μM (PPF), 10–50 nM (FTN)	Whole blood	[109]
GCE	rGO/MB-AuNPs/electrode modifier, immobilization of aptamer-Fc	Ratiometric aptasensor	SWV	VEGF	2–500 pg mL^−1^/0.1 pg mL^−1^	Serum	[110]
GCE	rGO/MB/electrode modifier	Ratiometric sensor	DPV	Cerebral ascorbic acid	0.5 μM–1000 μM/ 10 nM	Brain micro-dialysate	[111]
needle-type electrode	rGO/AuNCs/immobilization of ssDNA	Micro-aptasensor (hybridization)	DPV (MB)	Adenosine	0.1 nM–1 mM/~0.1 nM	In vivo (rat body)	[112]
micro-electrode array	rGOPtNPs/electrode modifier	Microsensor	DPV	Norepinephrine	-/0.08 μM	Brain silice secretion	[113]
GCE	rGO-MWCNTs /Chit/CQDs/aptamer immobilization	Label-free aptasensor	DPV, EIS [Fe(CN)_6_]^3−/4−^	Lysozyme	20 fmol L^−1^–10 nmol L^−1^/3.7 fmol L^−1^ (DPV); 10 fmol L^−1^–100 nmol L^−1^/1.9 fmol L^−1^ (EIS)	Spiked serum, urine	[114]
-Multifunctional carbon nanomaterials for signal amplification-							
SPCE	V-Phe-SWCNT (-HRP)/carrier tag for immobilization of Ab_2_	Sandwich-type immunosensor	Amperometry	TGF-β1	2.5–1000 pg mL^−^^1^/0.95 pg mL^−^^1^	Saliva	[115]
AuE	COOH-MWCNTs/AuNPs/immobilization of thionine and aptamer	Aptasensor	DPV	Tetracycline	0.1 nM–1 μM/0.06 nM	-	[116]
GCE	AuNPs-rGO/electrode modifier and Ab_1_ immobilization; SWCNTs-GQDs/carrier tag for Ab_2_ immobilization	Sandwich-type immunosensor	SWV (H_2_O_2_)	CEA	50–650 pg mL^−1^/5.3 pg mL^−1^	Spiked serum	[117]
GCE	Pt@Au-P-C60-rGO electrode modifier for immobilization of SH-aptamer and GOx	Label-free aptasensor	CV	Sulfadime-thoxime	10^−5^–50 ng mL^−1^/8.68 fg mL^−1^	Spiked milk	[118]
GCE	Pd/g-C_3_N_4_-CNTs/electrode modifier	Sensor	DPV	EE2	2.0 × 10^−6^ –1.5 × 10^−4^ M/5.0 × 10^−7^ M	Chicken and pig foodstuffs	[119]
GCE	GOx/Au/MXene/Nafion	Enzyme biosensor	Amperometry	glucose	0.1–18 mM/5.9 μM	-	[120]
AuNPs/ GCE	c-g-C_3_N_4_/carrier tag for Ab_2_ immobilizantion	Sandwich-type immunosensor	DPV	Procalcitonin	0.01–1.0 pg mL^−1^/2.0 fg mL^−1^	Plasma	[121]
dSPCE	GQDs/MWCNTs/HRP, carrier tag for Ab_2_ immobilization	Dual sandwich-type immunosensor	Amperometry (H_2_O_2_, HQ)	IL-13Rα2, CDH-17	4.92–100 ng mL^−1^/1.4 ng mL^−1^ (IL-13sRα2); 0.11–10 ng mL^−1^/0.03 ng mL^−1^ (CDH-17)	Lysates from breast and colorectal cancer cells	[122]
GCE	Chit@AB-MWCNTs/AuNPs/electrode modifier for immobilization of aptamer and specific binding to GO-ZEN Apt.	Aptasensor	DPV ([Fe(CN)_6_]^3−/4−^)	ZEN	10 fg mL^−1^–1 ng mL^−1^/ 3.64 fg mL^−1^	Corn oil and corn flour	[123]

AFP: alpha-fetoprotein; CDH-17: cadherin-17; CEA: carcinoembryonic antigen; C@GNRs: carbon-graphene nanoribbons; Chit: chitosan; CNP-L: lipase immobilized on magnetic nanoparticles; CQDs: carbon quantum dots; CTCs: circulating tumor cells; CPE: carbon paste electrode; CTCs: circulating tumor cells; CuONP: cupric oxide nanoparticles; CV: cyclic voltammetry; EE2: 17α-ethinylestradiol; EIS: electrochemical impedance spectroscopy; Fe_3_O_4_@TMU-21: magnetic framework TMU-21; GCE: glassy carbon electrode; GN: graphene nanosheet; GO: graphene oxide; GOx: glucose oxidase; GQDs: graphene quantum dots; GS: graphene sheets; HER2: human epidermal growth factor receptor 2; HRP: horseradish peroxidase; IL-13Rα2: receptor α2 of interleukin-13; ITO: indium tin oxide; MB: methylene blue; LOD: detection limit; m-CNTs: magnetic carbon nanotubes; MC-LR: microcystin-LR; OMCS: ordered mesoporous carbon/silica; NGr: nitrogen doped graphene; PANHS: polycyclic aromatic nitrogen heterocycles; Pe: pectin; SPCE: screen-printed carbon electrode; P-C_60_: poly(diallyldimethylammonium chloride)-fullerene C_60_; PdNFs, paladium nanoglowers; PO: poluolefin; SWV: square wave voltammetry; TGF-β1: transforming growth factor-β1; VEGF: vascular endothelial growth factor; ZEN: zerealenone.

**Table 3:** Reported mesoporous nanomaterials: types, names, and characteristic features [153].

Type		Names	Characteristic Features	Ref.
MSNs	Mobil Crystalline Materials (MCM)	MCM-48, MCM-41, and MCM-50	MCM-41: honeycomb-like structure with a pore diameter of 2.5–6 nm and an easily functionalized surface due to the presence of silanol groups. MCM-48 and MCM-50: cubic and lamella-like arrangement, respectively. MCM-41 and MCM-48: can be functionalized with amino groups	[154,162,163,164,165]
	Santa Barbara Amorphous (SBA)	SBA-11, SBA-12, and SBA 15, SBA-16	Pores of 4.6–30 nm and thicker silica walls. SBA-15: high surface to area ratio, porosity, uniform pore size distributions, with 30 nm average pore diameter and thermal stability.	[166,167,168,169]
	Korea advanced institute of science and technology (KIT)	KIT-5, KIT-6	KIT-6: composed of two interwoven mesoporous networks similar to that found in MCM-48 silica, but possessing much larger pore diameters of 5–12 nm.	[147,170]
	Center for research chemistry and catalysis (COK)	COK-12	Large-pore ordered mesoporous silica (OMS), analogous to the SBA-15, but synthesized in a more environmentally friendly way and exhibiting a shorter plate-like structure	[171]
	Folded sheets of mesoporous (FSM)	FSM-16	Narrowly distributed pore size of of 2.8 nm in diameter	[172]
	Fibrous silica nanospheres	KCC-1	Particles with extremely large surface area, highly stable, which tolerate high temperature, can be kept at room temperature for months without any changes in their properties and modified with various agents to change the surface properties	[173]
Mesoporous silica film (MSF)		MSF	Mesoporous that have hexagonal arrays with parallel cylindrical channels	[174,175,176,177]

**Table 4:** Electrochemical biosensors and biosensing methods involving multifunctional silica nanomaterials.

Electrode	Nanomaterial/Role	Biosensing Approach/Format	Detection Technique	Target Analyte	LR/LOD	Sample	Ref.
-MSNs-							
Disk AuE	Janus type Au-MSNs modified with Strep and HRP on the Au and MS faces, respectively/Signaling tags	Affinity-based/Direct	CV and EIE ([Fe(CN)_6_]^4-/3-^)	-	-	-	[185]
GCE coated with SWCNTs	Janus type Au-MSNs modified with GOx and HRP on the Au and MS faces, respectively/Electrode modifier	Enzyme-based	Amperometry (H_2_O_2_+ HQ)	Glucose	490 nM–600 μM/360 nM	Commercial soft drinks	[186]
SPCE	Janus type Au-MSNs modified with biotin thiol-modified anti-CEA DNA hairpin aptamer and HRP on the Au and mesoporous silica faces/Signaling tags and avidin-modified Fe_3_O_4_@SiO_2_ NPs/Solid support	Aptamer-based/Direct	Amperometry (H_2_O_2_ + HQ)	CEA	1–5000 ng mL^−1^/1.2 pM	Spiked human blood plasma	[183]
-	MB-loaded aptamer-gated MSNs (MCM-41)/Electrode modifiers	Aptamer-based/Direct	DPV (MB)	OTA	Up to 50 nM/0.003 nM	Doped wheat samples	[164]
AuNPs-SPCE	MB-loaded MSNs and capped with an avidin/imminobiotin stimulus-responsive gate-like ensemble/Nanocarriers of signaling elements	Aptamer-based/Direct	DPV (MB)	CEA	1.0 pg mL^−1^–160 ng mL^−1^/280 fg mL^−1^	Human serum samples (5-fold diluted)	[187]
-	Glucose-loaded DNAzyme-capped MSNs	Affinity-based/Direct	PGM	Pb^2+^	1.0 pM–0.7 nM/1 pM	Spiked drinking water	[188]
-	Glucose-loaded wrapping DNA-capped MSNs	Telomerase + dNTPs-assisted extension	PGM	Telomerase	100–5000 HeLa cells mL^−1^/80 HeLa cells mL^−1^	HeLa extracts	[189]
-	Glucose-loaded DNA-gated MSNs	DNA-based/Direct	PGM	Hg^2+^	0.1–80 nM/0.1 nM	Spiked tap water and lake water	[190]
-	Aptamer-capped MSNs with glucose loaded on GO nanosheets attached to the aptamer through π-stacking interactions	Aptamer-based/Displacement	PGM	AsO_3_^-^	0.01–100 ng mL^−1^/2.3 pg mL^−1^	River, lake, seawater and tap water	[179]
-	“Dual gates” aminated magnetic mesoporous silica nanocomposites loaded with glucose and bearing PDA-aptamer two-tier shells	Aptamer-based/Direct	PGM	AFB1	0.03–8 ng mL^−1^/0.02 ng mL^−1^	Pearl rice, maize and wheat	[191]
GCE	Au-MSNs/Electrode modifier	Aptamer-based/Direct	DPV ([Fe(CN)_6_]^4−/3−^)	Codein	10 pM–100 nM/3 pM	-	[156]
Graphite SPE	AuNPs/SBA-15@DABCO/Electrode modifier	Aptamer-based/Direct	DPV (hemin)	CAP	0.03–0.15 μM and 0.15–7.0 μM/4.0 nM	Spiked human blood serum	[169]
SPCE	AuNPs/Fe_3_O_4_@SiO_2_/DABCO/Electrode modifier	Aptamer-based/Direct	LSV (epirubicin)	Epirubicin	0.07 μM–1.0 μM and 1.0 μM–21.0 μM/0.04 μM	Spiked human blood serum	[152]
GCE	AMSNs (MCM-41)/Electrode modifier	Aptamer-based/Direct	DPV (electrocatalytic reduction of oxygen by hemin)	Hemin and Hb	Hemin and Hb: 1.0 × 10^−19^–1.0 × 10^−6^ M/Hemin: 7.5 × 10^−20^ M, Hb: 6.5 × 10^−20^ M	Blood (Hb)	[163]
GCE	AuNPs incorporated in AMSNs (MCM-41)/Electrode modifier	Aptamer-based/Direct	EIS ([Fe(CN)_6_]^4^^−/3^^−^)	CEA	1.0 × 10^−^^3^−100.0 ng mL^−1^ 9.8 × 10^−4^ ng mL^−1^	Human serum	[154]
GCE	GQDs-CS/KCC-1-NH_2_-Tb/Electrode modifier	Aptamer-based/Direct	DPV (TB)	AFB1	0.1 μM–1 fM/LQ = 10 fM	Spiked milk samples	[173]
GCE	MWCNTs-MSNs (MCM41)-Hb/Electrode modifier	Enzymatic-based (Hb DET)	Amperometry	NO_2_^-^, TCA	NO_2_^−^: 1.0 × 10^−^^7^–1.25 × 10^−^^4^ M/16 nM TCA: 5.0 × 10^−^^5^–2.7 × 10^−^^2^ M/3 μM	Spiked tap water	[165]
GCE	PAMAM–rGO/electrode modifier and mSiO_2_@MWCNT/nanocarriers of Thi, platinum nanoparticles (PtNPs), and hemin/G-quadruplex bioelectrocatalytic complex	Aptamer-based/Sandwich	DPV (H_2_O_2_)	Thrombin	0.0001–80 nM/50 fM	Spiked human serum	[178]
GCE modified with HDGMs	Au-MSNs modified with Thi/Signaling tags	Aptamer-based/Sandwich	DPV (Thi)	Thrombin	0.03 pM–0.018 μM/15 fM	Spiked fetal calf serum	[151]
GCE	TRSiNs doped with Thi/electrode modifier and GMSNs/nanocarriers of STR-BSA and HRP	Antibody-based/Competitive	DPV (H_2_O_2_+Thi)	STR	0.05–50 ng mL^−1^ 5 pg mL^−1^	Spiked samples (honey, milk, kidney, and muscle)	[192]
GO–AuNPs-modified GCE	Fe_3_O_4_@SiO_2_–NH_2_/Nanocarriers of Fc-COOH and DAb	Antibody-based/Sandwich	DPV (H_2_O_2_+Fc)	CEA	0.001 ng mL^−1^–80 ng mL^−1^/0.0002 ng mL^−1^	Spiked human serum samples	[182]
RGO-GCE	Mesoporous SiO_2_ decorated with DAb and CdSeTe@CdS quantum dots (QDs) or Ag nanoclusters (NCs)/nanocarriers of signaling elements	Antibody-based/Sandwich	ASV (Ag, Cd)	Bcl-2 and Bax	1–250 ng mL^−1^/∼0.5 fmol	Nilotinib-treated chronic myeloid leukemia K562 cells	[180]
AuNPs-GO modified GCE covered with MIPs for RhB	DNA-modified SiO_2_@AgNPs/Tracing tag	DNA-based/Sandwich	DPV (RhB)	*BRCA-1*	10 fM–100 nM/2.53 fM	Spiked human serum	[193]
-SiO_2_-SPAABs-							
GO-GCE	SiO_2_-SPAABs decorated with HRP and DAb/Nanocarriers	Antibody-based/Sandwich	DPV (H_2_O_2_ + ODP)	human IgG (HIgG)	100 pg mL^−1^–100 μg mL^−1^	Spiked serum samples	[194]
-Si MSs-							
AuNPs-GCE	Au-Si MSs modified with signal molecules (Zn^2+^) and antibodies (Ab)/Signaling tags	Mixed aptamer and antibody-based/Sandwich	SWV (Zn^2+^)	CRP	0.005–125 ng mL^−1^/0.0017 ng mL^−1^	Real serum samples	[195]
-MSF-							
Disk AuE	NH_2_-MSF/Electrode modifier	Biocatalytic-based	CV/EIS ([Fe(CN)_6_]^4−/3−^)	TGase activity (in the presence of *N*-benzyloxycarbonyl-L-glutaminylglycine) and indirectly of Pb^2+^ due to TGase activity irreversible inhibition	TGase activity: 0.4–185 μU mL^−1^ Pb^2+^: 4.0–500 μM	-	[196]
Disk AuE	DNAzyme-gated MSF/Electrode modifier	DNAzyme-based	SWV ([Fe(CN)_6_]^4^^−/3^^−^)	Cu^2+^, AA	Cu^2+^: 310 ppb–4.5 ppm/250 ppb AA: 1.0–120 μM/700 nM	Certified water samples (Cu^2+^) and Redoxon^®^ pills (AA)	[174]
Disk AuE	DNA-gated MSF/Electrode modifier	DNA-based biosensing approach	SWV ([Fe(CN)_6_]^4−/3−^)	*E. coli* 16S rRNA	Synthetic target DNA, a copy of partial region of the *E. coli* 16S rRNA gene (position 432–461 according to the 5′→3′ nucleotide sequence): 5–700 nM/ 2.5 nM	Raw *E. coli* lysate	[175]
Disk AuE	Aptamer-gated MSF/Electrode modifier	Aptamer-based biosensing approach	DPV ([Fe(CN)_6_]^4−/3−^)	PSA	1 to 300 ng mL^−1^/280 pg mL^−1^	Spiked artificial urine samples and blood serum from a healthy individual	[176]
AuE	AgNPs-MSF/Electrode modifier	Aptamer-based biosensing approach	DPV ([Fe(CN)_6_]^4−/3−^)	STR	1 fg mL^−1^–6.2 ng mL^−1^/0.33 fg mL^−1^	Spiked milk and blood serum samples	[177]

AA: ascorbic acid; AFB1: aflatoxin B1; AMSNs amino-functionalized MSNs; AMMS: aminated magnetic mesoporous silica nanocomposites; ASV: anodic stripping voltammetry; *BRCA-1*: breast cancer susceptibility gene; Bcl-2: B-cell lymphoma 2; Bax: Bcl-2-associated X protein; CAP: chloramphenicol; CEA: carcinoembryonic antigen; CRP: C-reactive protein; DET: direct electron transfer; DAb: detector antibody; DABCO: 1,4-diazabicyclo[2.2.2]octane; DPV: differential pulse voltammetry; *Escherichia coli*: *E. coli*; GCE: glassy carbon electrode; GO: graphene oxide; Fc: ferrocene; GMSNs: nanogold-assembled mesoporous silica; GQDs-CS: chitosan modified graphene quantum dot; Hb: hemoglobin; HDGMs: densely packed hierarchical dendritic gold microstructures; HIgG: human IgG; HQ: hydroquinone; HRP: horseradish peroxidase; LOD: detection limit; LQ: quantification limit; LSV: linear sweep voltammetry; KCC-1-NH_2_-Tb: dendritic fibrous nanosilica functionalized by amine groups; MIPs: molecularly imprinted polymers; MSF: mesoporous silica film; MWCNTs: multi-walled carbon nanotubes; MB: methylene blue; MSNs: mesoporous silica nanoparticles; Si MSs: silica microspheres; SWCNTs: single-walled carbon nanotubes; TB: toluidine blue; Hb: hemoglobin; MSNs: mesoporous silica nanoparticles; OPD: o-phenylenediamine; OTA: Ochratoxin A; PAMAM: poly(amidoamine); PDA: polydopamine; PSA: prostate specific antigen; PGM: personal glucose meter; RGO: reduced graphene oxide; RhB: rhodamine B; rRNA: ribosomal RNA; Si MSs: silica microspheres; SiO_2_-SPAABs: silica-poly(acrylic acid) brushes; STR: streptomycin; TB: toluidine blue; Thi: thionine; TRSiNs: three-dimensional redox-active organosilica nanostructures; SPCE: screen-printed carbon electrode; SPE: screen-printed electrode; SWV: square wave voltammetry; STR: streptomycin; TCA: trichloroacetic acid; TGase: transglutaminase.

## References

[1] Yoon J., Shin M., Lee T., Choi J.-W. (2020). Highly sensitive biosensors based on biomolecules and functional nanomaterials depending on the types of nanomaterials: A perspective review. Materials.

[2] Wongkaew N., Simsek M., Griesche C., Baeumner A.J. (2019). Functional nanomaterials and nanostructures enhancing electrochemical biosensors and lab-on-a-chip performances: Recent progress, applications, and future perspective. Chem. Rev..

[3] Cho I.-H., Kim D.H., Park S. (2020). Electrochemical biosensors: Perspective on functional nanomaterials for on-site analysis. Biomater. Res..

[4] Bezinge L., Suea-Ngam A., deMello A.J., Shih C.-J. (2020). Nanomaterials for molecular signal amplification in electrochemical nucleic acid biosensing: Recent advances and future prospects for point-of-care diagnostics. Mol. Syst. Des. Eng..

[5] Simón de Dios A., Díaz-García M.E. (2010). Multifunctional nanoparticles: Analytical prospects. Anal. Chim. Acta.

[6] Dimcheva N., Horozova E., Ivanov Y., Godjevrgova T. (2013). Self-assembly of acetylcholinesterase on gold nanoparticles electrodeposited on graphite. Cent. Eur. J. Chem..

[7] Niu Y., Liu J., Chen W., Yin C., Weng W., Li X., Wang X., Li G., Sun W.A. (2018). Direct electron transfer biosensor based on a horseradish peroxidase and gold nanotriangle modified electrode and electrocatalysis. Anal. Methods.

[8] Tang H., Cai D., Ren T., Xiong P., Liu Y., Gu H., Shi G. (2019). Fabrication of a low background signal glucose biosensor with 3D network materials as the electrocatalyst. Anal. Biochem..

[9] Guzsvány V., Anojčić J., Vajdle O., Radulović E., Madarász D., Kónya Z., Kalcher K. (2019). Amperometric determination of glucose in white grape and in tablets as ingredient by screen-printed electrode modified with glucose oxidase and composite of platinum and multiwalled carbon nanotubes. Food Anal. Methods.

[10] Jiang Y., Zhang X., Pei L., Yue S., Ma L., Zhou L., Huang Z., He Y., Gao J. (2018). Silver nanoparticles modified two-dimensional transition metal carbides as nanocarriers to fabricate acetylcholinesterase-based electrochemical biosensor. Chem. Eng. J..

[11] Chang H., Zhang H., Lv J., Zhang B., Wei W., Guo J. (2016). Pt NPs and DNAzyme functionalized polymer nanospheres as triple signal amplification strategy for highly sensitive electrochemical immunosensor of tumour marker. Biosens. Bioelectron..

[12] Dong L., Yin L., Tian G., Wang Y., Pei H., Wu Q., Cheng W., Ding S., Xia Q. (2020). An enzyme-free ultrasensitive electrochemical immunosensor for calprotectin detection based on PtNi nanoparticles functionalized 2D Cu metal organic framework nanosheets. Sens. Actuators B-Chem..

[13] Ehzari H., Amiri M., Safari M. (2020). Enzyme-free sandwich-type electrochemical immunosensor for highly sensitive prostate specific antigen based on conjugation of quantum dots and antibody on surface of modified glassy carbon electrode with core-shell magnetic metal-organic frameworks. Talanta.

[14] Feng J., Li Y., Li M., Li F., Han J., Dong Y., Chen Z., Wang P., Liu H., Wei Q. (2017). A novel sandwich-type electrochemical immunosensor for PSA detection based on PtCu bimetallic hybrid (2D/2D) rGO/g-C_3_N_4_. Biosens. Bioelectron..

[15] Hartati Y.W., Letelay L.K., Gaffar S., Wyantuti S., Bahti H.H. (2000). Cerium oxide-monoclonal antibody bioconjugate for electrochemical immunosensing of HER2 as a breast cancer biomarker. Sens. Biosens. Res..

[16] Lah Z.M.A.N.H., Ahmad S.A.A., Zaini M.S., Kamarudin M.A. (2019). An electrochemical sandwich immunosensor for the detection of HER2 using antibody-conjugated PbS quantum dot as a label. J. Pharm. Biomed. Anal..

[17] Li F., Li Y., Feng J., Dong Y., Wang P., Chen L., Chen Z., Liu H., Wei Q. (2017). Ultrasensitive amperometric immunosensor for PSA detection based on Cu_2_O@CeO_2_-Au nanocomposites as integrated triple signal amplification strategy. Biosens. Bioelectron..

[18] Li Y., Zhang Y., Li F., Feng J., Li M., Chen L., Dong Y. (2017). Ultrasensitive electrochemical immunosensor for quantitative detection of SCCA using Co_3_O_4_@CeO_2_-Au@Pt nanocomposite as enzyme-mimetic labels. Biosens. Bioelectron..

[19] Liu Y., He G., Liu H., Yin H., Gao F., Chen J., Zhang S., Yang B. (2020). Electrochemical immunosensor based on AuBP@Pt nanostructure and AuPd-PDA nanozyme for ultrasensitive detection of APOE4. RSC Adv..

[20] Martín-Yerga D., González-García M.B., Costa-García A. (2014). Electrochemical immunosensor for anti-tissue transglutaminase antibodies based on the in situ detection of quantum dots. Talanta.

[21] Razzino C.A., Serafín V., Gamella M., Pedrero M., Montero-Calle A., Barderas R., Calero M., Lobo A.O., Yáñez-Sedeño P., Campuzano S. (2020). An electrochemical immunosensor using gold nanoparticles-PAMAM-nanostructured screen-printed carbon electrodes for tau protein determination in plasma and brain tissues from Alzheimer patients. Biosens. Bioelectron..

[22] Serafín V., Razzino C.A., Gamella M., Pedrero M., Povedano E., Montero-Calle A., Barderas R., Calero M., Lobo A.O., Yáñez-Sedeño P. (2020). Disposable immunoplatforms for the simultaneous determination of biomarkers for neurodegenerative disorders using poly(amidoamine) dendrimer/gold nanoparticle nanocomposite. Anal. Bioanal. Chem..

[23] Shen Y., Shen G., Zhang Y. (2018). Voltammetric immunoassay for α-fetoprotein by using a gold nanoparticle/dendrimer conjugate and a ferrocene derived ionic liquid. Microchim. Acta.

[24] Su S., Sun Q., Wan L., Gu X., Zhu D., Zhou Y., Chao J., Wang L. (2019). Ultrasensitive analysis of carcinoembryonic antigen based on MoS_2_-based electrochemical immunosensor with triple signal amplification. Biosens. Bioelectron..

[25] Yu S., Zou G., Wei Q. (2016). Ultrasensitive electrochemical immunosensor for quantitative detection of tumor specific growth factor by using Ag@CeO_2_ nanocomposite as labels. Talanta.

[26] Das R., Dhiman A., Kapil A., Bansal V., Sharma T.K. (2019). Aptamer-mediated colorimetric and electrochemical detection of Pseudomonas aeruginosa utilizing peroxidase-mimic activity of gold NanoZyme. Anal. Bioanal. Chem..

[27] Fu X.-M., Liu Z.-J., Cai S.-X., Zhao Y.-P., Wu D.-Z., Li C.-Y., Chen J.-H. (2016). Electrochemical aptasensor for the detection of vascular endothelial growth factor (VEGF) based on DNA-templated Ag/Pt bimetallic nanoclusters. Chin. Chem. Lett..

[28] Liu H., Xu S., He Z., Deng A., Zhu J.J. (2013). Supersandwich cytosensor for selective and ultrasensitive detection of cancer cells using aptamer-DNS concatamer-Quantum Dots probes. Anal. Chem..

[29] Ou D., Sun D., Lin X., Liang Z., Zhong Y., Chen Z. (2019). A dual-aptamer-based biosensor for specific detection of breast cancer biomarker HER2 via flower-like nanozymes and DNA nanostructures. J. Mater. Chem. B.

[30] Wang Y., Ning G., Bi H., Wu Y., Liu G., Zhao Y. (2018). A novel ratiometric electrochemical assay for ochratoxin A coupling Au nanoparticles decorated MoS_2_ nanosheets with aptamer. Electrochim. Acta.

[31] Zhao R.-N., Feng Z., Zhao Y.-N., Jia L.-P., Ma R.-N., Zhang W., Shang L., Xue Q.-W., Wang H.-S. (2019). A sensitive electrochemical aptasensor for Mucin 1 detection based on catalytic hairpin assembly coupled with PtPdNPs peroxidase-like activity. Talanta.

[32] Jin X., Zhou L., Zhu B., Jiang X., Zhu N. (2018). Silver-dendrimer nanocomposites as oligonucleotide labels for electrochemical stripping detection of DNA hybridization. Biosens. Bioelectron..

[33] Liu L., Chang Y., Xia N., Peng P., Zhang L., Jiang M., Zhang J., Liu L. (2017). Simple, sensitive and label-free electrochemical detection of microRNAs based on the in situ formation of silver nanoparticles aggregates for signal amplification. Biosens. Bioelectron..

[34] Liu L., Xing Y., Zhang H., Liu R., Liu H., Xia N. (2014). Amplified voltammetric detection of glycoproteins using 4-mercaptophenylboronic acid/biotin-modified multifunctional gold nanoparticles as labels. Int. J. Nanomed..

[35] Liu L., Xia N., Liu H., Kang X., Liu X., Xue C., He X. (2014). Highly sensitive and label-free electrochemical detection of microRNAs based on triple signal amplification of multifunctional gold nanoparticles, enzymes and redox-cycling reaction. Biosens. Bioelectron..

[36] Ma X., Qian K., Ejeromedoghene O., Kandawa-Schulz M., Wang Y. (2020). Electrochemical detection of microRNA based on SA-PPy/AuNPs nanocomposite with the signal amplification through catalytic hairpin assembly reaction and the spontaneous catalytic reaction of Fe^3+^/Cu^2+^. Electrochim. Acta.

[37] Park S.Y., Kim J., Yim G., Jang H., Lee Y., Kim S.M., Park C., Lee M.-H., Lee T. (2020). Fabrication of electrochemical biosensor composed of multi-functional DNA/rhodium nanoplate heterolayer for thyroxine detection in clinical sample. Colloids Surf. B Biointerfaces.

[38] Tian L., Qian K., Qi J., Liu Q., Yao C., Song W., Wang Y. (2018). Gold nanoparticles superlattices assembly for electrochemical biosensor detection of microRNA-21. Biosens. Bioelectron..

[39] Yan T., Zhu L., Ju H., Lei J. (2018). DNA-Walker-Induced Allosteric Switch for Tandem Signal Amplification with Palladium Nanoparticles/Metal-Organic Framework Tags in Electrochemical Biosensing. Anal. Chem..

[40] Zhang W., Dai Z., Liu X., Yang J. (2018). High-Performance Electrochemical Sensing of Circulating Tumor DNA in Peripheral Blood Based on Poly-Xanthurenic Acid Functionalized MoS_2_ Nanosheets. Biosens. Bioelectron..

[41] Dimecheva N. (2020). Nanostructures of noble metals as functional materials in biosensors. Curr. Opin. Electrochem..

[42] Du D., Wang M., Cai J., Qin Y., Zhang A. (2010). One-step synthesis of multiwalled carbon nanotubes-gold nanocomposites for fabricating amperometric acetylcholinesterase biosensor. Sens. Actuators B-Chem..

[43] Zhang P., Sun T., Rong S., Zeng D., Yu H., Zhang Z., Chang D., Pan H. (2019). A sensitive amperometric AChE-biosensor for organophosphate pesticides detection based on conjugated polymer and Ag-rGO-NH_2_ nanocomposite. Bioelectrochemistry.

[44] Wang C., Liu C., Luo J., Tian Y., Zhou N. (2016). Direct electrochemical detection of kanamycin based on peroxidase-like activity of gold nanoparticles. Anal. Chim. Acta.

[45] Stasyuk N., Gayda G., Zakalskiy A., Zakalska O., Serkiz R., Gonchar M. (2019). Amperometric biosensors based on oxidases and PtRu nanoparticles as artificial peroxidase. Food Chem..

[46] Kim H.Y., Song J., Park K.S., Park H.G. (2020). Simple and label-free strategy for terminal transferase assay using a personal glucose meter. Chem. Commun..

[47] Chen J., Yu C., Zhao Y., Niu Y., Zhang L., Yu Y., Wu J., He J. (2017). A novel non-invasive detection method for the *FGFR3* gene mutation in maternal plasma for a fetal achondroplasia diagnosis based on signal amplification by hemin-MOFs/PtNPs. Biosens. Bioelectron..

[48] He L., Duan F., Song Y., Guo C., Zhao H., Tian J.-Y., Zhang Z., Liu C.-S., Zhang X., Wang P. (2017). 2D zirconium-based metal-organic framework nanosheets for highly sensitive detection of mucin 1: Consistency between electrochemical and surface plasmon resonance methods. 2D Mater..

[49] Li Y., Xie M., Zhang X., Liu Q., Lin D., Xu C., Xie F. (2019). Co-MOF nanosheet array: A high-performance electrochemical sensor for non-enzymatic glucose detection. Sens. Actuators. B Chem..

[50] Fatima B., Hussain D., Bashir S., Hussain H.T., Aslam R., Nawaz R., Rashid H.N., Bashir N., Majeed S., Ashiq M.N. (2020). Catalase immobilized antimonene quantum dots used as an electrochemical biosensor for quantitative determination of H_2_O_2_ from CA-125 diagnosed ovarian cancer samples. Mater. Sci. Eng. C.

[51] Freitas M., Neves M.M.P.S., Nouws H.P.A., Delerue-Matos C. (2020). Quantum dots as nanolabels for breast cancer biomarker HER2-ECD analysis in human serum. Talanta.

[52] Li C.-C., Hu J., Lu M., Zhang C.-Y. (2018). Quantum dot-based electrochemical biosensor for stripping voltammetric detection of telomerase at the single-cell level. Biosens. Bioelectron..

[53] Yang B., Zhang S., Fang X., Kong J. (2019). Double signal amplification strategy for ultrasensitive electrochemical biosensor based on nuclease and quantum dot-DNA nanocomposites in the detection of breast cancer 1 gene mutation. Biosens. Bioelectron..

[54] Rezaei H., Motovali-bashi M., Radfar S. (2019). An enzyme-free electrochemical biosensor for simultaneous detection of two hemophilia A biomarkers: Combining target recycling with quantum dots-encapsulated metal-organic frameworks for signal amplification. Anal. Chim. Acta.

[55] Shuai H.-L., Huang K.-J., Chen Y.-X., Fang L.-X., Jia M.-P. (2019). Au nanoparticles/hollow molybdenum disulfide microcubes based biosensor for microRNA-21 detection coupled with duplex-specific nuclease and enzyme signal amplification. Biosens. Bioelectron..

[56] Hong G., Chen R., Xu L., Lu X., Yang Z., Zhou G., Li L., Chen W., Peng H. (2020). One-pot ultrasonic synthesis of multifunctional Au nanoparticle ferrocene-WS_2_ nanosheet composite for the construction of an electrochemical biosensing platform. Anal. Chim. Acta.

[57] Yao P., Yu S., Shen H., Yang J., Min L., Yang Z., Zhu X. (2019). A TiO_2_–SnS_2_ nanocomposite as a novel matrix for the development of an enzymatic electrochemical glucose biosensor. New J. Chem..

[58] Kumar S., Lei Y., Alshareef N.H., Quevedo-Lopez M.A., Salama K.N. (2018). Biofunctionalized two-dimensional Ti_3_C_2_ MXenes for ultrasensitive detection of cancer biomarker. Biosens. Bioelectron..

[59] Liu L., Wei Y., Jiao S., Zhu S., Liu X. (2019). A novel label-free strategy for the ultrasensitive miRNA-182 detection based on MoS_2_/Ti_3_C_2_ nanohybrids. Biosens. Bioelectron..

[60] Golchin K., Golchin J., Ghaderi S., Alidadiani N., Eslamkhah S., Eslamkhah M., Davaran S., Akbarzadeh A. (2018). Gold nanoparticles applications: From artificial enzyme till drug delivery. Artif. Cells Nanomed. Biotechnol..

[61] Rick J., Tsai M.-C., Hwang B.J. (2015). Biosensors incorporating Bimetallic Nanoparticles. Nanomaterials.

[62] Gao L., Zhuang J., Nie L., Zhang J., Zhang Y., Gu N., Wang T., Feng J., Yang D., Perrett S. (2007). Intrinsic peroxidase-like activity of ferromagnetic nanoparticles. Nat. Nanotechnol..

[63] Yang Y., Mao Z., Huang W., Liu L., Li J., Li J., Wu Q. (2016). Redox enzyme-mimicking activities of CeO_2_ nanostructures: Intrinsic influence of exposed facets. Sci. Rep..

[64] Verma N., Kumar N. (2019). Synthesis and biomedical applications of copper oxide nanoparticles: An expanding horizon. ACS Biomater. Sci. Eng..

[65] Yang T., Fruergaard A.S., Winther A.K., Zelikin A.N., Chandrawati R. (2020). Zinc oxide particles catalytically generate nitric oxide from endogenous and exogenous products. Small.

[66] Mu J., Wang Y., Zhao M., Zhang L. (2012). Intrinsic peroxidase-like activity and catalase-like activity of Co_3_O_4_ nanoparticles. Chem. Commun..

[67] Yue Y., Wei H., Guo J., Yang Y. (2020). Ceria-based peroxidase-mimicking nanozyme with enhanced activity: A coordination chemistry strategy. Colloids Surf. A Physicochem. Eng. Asp..

[68] Singh S. (2016). Cerium oxide based nanozymes: Redox phenomenon at biointerfaces. Biointerphases.

[69] Zhao F., Sun T., Geng F., Chen P., Gao Y. (2019). Metal-Organic Frameworks-Based Electrochemical Sensors and Biosensors. Int. J. Electrochem. Sci..

[70] Ulhakim M.T., Rezki M., Dewi K.K., Abrori S.A., Harimurti S., Septiani N.L.W., Kurnia K.A., Setyaningsih W., Darmawan N., Yuliarto B. (2020). Review—Recent trend on two-dimensional metal-organic frameworks for electrochemical biosensor application. J. Electrochem. Soc..

[71] Sánchez A., Villalonga A., Martínez-García G., Parrado C., Villalonga R. (2019). Dendrimers as soft nanomaterials for electrochemical immunosensors. Nanomaterials.

[72] Kokkinos C., Economou A. (2017). Emerging trends in biosensing using stripping voltammetric detection of metal-containing nanolabels—A review. Anal. Chim. Acta.

[73] Bolotsky A., Butler D., Dong C., Gerace K., Glavin N.R., Muratore C., Robinson J.A., Ebrahim A. (2019). Two-dimensional materials in biosensing and healthcare: From in vitro diagnostics to optogenetics and beyond. ACS Nano.

[74] Wen W., Song Y., Yan X., Zhu C., Du D., Wang S., Asiri A.M., Lin Y. (2018). Recent advances in emerging 2D nanomaterials for biosensing and bioimaging applications. Mater. Today.

[75] Kalambate P.K., Gadhari N.S., Li X., Rao Z., Navale S.T., Shen Y., Patil V.R., Huang Y. (2019). Recent advances in MXene-based electrochemical sensors and biosensors. TrAC Trends Anal. Chem..

[76] Lorencova L., Bertok T., Filip J., Jerigova M., Velic D., Kasak P., Mahmoud K.A., Tkac J. (2018). Highly stable Ti_3_C_2_T_x_ (MXene)/Pt nanoparticles-modified glassy carbon electrode for H_2_O_2_ and small molecules sensing applications. Sens. Actuators B.

[77] Villalonga R., Villalonga M.L., Díez P., Pingarrón J.M. (2011). Decorating carbon nanotubes with polyethylene glycol-coated magnetic nanoparticles for implementing highly sensitive enzyme biosensors. J. Mater. Chem..

[78] Khoshsafar H., Bagheri H., Rezaei M., Shirzadmehr A., Hajian A., Sepehri Z. (2016). Magnetic carbon paste electrode modified with a high performance composite based on molecularly imprinted carbon nanotubes for sensitive determination of levofloxacin. J. Electrochem. Soc..

[79] Sánchez-Tirado E., González-Cortés A., Yáñez-Sedeño P., Pingarrón J.M. (2018). Magnetic multiwalled carbon nanotubes as nanocarrier tags for sensitive determination of fetuin in saliva. Biosens. Bioelectron..

[80] Dou B., Xu L., Jiang B., Yuan R., Xiang Y. (2019). Aptamer-functionalized and gold nanoparticle array-decorated magnetic graphene nanosheets enable multiplexed and sensitive electrochemical detection of rare circulating tumor cells in whole blood. Anal. Chem..

[81] Li F., Han J., Jiang L., Wang Y., Li Y., Dong Y., Wei Q. (2015). An ultrasensitive sandwich-type electrochemical immunosensor based on signal amplification strategy of gold nanoparticles functionalized magnetic multi-walled carbon nanotubes loaded with lead ions. Biosens. Bioelectron..

[82] Lin C.-W., Wei K.-C., Liao S.-S., Huang C.-Y., Sun C.-L., Wu P.J., Lu Y.J., Yang H.W., Ma C.C.M. (2015). A reusable magnetic graphene oxide-modified biosensor for vascular endothelial growth factor detection in cancer diagnosis. Biosens. Bioelectron..

[83] Li F., Li Y., Dong Y., Jiang L., Wang P., Liu Q., Liu H., Wei Q. (2016). An ultrasensitive label-free electrochemical immunosensor based on signal amplification strategy of multifunctional magnetic graphene loaded with cadmium ions. Sci. Rep..

[84] Khoshfetrat S.M., Mehrgardi M.A. (2017). Amplified detection of leukemia cancer cells using an aptamer-conjugated gold-coated magnetic nanoparticles on a nitrogen-doped graphene modified electrode. Bioelectrochem..

[85] He Z., Wei J., Gan C., Liu W., Liu Y. (2017). A rolling circle amplification signal-enhanced immunosensor for ultrasensitive microcystin-LR detection based on a magnetic graphene functionalized electrode. RSC Adv..

[86] Li Y., Zhang Y., Li F., Li M., Chen L., Dong Y., Wei Q. (2017). Sandwich-type amperometric immunosensor using functionalized magnetic graphene loaded gold and silver core-shell nanocomposites for the detection of carcinoembryonic antigen. J. Electroanal. Chem..

[87] Sun B., Gou Y., Ma Y., Zheng X., Bai R., Attia A., Abdelmoaty A., Hu F. (2017). Investigate electrochemical immunosensor of cortisol based on gold nanoparticles/magnetic functionalized reduced graphene oxide. Biosens. Bioelectron..

[88] Jahanbani S., Benvidi A. (2016). A novel electrochemical DNA biosensor based on a modified magnetic bar carbon paste electrode with Fe_3_O_4_NPs-reduced graphene oxide/PANHS nanocomposite. Mater. Sci. Eng. C.

[89] Di Tocco A., Robledo S.N., Osunac Y., Sandoval-Cortez J., Granero A.M., Vettorazzi N.R., Martínez J.L., Segura E.P., Ilinác A., Zona A. (2018). Development of an electrochemical biosensor for the determination of triglycerides in serum samples based on a lipase/magnetite-chitosan/copper oxide nanoparticles/multiwalled carbon nanotubes/pectin composite. Talanta.

[90] Azadbakht A., Derikvandi Z. (2018). Aptamer-based sensor for diclofenac quantification using carbon nanotubes and graphene oxide decorated with magnetic nanomaterials. J. Iran. Chem. Soc..

[91] Waifalkar P.P., Chougaleb A.D., Kolluc P., Patila P.S., Patile P.B. (2018). Thin film magnetic nanoparticle decorated graphene based electrochemical nanobiosensor for H_2_O_2_ sensing using HRP. Colloids Surf. B Biointerfaces.

[92] Rodríguez B.A.G., Pérez-Caro M., Alencar R.S., Souza Filho A.G., Aguiar A. (2020). Graphene nanoribbons and iron oxide nanoparticles composite as a potential candidate in DNA sensing applications. J. Appl. Phys..

[93] Neravathu D., Paloly A.R., Sajan P., Satheesh M., Bushiri M.J. (2020). Hybrid nanomaterial of ZnFe_2_O_4_/α-Fe_2_O_3_ implanted graphene for electrochemical glucose sensing application. Diam. Relat. Mater..

[94] Ehzari H., Samimi M., Safari M., Gholivand M.B. (2020). Label-free electrochemical immunosensor for sensitive HER2 biomarker detection using the core-shell magnetic metal-organic frameworks. J. Electroanal. Chem..

[95] Shahnavaz Z., Hamid S.B.A. (2017). Fabrication of a novel metal chromite—carbon nanotube composite for the highly efficient electrocatalytic reduction of hydrogen peroxide. Appl. Surf. Sci..

[96] Gallay P., Eguílaz M., Rivas G. (2020). Designing electrochemical interfaces based on nanohybrids of avidin functionalized-carbon nanotubes and ruthenium nanoparticles as peroxidase-like nanozyme with supramolecular recognition properties for site-specific anchoring of biotinylated residues. Biosens. Bioelectron..

[97] Liu J.-X., Ding S.-N. (2017). Non-enzymatic amperometric determination of cellular hydrogen peroxide using dendrimer-encapsulated Pt nanoclusters/carbon nanotubes hybrid composites modified glassy carbon electrode. Sens. Actuators B-Chem..

[98] Wang H., Li S., Si Y.M., Sun Z.Z., Li S.Y., Lin Y.H. (2014). Recyclable enzyme mimic of cubic Fe_3_O_4_ nanoparticles loaded on graphene oxide dispersed carbon nanotubes with enhanced peroxidase-like catalysis and electrocatalysis. J. Mater. Chem. B.

[99] Bai J., Sun C., Jiang X. (2016). Carbon dots-decorated multiwalled carbon nanotubes nanocomposites as a high-performance electrochemical sensor for detection of H_2_O_2_ in living cells. Anal. Bioanal. Chem..

[100] Zhang Y., Gao G., Liu H., Fu H., Fan J., Wang K., Chen Y., Li B., Zhang C., Zhi X. (2014). Identification of volatile biomarkers of gastric cancer cells and ultrasensitive electrochemical detection based on sensing interface of Au-Ag alloy coated MWCNTs. Theranostics.

[101] Savas S., Altintas Z. (2019). Graphene quantum dots as nanozymes for electrochemical sensing of *Yersinia enterocolitica* in milk and human serum. Materials.

[102] Serafín V., Valverde A., Martínez-García G., Martínez-Periñán E., Comba F., Garranzo-Asensio M., Barderas R., Yáñez-Sedeño P., Campuzano S., Pingarrón J.M. (2019). Graphene quantum dots-functionalized multi-walled carbon nanotubes as nanocarriers in electrochemical immunosensing. Determination of IL-13 receptor α2 in colorectal cells and tumor tissues with different metastatic potential. Sens. Actuators B..

[103] Yang Y., Liu Q., Liu Y., Chui J., Liu H., Wang P., Li Y., Chen L., Zhao Z., Dong Y. (2017). A novel label-free electrochemical immunosensor based on functionalized nitrogen-doped graphene quantum dots for carcinoembryonic antigen detection. Biosens. Bioelectron..

[104] Zhang Y., Wu C., Zhou X., Wu X., Yang Y., Wu H., Guo S., Zhang J. (2013). Graphene quantum dots/gold electrode and its application in living cell H_2_O_2_ detection. Nanoscale.

[105] Wang Y., Zhao H., Song H., Dong J., Xu J. (2020). Monodispersed gold nanoparticles entrapped in ordered mesoporous carbon/silica nanocomposites as xanthine oxidase mimic for electrochemical sensing of xanthine. Microchim. Acta.

[106] Belkhalfa H., Teodorescu F., Quéniat G., Coffinier Y., Dokhan N., Sam S., Abderrahmani A., Boukherroub R., Szunerits S. (2016). Insulin impregnated reduced graphene oxide/Ni(OH)_2_ thin films for electrochemical insulin release and glucose sensing. Sens. Actuators B-Chem..

[107] He Y., Cao W., Cong C., Zhang X., Luo L., Li L., Cui H., Gao D. (2019). Rationally designed multifunctional carbon−palladium nanohybrids for wide applications: From electrochemical catalysis/nonenzymatic sensor to photothermal tumor therapy. ACS Sustain. Chem. Eng..

[108] He W., Liu R., Zhou P., Liu Q., Cui T. (2020). Flexible micro-sensors with self-assembled graphene on a polyolefin substrate for dopamine detection. Biosens. Bioelectron..

[109] Moonla C., Goud K.Y., Teymourian H., Tangkuaram T., Ingrande J., Suresh P., Wang J. (2020). An integrated microcatheter-based dual-analyte sensor system for simultaneous, real-time measurement of propofol and fentanyl. Talanta.

[110] Ni S., Shen Z., Zhang P., Liu G. (2020). Enhanced performance of an electrochemical aptasensor for real-time detection of vascular endothelial growth factor (VEGF) by nanofabrication and ratiometric measurement. Anal. Chim. Acta.

[111] Jiang Y., Xiao X., Li C., Luo Y., Chen S., Shi G., Han K., Gu H. (2020). Facile ratiometric electrochemical sensor for in vivo/online repetitive measurements of cerebral ascorbic acid in brain microdiaysate. Anal. Chem..

[112] Zhang D., Ma J., Meng X., Xu Z., Zhang J., Fang Y., Guo Y. (2019). Electrochemical aptamer-based microsensor for real-time monitoring of adenosine in vivo. Anal. Chim. Acta.

[113] Wang L., Song Y., Zhang Y., Xu S., Xu H., Wang M., Wang Y., Cai X. (2017). A microelectrode array electrodeposited with reduced graphene oxide and Pt nanoparticles for norepinephrine and electrophysiological recordings. J. Micromech. Microeng..

[114] Rezaei B., Jamei H.R., Ensafi A.A. (2018). An ultrasensitive and selective electrochemical aptasensor based on rGO-MWCNTs/Chitosan/carbon quantum dot for the detection of lysozyme. Biosens. Bioelectron..

[115] Sánchez-Tirado E., Arellano L.M., González-Cortés A., Yáñez-Sedeño P., Langa F., Pingarrón J.M. (2017). Viologen-functionalized single-walled carbon nanotubes as carrier nanotags for electrochemical immunosensing. Application to TGF-β1 cytokine. Biosens. Bioelectron..

[116] He B.S., Yan S.S. (2018). Electrochemical aptasensor based on aptamer complimentary strand conjugate and thionine for sensitive detection of tetracycline with multiwalled carbon nanotubes and gold nanoparticles amplification. Anal. Methods.

[117] Luo Y., Wang Y., Yan H., Wu Y., Zhu C., Du D., Lin Y. (2018). SWCNTs@GQDs composites as nanocarriers for enzyme-free dual signal amplification electrochemical immunoassay of cancer biomarker. Anal. Chim. Acta.

[118] You H., Mu Z., Zhao M., Zhou J., Chen Y., Bai L. (2019). Voltammetric aptasensor for sulfadimethoxine using a nanohybrid composed of multifunctional fullerene, reduced graphene oxide and Pt@Au nanoparticles, and based on direct electron transfer to the active site of glucose oxidase. Microchim. Acta.

[119] Zheng Z., Wang M., Shi X., Wang C. (2019). Palladium nanoparticles/graphitic carbon nitride nanosheets-carbon nanotubes as a catalytic amplification platform for the selective determination of 17α-ethinylestradiol in feedstuffs. Sci. Rep..

[120] Rakhi R.B., Nayak P., Xia C., Alshareef H.N. (2016). Novel amperometric glucose biosensor based on MXene nanocomposite. Sci. Rep..

[121] Medetalibeyoglu H., Beytur M., Akyıldırım O., Atar N., Yola M.L. (2020). Validated electrochemical immunosensor for ultra-sensitive procalcitonin detection: Carbon electrode modified with gold nanoparticles functionalized sulfur doped MXene as sensor platform and carboxylated graphitic carbon nitride as signal amplification. Sens. Actuators B-Chem..

[122] Serafín V., Valverde A., Garranzo-Asensio M., Barderas R., Campuzano S., Yáñez-Sedeño P., Pingarrón J.M. (2019). Simultaneous amperometric immunosensing of the metastasis-related biomarkers IL-13Rα2 and CDH-17 by using grafted screen-printed electrodes and a composite prepared from quantum dots and carbon nanotubes for signal amplification. Microchim. Acta.

[123] Mu Z., Ma L., Wang J., Zhou J., Yuan Y., Bai L. (2020). A target-induced amperometic aptasensor for sensitive zearalenone detection by CS@AB-MWCNTs nanocomposite as enhancers. Food Chem..

[124] Garg B., Bisht T. (2016). Carbon nanodots as peroxidase nanozymes for biosensing. Molecules.

[125] Huang Y., Ren J., Qu X. (2019). Nanozymes: Classification, catalytic mechanisms, activity regulation, and applications. Chem. Rev..

[126] Mahmudunnabi R.G., Farhana F.Z., Kashaninejad N., Firoz S.H., Shim Y.-B., Shiddiky M.J.A. (2020). Nanozyme-based electrochemical biosensors for disease biomarker detection. Analyst.

[127] Liang M., Yan X. (2019). Nanozymes: From new concepts, mechanisms, and standards to applications. Acc. Chem. Res..

[128] Sun H., Zhou Y., Ren J., Qu X. (2018). Carbon Nanozymes: Enzymatic Properties, Catalytic Mechanism, and Applications. Angew. Chem. Int. Ed..

[129] Cui R., Han Z., Zhu J.J. (2011). Helical carbon nanotubes: Intrinsic peroxidase catalytic activity and its application for biocatalysis and biosensing. Chem. Eur. J..

[130] Wang H., Jiang H., Wang S., Shi W.B., He J.C., Liu H., Huang Y.M. (2014). Fe_3_O_4_-MWCNT magnetic nanocomposites as efficient peroxidase mimic catalysts in a Fenton-like reaction for water purification without pH limitation. RSC Adv..

[131] Ye H., Mohar J., Wang Q., Catalano M., Kim M.J., Xia X. (2016). Peroxidase-like properties of ruthenium nanoframes. Sci. Bull..

[132] Nekoueian K., Amiri M., Sillanpää M., Marken F., Boukherroub R., Szunerits S. (2019). Carbon-based quantum particles: An electroanalytical and biomedical perspective. Chem. Soc. Rev..

[133] Hu Y., Gao X.J., Zhu Y., Muhammad F., Tan S., Cao W., Lin S., Jin Z., Gao X., Wei H. (2018). Nitrogen-doped carbon nanomaterials as highly active and specific peroxidase mimics. Chem. Mater..

[134] Fan K., Xi J., Fan L., Wang P., Zhu C., Tang Y., Xu X., Liang M., Jiang B., Yan X. (2018). In vivo guiding nitrogen-doped carbon nanozyme for tumor catalytic therapy. Nat. Commun..

[135] Raphey V.R., Henna T.K., Nivitha K.P., Mufeedha P., Sabu C., Pramod K. (2019). Advanced biomedical applications of carbon nanotube. Mat. Sci. Eng. C.

[136] Raghav S., Painuli R., Kumar D. (2017). Multifunctional nanomaterials for multifaceted applications in biomedical arena. Int. J. Pharmacol..

[137] Saleemi M.A., Kong Y.L., Yong P.V.C., Wong E.H. (2020). An overview of recent development in therapeutic drug carrier system using carbon nanotubes. J. Drug Deliv. Sci. Technol..

[138] Zhou Y., Chen C., Zhao J., Fei J., Ding Y., Cai Y. (2016). Reversible switched detection of dihydroxybenzenes using a temperature-sensitive electrochemical sensing film. Electrochim. Acta.

[139] Wu X., Bai X., Ma Y., Wei J., Peng J., Shi K., Yao H. (2018). Construction of multiple switchable sensors and logic gates based on carboxylated multi-walled carbon nanotubes/poly(*N*,*N*-diethylacrylamide). Sensors.

[140] Ma Y., Hou S., Xue S., Wu X., Yao H., Shi K., Ma W., Zuo L., Yao H. (2020). The fabrication of multiple stimuli-responsive film electrode and logic gates of rutin. J. Electrochem. Soc..

[141] Shervedani R.K., Foroushani M.S., Kefayat A., Torabi M., Rahsepar F.R. (2018). Construction and characterization of a theranostic system based on graphene/manganese chelate. Biosens. Bioelectron..

[142] Foroushani M.S., Shervedani R.K., Kefayat A., Torabi M., Ghahremani F., Yaghoobi F. (2019). Folate-graphene chelate manganese nanoparticles as a theranostic system for colon cancer MR imaging and drug delivery: In-vivo examinations. J. Drug Deliv. Sci. Technol..

[143] Lee D., Kim Y.H., Park S. (2016). Enzyme electrode platform using methyl viologen electrochemically immobilized on carbon materials. J. Electrochem. Soc..

[144] Chan M.-H., Liu R.-S., Hsiao M. (2019). Graphitic carbon nitride-based nanocomposites and their biological applications: A review. Nanoscale.

[145] Kresge C., Leonowicz M., Roth W.J., Vartuli J., Beck J. (1992). Ordered mesoporous molecular sieves synthesized by a liquid-crystal template mechanism. Nature.

[146] Narayan R., Nayak U., Raichur A., Garg S. (2018). Mesoporous silica nanoparticles: A comprehensive review on synthesis and recent advances. Pharmaceutics.

[147] Hasanzadeh M., Shadjou N., de la Guardia M., Eskandani M., Sheikhzadeh P. (2012). Mesoporous silica-based materials for use in biosensors. TrAC Trends Anal. Chem..

[148] Sancenón F., Pascual L., Oroval M., Aznar E., Martínez-Máñez R. (2015). Gated silica mesoporous materials in sensing applications. Chem. Open.

[149] Kordasht H.K., Pazhuhi M., Pashazadeh-Panahi P., Hasanzadeh M., Shadjou N. (2020). Multifunctional aptasensors based on mesoporous silica nanoparticles as an efficient platform for bioanalytical applications: Recent advances. TrAC Trends Anal. Chem..

[150] Feyen M., Weidenthaler C., Schüth F., Lu A.-H. (2010). Regioselectively controlled synthesis of colloidal mushroom nanostructures and their hollow derivatives. J. Am. Chem. Soc..

[151] Tang J., Tang D., Niessner R., Knopp D., Chen G. (2012). Hierarchical dendritic gold microstructure-based aptasensor for ultrasensitive electrochemical detection of thrombin using functionalized mesoporous silica nanospheres as signal tags. Anal. Chim. Acta.

[152] Huang L., Yang X., Qi C., Niu X., Zhao C., Zhao X., Shangguan D., Yang Y. (2013). A label-free electrochemical biosensor based on a DNA aptamer against codeine. Anal. Chim. Acta.

[153] Su M., Liu Y., Zhang Y., Wang Z., Li Y., He P. (2018). Robust and underwater superoleophobic coating with excellent corrosion and biofouling resistance in harsh environments. Appl. Surf. Sci..

[154] Shekari Z., Zare H.R., Falahati A. (2017). Developing an impedimetric aptasensor for selective label–free detection of CEA as a cancer biomarker based on gold nanoparticles loaded in functionalized mesoporous silica films. J. Electrochem. Soc..

[155] Wu S.H., Hung Y., Mou C.Y. (2011). Mesoporous silica nanoparticles as nanocarriers. Chem. Commun..

[156] Castillo R.R., Baeza A., Vallet-Regí M. (2017). Recent applications of the combination of mesoporous silica nanoparticles with nucleic acids: Development of bioresponsive devices, carriers and sensors. Biomater. Sci..

[157] Walcarius A. (2018). Silica-based electrochemical sensors and biosensors: Recent trends. Curr. Op. Electrochem..

[158] Jiménez-Falcao S., Villalonga A., Arévalo-Villena M., Briones-Pérez A., Martínez-Máñez R., Martínez-Ruiz P., Villalonga R. (2020). Enzyme-controlled mesoporous nanosensor for the detection of living *Saccharomyces cerevisiae*. Sens. Actuators B-Chem..

[159] Slowing I.I., Trewyn B.G., Giri S., Lin V.S.-Y. (2007). Mesoporous silica nanoparticles for drug delivery and biosensing applications. Adv. Funct. Mater..

[160] Xing Y. (2004). Synthesis and electrochemical characterization of uniformly dispersed high loading Pt nanoparticles on sonochemically-treated carbon nanotubes. J. Phys. Chem. B.

[161] Yang M., Li H., Javadi A., Gong S. (2010). Multifunctional mesoporous silica nanoparticles as labels for the preparation of ultrasensitive electrochemical immunosensors. Biomaterials.

[162] Miyahara M., Vinu A., Ariga K. (2007). Adsorption myoglobin over mesoporous silica molecular sieves: Pore size effect and pore-filling model. Mater. Sci. Eng. C.

[163] Shekari Z., Zare H.R., Falahati A. (2017). An ultrasensitive aptasensor for hemin and hemoglobin based on signal amplification via electrocatalytic oxygen reduction. Anal. Biochem..

[164] Muthamizh S., Ribes À., Anusuyajanakiraman M., Narayanan V., Soto J., Martínez-Máñez R., Aznar E. (2017). Implementation of oligonucleotide-gated supports for the electrochemical detection of Ochratoxin, A. Supramol. Chem..

[165] Eguílaz M., Villalonga R., Rivas G. (2018). Electrochemical biointerfaces based on carbon nanotubes-mesoporous silica hybrid material: Bioelectrocatalysis of hemoglobin and biosensing applications. Biosens. Bioelectron..

[166] Trewyn B.G., Giri S., Slowing I.I., Lin V.S.-Y. (2007). Mesoporous silica nanoparticle based controlled release, drug delivery, and biosensor systems. Chem. Commun..

[167] Hudson S.P., Padera R.F., Langer R., Kohane D.S. (2008). The biocompatibility of mesoporous silicates. Biomaterials.

[168] Øye G., Sjöblom J., Stöcker M. (2001). Synthesis, characterization and potential applications of new materials in the mesoporous range. Adv. Colloid Interface Sci..

[169] Hashkavayi A.B., Raoof J.B., Azimi R., Ojani R. (2016). Label-free and sensitive aptasensor based on dendritic gold nanostructures on functionalized SBA-15 for determination of chloramphenicol. Anal. Bioanal. Chem..

[170] Kleitz F., Liu D., Anilkumar G.M., Park I.-S., Solovyov L.A., Shmakov A.N., Ryoo R. (2003). Large cage face-centered-cubic Fm3m mesoporous silica: Synthesis and structure. J. Phys. Chem. B.

[171] Nandiyanto A.B.D., Kim S.-G., Iskandar F., Okuyama K. (2009). Synthesis of spherical mesoporous silica nanoparticles with nanometer-size controllable pores and outer diameters. Microporous Mesoporous Mater..

[172] Tozuka Y., Wongmekiat A., Kimura K., Moribe K., Yamamura S., Yamamoto K. (2005). Effect of pore size of FSM-16 on the entrapment of flurbiprofen in mesoporous structures. Chem. Pharm. Bull..

[173] Kordasht H.K., Moosavy M.-H., Hasanzadeh M., Soleymani J., Mokhtarzadeh A. (2019). Determination of Aflatoxin M1 using aptamer based biosensor on the surface of dendritic fibrous nano-silica functionalized by amine groups. Anal. Methods.

[174] Saadaoui M., Fernández I., Sánchez A., Díez P., Campuzano S., Raouafi N., Pingarrón J.M., Villalonga R. (2015). Mesoporous silica thin film mechanized with a DNAzyme-based molecular switch for electrochemical biosensing. Electrochem. Commun..

[175] Saadaoui M., Fernández I., Luna G., Díez P., Campuzano S., Raouafi N., Sánchez A., Pingarrón J.M., Villalonga R. (2016). Label-free electrochemical genosensor based on mesoporous silica thin film. Anal. Bioanal. Chem..

[176] Argoubi W., Sánchez A., Parrado C., Raouafi N., Villalonga R. (2018). Label-free electrochemical aptasensing platform based on mesoporous silica thin film for the detection of prostate specific antigen. Sens. Actuators B Chem..

[177] Roushani M., Ghanbari K. (2019). An electrochemical aptasensor for streptomycin based on covalent attachment of the aptamer onto a mesoporous silica thin film-coated gold electrode. Microchim. Acta.

[178] Zhang J., Chai Y., Yuan R., Yuan Y., Bai L., Xie S. (2013). A highly sensitive electrochemical aptasensor for thrombin detection using functionalized mesoporous silica@multiwalled carbon nanotubes as signal tags and DNAzyme signal amplification. Analyst.

[179] Zhou Q., Tang D. (2018). Graphene oxide-gated mesoporous silica nanocontainers using aptamers for arsenite detection with glucometer readout. J. Mater. Chem. B.

[180] Zhou S., Wang Y., Zhu J.J. (2016). Simultaneous detection of tumor cell apoptosis regulators Bcl-2 and Bax through a dual-signal-marked electrochemical immunosensor. ACS Appl. Mater. Interfaces.

[181] Hashkavayi A.B., Raoof J.B. (2017). Design an aptasensor based on structure-switching aptamer on dendritic gold nanostructures/Fe_3_O_4_@SiO_2_/DABCO modified screen printed electrode for highly selective detection of epirubicin. Biosens. Bioelectron..

[182] Bagheri E., Ansari L., Sameiyan E., Abnous K., Taghdisi S.M., Ramezani M., Alibolandi M. (2020). Sensors design based on hybrid gold-silica nanostructures. Biosens. Bioelectron..

[183] Feng T., Qiao X., Wang H., Sun Z., Hong C. (2016). A sandwich-type electrochemical immunosensor for carcinoembryonic antigen based on signal amplification strategy of optimized ferrocene functionalized Fe_3_O_4_@SiO_2_ as labels. Biosens. Bioelectron..

[184] Paniagua G., Villalonga A., Eguílaz M., Vegas B., Parrado C., Rivas G., Díez P., Villalonga R. (2019). Amperometric aptasensor for carcinoembryonic antigen based on the use of bifunctionalized Janus nanoparticles as biorecognition signaling element. Anal. Chim. Acta.

[185] Sánchez A., Díez P., Martínez-Ruíz P., Villalonga R., Pingarrón J.M. (2013). Janus Au-mesoporous silica nanoparticles as electrochemical biorecognition-signaling system. Electrochem. Commun..

[186] Boujakhrout A., Sánchez E., Díez P., Sánchez A., Martínez-Ruiz P., Parrado C., Pingarrón J.M., Villalonga R. (2015). Single-walled carbon nanotubes/Au–mesoporous silica Janus nanoparticles as building blocks for the preparation of a bienzyme biosensor. ChemElectroChem.

[187] Jiménez-Falcao S., Parra-Nieto J., Pérez-Cuadrado H., Martínez-Máñez R., Martínez-Ruiz P., Villalonga R. (2019). Avidin-gated mesoporous silica nanoparticles for signal amplification in electrochemical. Electrochem. Commun..

[188] Fu L., Zhuang J., Lai W., Que X., Lu M., Tang D. (2013). Portable and quantitative monitoring of heavy metal ions using DNAzyme-capped mesoporous silica nanoparticles with a glucometer readout. J. Mater. Chem. B.

[189] Wang Y., Lu M., Zhu J., Tian S. (2014). Wrapping DNA-gated mesoporous silica nanoparticles for quantitative monitoring of telomerase activity with glucometer readout. J. Mater. Chem. B.

[190] Liang X., Wang L., Wang D., Zeng L., Fang Z. (2016). Portable and quantitative monitoring of mercury ions using DNA-gated mesoporous silica nanoparticles using a glucometer readout. Chem. Commun..

[191] Wang L., Zhu F., Chen M., Xiong Y., Zhu Y., Xie S., Liu Q., Yang H., Chen X. (2019). Development of a “dual gates” locked, target-triggered nanodevice for point-of-care testing with a glucometer readout. ACS Sens..

[192] Liu B., Zhang B., Cui Y., Chen H., Gao Z., Tang D. (2011). Multifunctional goldesilica nanostructures for ultrasensitive electrochemical immunoassay of streptomycin residues. ACS Appl. Mater. Interfaces.

[193] You M., Yang S., Tang W., Zhang F., He P. (2018). Molecularly imprinted polymers-based electrochemical DNA biosensor for the determination of *BRCA-1* amplified by SiO_2_@Ag. Biosens. Bioelectron..

[194] Zhao Y., Zheng Y., Kong R., Xia L., Qu F. (2016). Ultrasensitive electrochemical immunosensor based on horseradish peroxidase (HRP)-loaded silica-poly(acrylic acid) brushes for protein biomarker detection. Biosens. Bioelectron..

[195] Wang J., Guo J., Zhang J., Zhang W., Zhang Y. (2017). RNA aptamer-based electrochemical aptasensor for C-reactive protein detection using functionalized silica microspheres as immunoprobes. Biosens. Bioelectron..

[196] Fernández I., Sánchez A., Díez P., Martínez-Ruiz P., Di Pierro P., Porta R., Villalonga R., Pingarrón J.M. (2014). Nanochannel-based electrochemical assay for transglutaminase activity. Chem. Commun..

[197] Aznar E., Oroval M., Pascual L., Murguia J.R., Martínez-Mánez R., Sancenón F. (2016). Gated materials for on-command release of guest molecules. Chem. Rev..

[198] Parvanian S., Mostafavi S.M., Aghashiri M. (2017). Multifunctional nanoparticle developments in cancer diagnosis and treatment. Sens. Biosens. Res..

[199] Mazzola L. (2003). Commercializing nanotechnology. Nat. Biotechnol..

